# Mitochondrial Phylogenomics and Genome Evolution in Anura: Insights From Structure and Gene Order Rearrangements

**DOI:** 10.1002/ece3.73370

**Published:** 2026-03-30

**Authors:** Jiaoying He, Zike Li, Qingya Yang, Mengyao Zhu, Baiyun Xue, Yinmeng Hou, Ganggang Yang, Xiaohong Chen, Zhuo Chen

**Affiliations:** ^1^ The Observation and Research Field Station of Taihang Mountain Forest Ecosystems of Henan Province, College of Life Sciences Henan Normal University Xinxiang China

**Keywords:** Anura, divergence time, evolution, mitogenome, phylogeny, rearrangement

## Abstract

Mitochondrial genome (mitogenome) has been proposed as a powerful molecular marker for phylogenetic reconstruction, evolutionary genomics, and population‐level studies due to its maternal inheritance, relatively conserved structure, and elevated evolutionary rates. Anurans (frogs and toads), comprising 7693 species and ~90% of extant amphibian diversity, exhibit extensive mitogenome variability, yet the evolutionary significance of this structural diversity remains poorly understood. Using 277 anuran mitogenomes (spanning 35 families) and outgroup comparisons, phylogenomic reconstructions and gene order evolution analyses were conducted. Phylogenetic analyses resolved five major clades: Leiopelmatoidea (basal lineage), Discoglossoidea, Pipoidea, Pelobatoidea, and the crown group Neobatrachia. Archaeobatrachians emerged as a paraphyletic group, with sequential divergences of the first four clades. Neobatrachia was monophyletic, with Heleophrynidae and Sooglossidae occupying basal positions. Subsequent diversification revealed two major neobatrachian subclades: one uniting Calyptocephalellidae with Myobatrachoidea and Hyloidea, and another comprising Ranoidea, a topology consistent with recent phylogenomic studies but highlighting persistent conflicts within Hyloidea and Ranoidea. Divergence time estimation traced the origin of Anura to the Early‐Late Triassic boundary, with major neobatrachian radiations occurring from the Late Cretaceous through the Early Neogene. CREx‐based comparative analyses identified 58 distinct gene arrangement patterns, including lineage‐specific synapomorphic rearrangements that were phylogenetically mapped. Our study provides a robust mitogenomic framework for Anura, reconciling deep phylogenetic discordance while revealing novel patterns of gene order evolution. These findings establish critical foundations for future investigation into the mechanisms driving mitogenomic structure diversification and its interplay with the evolutionary success of this ecologically vital vertebrate group.

## Graphical abstract

By analyzing 277 anuran mitochondrial genomes, this study reconstructs a robust phylogeny for frogs and toads, tracing their origins to the Triassic and major radiations to the Cretaceous‐Neogene. The research identifies 58 distinct gene arrangement patterns, providing a novel evolutionary framework. These findings resolve deep phylogenetic relationships and establish a foundation for understanding mitogenome evolution in anurans.

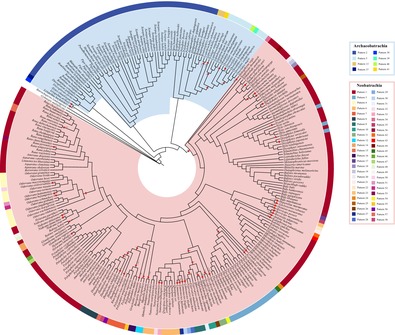

## 1 Introduction

Anura (frogs and toads), with currently 7693 described species (Frost 2024), dominate amphibian diversity and make up 90% of amphibian species. Their evolutionary trajectory, marked by the iconic water‐to‐land transition and subsequent radiation into diverse ecological niches, positions them as a model system for addressing questions in evolutionary biology, biogeography, and ecology (Fei et al. 2009). Consequently, resolving their phylogenetic relationships has become a cornerstone for comparative studies (Zhang et al. 2013; Feng et al. 2017; Portik, Streicher, Blackburn, et al. 2023). Advances in phylogenomic approaches have substantially clarified deep anuran divergences and diversification patterns (e.g., Zhang et al. 2013; Feng et al. 2017; Hime et al. 2021; Portik, Streicher, Blackburn, et al. 2023, Portik, Streicher, and Wiens 2023). Nevertheless, persistent conflicts plague relationships within several ecologically pivotal lineages, including Hylidae, Bufonidae, Leptodactylidae, and Telmatobiidae (Jetz and Pyron 2018; Hime et al. 2021; Portik, Streicher, Blackburn, et al. 2023, Portik, Streicher, and Wiens 2023), underscoring the need for integrative analyses to reconcile topological discordance.

Mitochondrial genome (mitogenome) has been proposed as a powerful molecular marker for phylogenetic reconstruction, evolutionary genomics, and population‐level studies due to its maternal inheritance, relatively conserved structure, and elevated evolutionary rates (Du et al. 2019; Chen, Qian, et al. 2022; Wang et al. 2023). Their utility in resolving relationships across taxonomic scales, from deep divergences to recent radiations, has been well demonstrated in vertebrates (Nicolas et al. 2020; Zhang et al. 2013; Chen et al. 2017; Zhu et al. 2023). The typical vertebrate mitogenome comprises a circular DNA molecule encoding 13 protein coding genes (PCGs), 22 transfer RNAs (tRNAs), two ribosomal RNAs (rRNAs), and two noncoding regions: the L‐strand replication origin (OL) and the control region (CR) containing transcriptional regulatory elements (Boore 1999; Montaña‐Lozano et al. 2022). While sequence variation provides primary phylogenetic signal, gene order rearrangements offer complementary evolutionary evidence, particularly for resolving deep nodes (Chen et al. 2011; Tan et al. 2018; Sun et al. 2022). Most vertebrate retain the ancestral gene arrangement (“vertebrate‐typical”), characterized by conserved positioning of all 37 genes and CR across lineages from teleost fishes to eutherian mammals (Montaña‐Lozano et al. 2022). Within Anura, archaeobatrachians predominantly exhibit this plesiomorphic organization (Mauro et al. 2004), though exceptions occur in *Leiopelma archeyi* (Irisarri et al. 2010) and *Oreolalax* species (Luo et al. 2022). In contrast, neobatrachians universally share a derived “neobatrachian‐typical” rearrangement involving tRNA and CR repositioning (Xia et al. 2014). Subclade‐specific synapomorphic rearrangements further characterize lineages like Natatanura (Chen, Qian, et al. 2022; Cui et al. 2022) and Afrobatrachia (Kurabayashi and Sumida 2013), providing critical topological support in contested phylogenies (e.g., Chen et al. 2017). Despite these advances, current understanding remains fragmented due to lineage‐focused studies, leaving unresolved the macroevolutionary patterns of mitogenomic architecture across Anura and the mechanistic drivers of these structural innovations.

Leveraging the most comprehensive mitogenomic dataset for Anura to date (*n* = 277 complete mitogenomes; current through April 2024), we conducted a tripartite investigation integrating structural, phylogenetic, and evolutionary analyses in this study. First, we quantified fundamental mitogenomic structure through compositional statistics, including genome size, GC/AT‐skew dynamics, nucleotide bias, and codon usage patterns. Second, we reconstructed deep anuran phylogeny using partitioned maximum likelihood and Bayesian analyses across four complementary datasets, that is, the concatenated nucleotide sequences of tRNAs, rRNAs, and PCGs (with or without third codon positions); the concatenated nucleotide sequences of PCGs; the concatenated amino acid sequences of PCGs. These phylogenomic frameworks were subsequently employed in Bayesian dating analyses incorporating 13 fossil calibrations to temporalize major cladogenetic events. Furthermore, we implemented a phylogenetically informed comparative genomics approach to systematically catalog mitochondrial gene rearrangements across all sampled lineages. This included ancestral state reconstruction of rearrangement events, mechanistic modeling of potential evolutionary drivers (e.g., tandem duplication‐random loss), and assessment of their phylogenetic congruence through reconciliation analyses. By integrating these multidimensional datasets, our study establishes the holistic framework for understanding mitogenomic evolution in Anura while resolving persistent uncertainties in their deep phylogenetic relationships.

## 2 Material and Methods

### 2.1 Source of Data

We compiled a mitogenomic dataset encompassing 277 anuran species through systematic retrieval of all available complete mitochondrial genomes from NCBI GenBank (last accessed on April 21, 2024; https://www.ncbi.nlm.nih.gov/search/). This curated dataset spans 35 families under the current anuran classification framework (Frost 2024), representing > 60% of recognized family diversity. The final alignment matrix was used to time stamp phylogenetic and structural genomic analyses. Taxon sampling details, including taxonomic hierarchy and GenBank accession numbers, are comprehensively documented in Table S1. To ensure analytical rigor, we implemented a phylogenetically informed subsampling strategy that maximized taxonomic coverage while minimizing long‐branch attraction artifacts through outlier sequence removal.

### 2.2 Mitogenomic Characterization

After obtaining GenBank accession numbers for 277 anuran species, complete mitochondrial genomes were systematically retrieved and subjected to standardized preprocessing. Gene annotation was performed using PhyloSuite v1.2.3 (Zhang et al. 2020; Xiang et al. 2023) with the following modular workflow: (1) importation of mitochondrial genome data from NCBI in GenBank format; (2) parameter configuration specifying sequence type as mitogenome and employing the vertebrate mitochondrial genetic code; (3) systematic normalization of gene nomenclature to ensure consistency for ortholog identification; (4) extraction of all 13 PCGs, 22 tRNAs, and two rRNAs through batch processing. Compositional analyses and relative synonymous codon usage (RSCU) values were conducted in MEGA 7.0 (Kumar et al. 2016) under the vertebrate mitochondrial genetic code (genetic code = 2). We quantified nucleotide usage asymmetries through AT‐skew [(A‐T)/(A + T)] and GC‐skew [(G‐C)/(G + C)] indices (Perna and Kocher 1995).

### 2.3 Phylogenetic Analysis

Phylogenetic reconstruction was conducted across 277 anuran species, with two species from Caudata and Gymnophiona used as outgroups, with analyses restricted to 11 protein‐coding genes, 11 tRNAs and two rRNAs due to incomplete mitochondrial genomes (missing *atp8*, *nad5*, and some tRNAs in certain species). Gene sequences were aligned in MEGA 7.0 with ClustalW (Kumar et al. 2016), where amino acid alignments of PCGs (translated using vertebrate mitochondrial code) guided nucleotide sequence alignment. Four datasets were generated: (1) 24NT dataset (13,012 bp) with 11 PCGs (including third codon positions), 11 tRNAs, and two rRNAs; (2) 24NTS dataset (9862 bp) with 11 PCGs (excluding third codon positions), 11 tRNAs, and two rRNAs; (3) 11NT dataset (9450 bp) for 11 PCGs nucleotides; and (4) 11AA dataset (3150 aa) for 11 PCGs amino acids. The maximum likelihood (ML) and Bayesian inference (BI) methods were used for phylogenetic analysis based on the four datasets. Before starting the actual phylogenetic analysis, ModelFinder 2.2.0 (Kalyaanamoorthy et al. 2017) was used to determine the best‐fit partitioning schemes and nucleotide substitution models (Table S2), with model selection based on the Akaike information criterion (AIC) for ML analyses and the Bayesian information criterion (BIC) for BI analyses. ML analysis was performed using IQ‐TREE (Minh et al. 2013; Nguyen et al. 2015) with the optimal partition strategy and models, incorporating 5000 ultrafast bootstrap replicates. BI analysis was conducted by MrBayes 3.2.7 (Ronquist et al. 2012), with four Markov Chain Monte Carlo (MCMC) chains running for 10,000,000 generations, sampling every 1000 generations. Two independent runs were performed to confirm the convergence of the posterior parameter distributions. The initial 25% of trees were discarded as burn‐in, and then Bayesian posterior probabilities were obtained as 50% majority rule from trees sampled at stationarity.

### 2.4 Divergence Time Analyses

Divergence time was estimated using MCMCTree in PAML 4.10 (Yang 2007), with the ML topology from the 24NT dataset serving as the reference tree. Thirteen fossil calibration points (Table 1), adopted from Feng et al. (2017) and Portik, Streicher, and Wiens (2023), were applied to constrain deep amphibian nodes, ensuring 95% prior probability mass fell within soft bounds (2.5% density extending beyond bounds). The root age (Gymnophiona–Caudata divergence) was fixed at 320 million years ago (Mya) (95% CI: 308.0–325.6 Mya) based on TimeTree (Kumar et al. 2022). Substitution rates were initially estimated using BASEML (GTR model), and a strict molecular clock was implemented with a gamma prior for the overall rate (rgene_gamma: G (1, 3.693, 1)). The independent rate model (clock = 2) defined rate variation across branches. The MCMC run was fist executed for 1000,000 generations as burn‐in, then sampled every 1000 generations until it gathered 10,000 samples. Two MCMC runs with random seeds were compared for convergence. We used print = 1 in MCMCTree to set the output of merging and summarizing these analyses to calculate the posterior mean divergence times and 95% highest posterior density (HPD) credible intervals on divergence times. Effective sample size (ESS) values of parameters were calculated for the combined results in Tracer 1.7.2 to evaluate the convergence of these results (ESS > 200) (Rambaut et al. 2018). Final divergence times, including posterior means and 95% HPD intervals, were visualized using FigTree 1.4.3.

**TABLE 1:** Fossil calibrations used for divergence time analyses.

Num	Node	Fossils	Minimum	Maximum	Source
1	Batrachia	*Triadobatrachus massinoti*	252	272.8	Cannatella (2015); Benton et al. (2015)
2	Discoglossoidea	*Lberobatrachus angelae*	125	252	Gómez and Turazzini (2016)
3	Alytidae	*Latonia*	23	252	Roček and Rage (2000); Yuan et al. (2000)
4	Pipoidea	*Rhadinosteus parvus*	148.1	252	Cannatella (2015); Báez (2013)
5	Pipidae	*Pachycentrata taqueti*	83.6	148.1	Cannatella (2015)
6	Scaphiopodidae	*Scaphiopus guthriei*	50.3	148.1	Gao and Chen (2017)
7	Pelobatidae+Megophryidae	*Gobiates spinari*	86.3	148.1	Chen et al. (2016); Roček (2008)
8	Calyptocephalellidae	*Calyptocephalella*	61.7	148.1	Báez (2000)
9	Myobatrachoidea	*Lechriodus*	54.6	148.1	Sanchiz (1998); Evans et al. (2008)
10	Hylinae	*Acris barbouri*	16.3	148.1	Holman (2003); Marjanovic and Laurin (2007)
11	Eleutherodactylidae+Brachycephalidae	*Eleutherodactylus*	29.5	148.1	Blackburn et al. (2020)
12	Bufonidae	*Bufo*	56	148.1	Báez (2000); Báez and Nicoli (2004); Walker et al. (2018)
13	Pyxicephalidae+Ptychadenidae	*Thaumastosaurus*	39.5	148.1	Lemierre et al. (2021)

### 2.5 Mitogenomic Gene Rearrangement Analysis

Mitochondrial gene orders for all 277 species were curated from GenBank and re‐annotated using MITOS2 (Bernt et al. 2013; Al‐Arab et al. 2017; Donath et al. 2019), with manual verification to ensure accuracy. All mitochondrial components (PCGs, tRNA, rRNAs, and CR) were incorporated into a standardized text file for comparative analysis. Species sharing identical gene orders were grouped, and pairwise comparisons of arrangements were conducted using the Common interval Rearrangement Explorer2 (CREx2; Bernt et al. 2007; Hartmann et al. 2019) and Tree Rearrangement Explorer (TreeREx; Bernt et al. 2008) to infer rearrangement mechanisms and evolutionary pathways. For complex cases (e.g., duplications and incomplete gene sets) incompatible with the automated workflow of CREx2 and TreeREx, rearrangement events were manually resolved by comparing with related species.

## 3 Results

### 3.1 Mitogenome Composition

The mitogenomes of the examined anuran species varied in size from 15,415 bp ( *Dryophytes femoralis* ) to 28,757 bp ( *Breviceps adspersus* ) (Table S3). Most species contained the standard 37 mitochondrial genes, including 13 PCGs, 22 tRNAs, and two rRNAs, with exceptions detailed in Table S4. Nucleotide composition was strongly AT‐biased, ranging from 52.8% ( *Hoplobatrachus chinensis* ) to 68.5% ( *Hyperolius marmoratus* ) (Table S3). Negative AT‐skews (T > A) dominated across species, while GC‐skews were primarily negative (C > G), except in Myobatrachidae species ( *Taudactylus pleione* ), two Ranidae species ( *Odorrana grahami* and *O. macrotympana* ), and one Ranixalidae species (*Indirana semipalmdata*), which exhibited positive GC‐skews (G > C). Full compositional metrics are summarized in Table S3.

**FIGURE 1:**
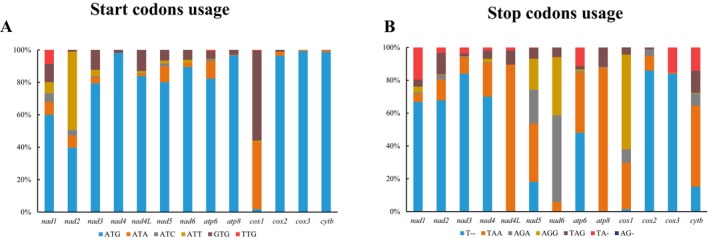
Usage of start (A) and stop (B) codons in the 13 mitochondrial protein‐coding genes of 277 anurans. Detailed information was presented in Tables S5 and S6.

The codon usage analysis of 13 PCGs across 277 anuran species revealed conserved patterns in initiation and termination codons (Figure 1, Tables S5 and S6). Initiation codons were predominantly ATG, used by 11 genes: *atp8*, *atp6*, *cox2*, *cox3*, *cytb*, *nad1*, *nad3*, *nad4*, *nad4L*, *nad5*, and *nad6*. Exceptions included *nad2*, which favored ATT, and *cox1*, which primarily utilized GTG. Overall, six distinct start codon types were identified (Figure 1A, Table S5). Termination codons included four complete types (AGA, AGG, TAA, and TAG) and three incomplete types (AG‐, T‐, and TA‐), with the incomplete T‐ (single thymine) being the most common, dominating in seven genes (*atp6*, *cox2*, *cox3*, *nad1*, *nad2*, *nad3*, and *nad4*) (Figure 1B, Table S6). Complete stop codons were prevalent in specific genes: TAA in *nad4L*, *nad5*, *atp8*, and *cytb*; AGA/AGG in *nad6* and *cox1* (Figure 1B, Table S6). When mapped onto the phylogenetic framework, the predominant start and stop codons of each gene were broadly conserved across major anuran clades (Figures S1 and S2). Alternative codon variants were distributed among phylogenetically distant taxa rather than confined to specific lineages. No consistent deep‐level clade‐specific patterns were detected.

**FIGURE 2:**
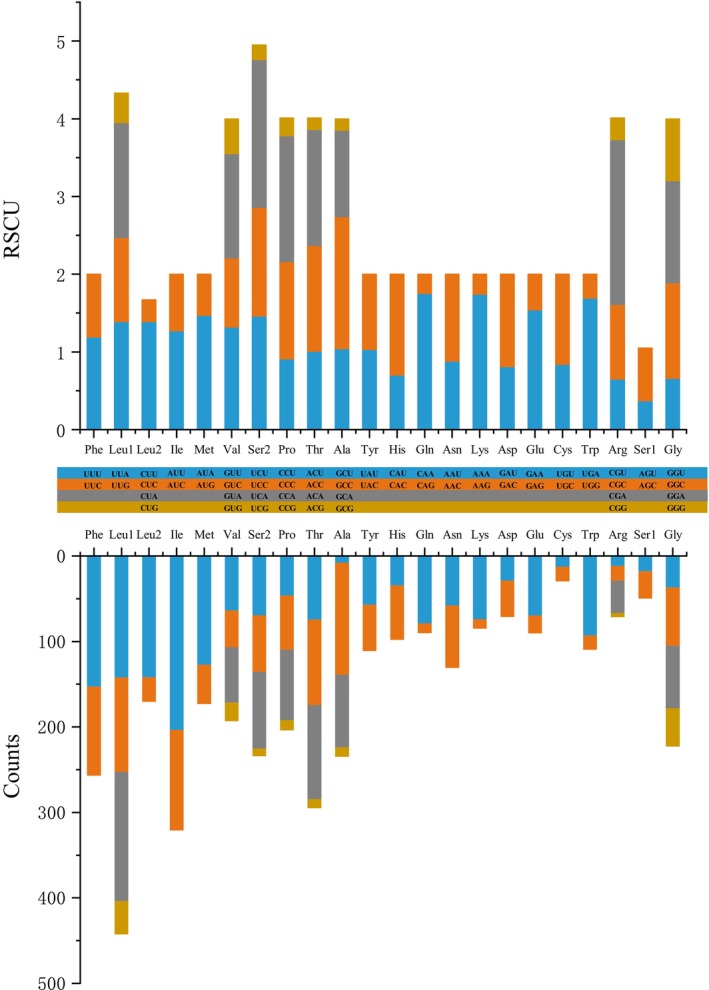
Usage of amino acid coding codon in the mitogenome of Anura species. The different colors in the column chart represent the codon families corresponding to the amino acids below, with the same colors representing the same codon families. The stop codon is excluded. Detailed information is presented in Table S7.

Relative synonymous codon usage (RSCU) analysis of the 13 PCGs across 277 anuran species (Figure 2, Table S7) revealed distinct codon preferences after excluding stop codons, with 3759 amino acids analyzed. Leucine (Leu, 613.2 occurrences) and serine (Ser, 284.2) utilized six synonymous codons each, while the remaining amino acids were encoded by either two or four. High‐abundance residues included Leu, isoleucine (Ile, 321), alanine (Ala, 307), threonine (Thr, 295.2), Ser, and phenylalanine (Phe, 256.9), contrasting with low frequencies of glutamic acid (Glu, 90.5), glutamine (Gln, 90.1), lysine (Lys, 85), arginine (Arg, 71.7), aspartic acid (Asp, 71.2), and cysteine (Cys, 29.7). Codon‐specific trends highlighted Leu (CUA/CUU/UUA), Ile (AUU), Ala (GCC), and Phe (UUU) as the most frequent, whereas Ala (GCG), Gln (CAG), Lys (AAG), Thr (ACG), Ser (UCG), and Arg (CGG) were least utilized. RSCU values further identified CGA (Arg), UCA (Ser), CAA (Gln), AAA (Lys), GCC (Ala), and UGA (Trp) as dominant codons, while AAG (Lys), CAG (Gln), CCG (Pro), UCG (Ser), ACG (Thr), and GCG (Ala) were rare, reflecting strong AT‐bias.

### 3.2 Phylogenetic Relationships Among Anura

**FIGURE 3:**
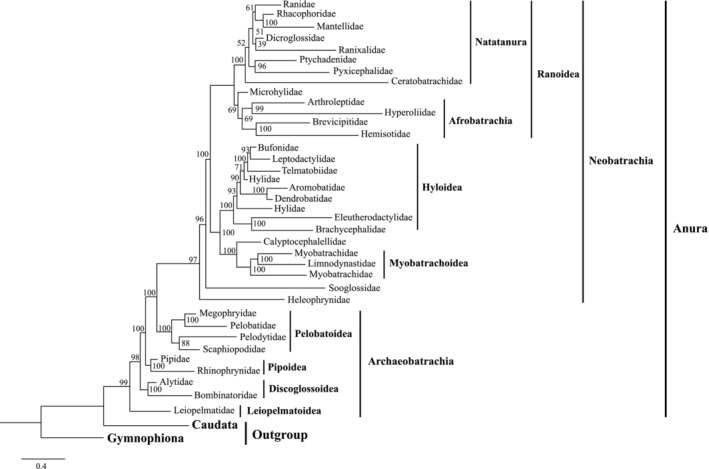
Maximum likelihood phylogeny based on 24NT dataset. For visual clarity, species within each anuran family have been pruned and collapsed into a single tip labeled with the family name. The original tree topology, branch lengths, and nodal support values are preserved. Family‐level taxonomy follows AmphibiaWeb (2024).

Phylogenetic relationships among 277 anuran species (35 families) and two outgroups (Gymnophiona and Caudata) were reconstructed using Bayesian Inference (BI) and Maximum Likelihood (ML) methods across four datasets (i.e., 24NT, 24NTS, 11NT, and 11AA datasets; see Figures 3 and S3–, S17). Anura were robustly recovered as a monophyletic group, with five major clades consistently recovered: Leiopelmatoidea, Discoglossoidea, Pipoidea, Pelobatoidea, and Neobatrachia. Archaeobatrachians were resolved as successively diverging lineages, each exhibiting well‐supported family‐level monophyly (Figures 3 and S3–, S9). Within the dominant Neobatrachia clade, the deepest splits involved Heleophrynidae and Sooglossidae, followed by the divergence of two strongly supported lineages: one comprising Calyptocephalellidae, Myobatrachoidea, and Hyloidea, and the other corresponding to Ranoidea (Figures 3 and S3–, S9). However, several higher‐level relationships showed significant incongruence across different dataset and analytical methods. These included the relative branching order of Discoglossoidea and Pipoidea, internal relationships within Pelobatoidea, the phylogenetic position of Microhylidae relative to Afrobatrachia and Natatanura, and the detailed topology among the major Asian families of Natatanura (Figures 3 and S3–, S9). Comprehensive presentations of the phylogenetic trees, including detailed topological structures and node support values are presented in Supporting Information File 1.

### 3.3 Divergence Time Estimations

The estimated divergence times for the nodes of the phylogeny (Figure S18) were summarized in Table S8 and traced the evolution of major anuran lineages from the Permian to the Neogene. The origin of Anura dated to the Permian (262.64 Mya; 95% HPD: 252.16–272.78 Mya), with the earliest divergence between Leiopelmatidae and other anurans occurring in the Triassic (221.09 Mya; 202–239.28 Mya). Neobatrachia split from Peobatoidea (Scaphiopodidae, Pelobatidae, Pelodytidae, and Megophryidae) during the Late Triassic to Middle Jurassic (171.92–203.49 Mya), followed by successive splits within Neobatrachia: Heleophrynidae diverged in the Early to Late Jurassic (155.34–183.53 Mya), Sooglossidae in the Middle to Late Jurassic (150.42–177.32 Mya), and the Calyptocephalellidae + Myobatrachoidea + Hyloidea clade from Ranoidea in the Jurassic (145.64–171.31 Mya). The separation between Calyptocephalellidae + Myobatrachoidea and Hyloidea occurred later (Late Jurassic to Early Cretaceous: 130.27–152.73 Mya), with the crown‐group Hyloidea originating in the Cretaceous (126.31 Mya; 113.64–137.99 Mya). Within Ranoidea, the basal divergence between Afrobatrachia + Microhylidae and Natatanura dated to the Late Jurassic (approximately 145.51 Mya; 133.14–157.32 Mya), while Afrobatrachia diversified in the Late Jurassic to Middle Cretaceous (116.71–143.12 Mya). Microhylidae radiated in the Early Cretaceous (118.94 Mya; 103.8–134.41 Mya), and Natatanura lineages emerged in the Late Jurassic to Early Cretaceous (125.35–148.53 Mya). Major genus‐level radiations within Neobatrachia occurred from the Late Cretaceous to the Early Neogene (Figure S18 and Table S8).

### 3.4 Mitogenome Gene Order Patterns in Anura

**FIGURE 4:**
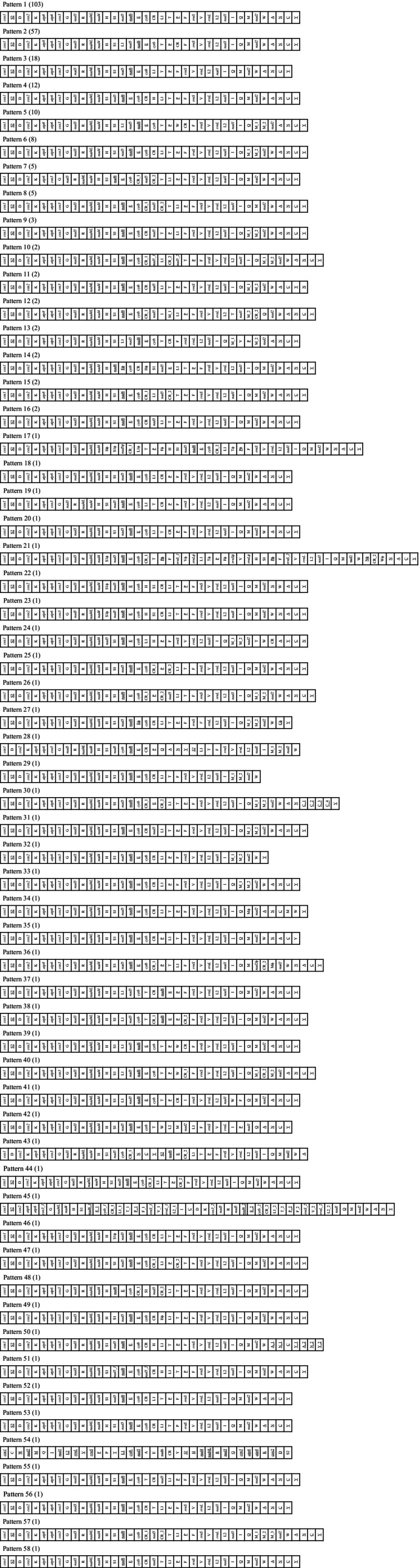
Patterns of mitogenome arrangement in anurans, which were labeled as Pattern 1 ~ Pattern 58 in descending order of the frequency of rearrangement types. The number of included species of each pattern was shown in parentheses. Underlined genes were encoded on the light strand. ϕ indicated a pseudogene. The tRNA genes were shown using single upper‐case letter amino acid codes. L1, L2, S1, and S2 indicated tRNAs for Leu(CUN), Leu(UUR), Ser(AGY), and Ser(UCN), respectively. Other gene abbreviations were: *rrnS* and *rrnL*, 12S and 16S ribosomal RNAs; *cox1‐3*, cytochrome c oxidase subunits 1–3; *cytb*, cytochrome b; *nad1‐6* and *4 L*, NADH dehydrogenase 1–6 and 4 L.

The mitochondrial genome arrangements were systematically analyzed in 277 anuran species, which revealed 58 distinct gene order patterns (Figure 4). Among these, 57 species retained the ancestral archaeobatrachian arrangement (typical of vertebrates), while the remaining 220 species exhibited 57 novel arrangement types, including 103 conforming to the neobatrachian archetype. The patterns were ranked (Pattern 1–58) by rearrangement frequency, with the neobatrachian arrangement (Pattern 1) being the most dominant (Table 2). Comparative analysis against ancestral (archaeobatrachian) and derived (neobatrachian) templates, together with CREx‐based rearrangement inference (Figures S19 and S20), identified lineage‐specific structural innovations, highlighting dynamic mitogenome evolution in Anura.

**TABLE 2:** Mitochondrial genome patterns contained in the two suborders of Anura, respectively.

Suborder	Taxa	Mitochondrial genome pattern
Archaeobatrachia	Leptodactylidae	Pattern 2
	Alytidae	Pattern 2
	Bombinatoridae	Pattern 2
	Rhinophrynidae	Pattern 2
	Pipidae	Pattern 2
	Scaphiopodidae	Pattern 2
	Pelobatidae	Pattern 2
	Pelodytidae	Pattern 2
	Megophryidae	Pattern 2, 5, 13, 37, 38, 39, 40, 41
Neobatrachia	Heleophrynidae	Pattern 34
	Sooglossidae	Pattern 1
	Myobatrachidae	Pattern 1, 44
	Limnodynastidae	Pattern 1
	Calyptocephalellidae	Pattern 1
	Brachycephalidae	Pattern 18, 19, 20
	Eleutherodactylidae	Pattern 1
	Dendrobatidae	Pattern 1, 25
	Aromobatidae	Pattern 1
	Hylidae	Pattern 1, 3
	Telmatobiidae	Pattern 1
	Leiopelmatidae	Pattern 1
	Bufonidae	Pattern 1
	Hemisotidae	Pattern 35
	Brevicipitidae	Pattern 21, 22, 23
	Hyperoliidae	Pattern 36
	Arthroleptidae	Pattern 17
	Microhylidae	Pattern 1, 42, 43
	Ceratobatrachidae	Pattern 24
	Pyxicephalidae	Pattern 45
	Ptychadenidae	Pattern 3
	Ranixalidae	Pattern 54
	Dicroglossidae	Pattern 6, 9, 10, 11, 26, 27, 28, 29, 30, 31, 32, 33
	Mantellidae	Pattern 12
	Rhacophoridae	Pattern 7, 8, 16, 56, 57, 58
	Ranidae	Pattern 1, 4, 14, 15, 46, 47, 48, 49, 50, 51, 52, 53

**FIGURE 5:**
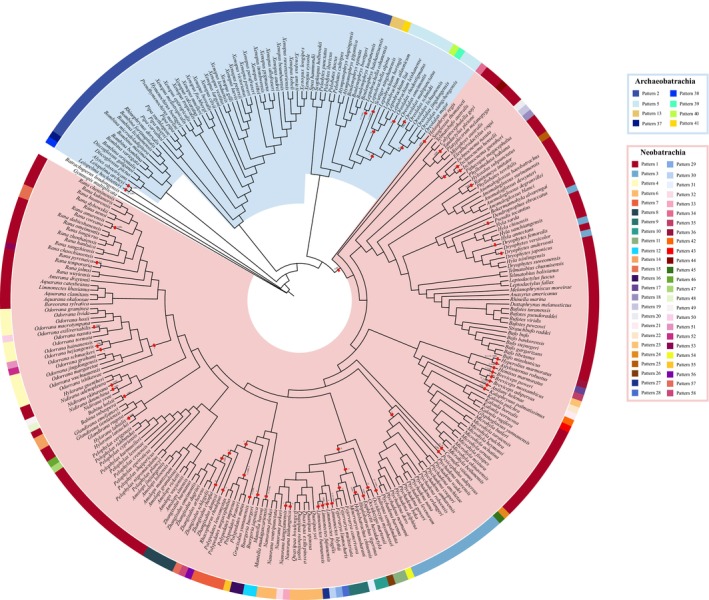
Maximum‐likelihood phylogeny of Anura based on 24NT dataset, with clades colored by suborder (Archaeobatrachia: Blue; Neobatrachia: Pink). The outer ring in the phylogenetic tree represents the distribution of mitochondrial gene orders (MGO), with 58 patterns identified across 277 species and labeled for each suborder in the panels to the right. MGOs that deviated from the ground pattern (Pattern 2 for Archaeobatrachia and Pattern 1 for Neobatrachia) were explained by a series of CREx‐predicted gene rearrangement events: Transposition (T), inversion (I), inverserve transposition (IT), and tandem duplication‐random loss (TDRL). Gene tandem duplication (D) and loss (L) events were also indicated.

A total of eight mitochondrial gene order patterns (Patterns 2, 5, 13, 37–41) were identified in archaeobatrachians (Figures 4, 5 and S19, Table 2). Pattern 2, representing the ancestral vertebrate arrangement (Figures 4 and 5), was predominant in most species, while deviations were limited to Megophryidae (six patterns) and Leiopelmatidae (two patterns). Neobatrachians displayed 50 gene order patterns (Figures 4 and 5, Table 2), among which Pattern 1 (the typical neobatrachian arrangement) was found in nearly half of the neobatrachian species (Figure 5). This pattern originated from tandem duplication‐random loss (TDRL) events affecting the *trnL1*‐*nad5*‐*nad6*‐*trnE*‐*cytb*‐*trnT*‐*trnP*‐CR region, leading to the formation of the derived *LTPF* tRNA cluster (Figures 5 and S20). Exceptions included Heleophrynidae ( *Heleophryne regis* , Pattern 34 via TDRL) and *Taudactylus pleione* (Myobatrachidae, Pattern 44 via TDRL). Within Hyloidea, Pattern 1 was dominant in Eleutherodactylidae, Phyllomedusinae (Hylidae), most Dendrobatidae (excluding *Ranitomeya imitator* ), Aromobatidae, and Hylinae (Hylidae, excluding *Pseudis tocantins* , *Dryophytes femoralis* , and *Dendropsophus ebraccatus* ), with five divergent patterns observed in the remaining species (Figure 5, Table 2). Regarding the Ranoidea species, a high degree of diversity was observed, with 42 patterns across 150 species (Figure 5, Table 2). Afrobatrachia and Microhylidae exhibited six and two types, respectively, while the remaining 34 patterns were found in Natatanura species, indicating an unexpectedly high diversity in mitochondrial genome organization (Figure 5, Table 2).

### 3.5 Gene Rearrangements in Anuran Mitogenomes

Our study identified four types of mitochondrial genome rearrangement in Anurans, that is, inversions (reversed gene orientation), transpositions (position shifts without inversion), inverse transpositions (position shifts with inversion), and tandem duplication‐random loss (TDRL) events. We found that tRNA genes had higher rearrangement rates compared to protein‐coding or rRNA genes. The rearrangement events formed two distinct patterns: Lineage‐specific changes restricted to closely related species and rare, homoplastic events shared across distantly related taxa or found in single species, suggesting both phylogenetic constraint and stochastic evolutionary processes.

Within Archaebatrachia, mitochondrial genome rearrangements revealed both shared and lineage‐specific patterns. *Leptobrachium* and *Oreolalax* species (Megophryidae) shared a transposition of *trnW* (Pattern 5, 39, 40; Figures 5 and S19), along with tandem duplications of *trnM* in all *Leptobrachium* and most *Oreolalax* species ( *O. lichuanensis* and *O. schmidti* excluded; Pattern 5, 39, 40; Figures 5 and S19). In contrast, *Leptobrachella* species showed a distinct nontandem duplication of *trnM*, coupled with transpositions of *trnP* and *trnV* and a loss of *trnW* (Pattern 13; Figures 5 and S19). Nontandem duplications of CR were observed in *Leiopelma hochstetteri* and *O. schmidti* , but with divergent insertion sites: The additional CR in *L. hochstetteri* was located upstream of *nad6*, while in *O. schmidti* , the CR was inserted downstream of *trnM* (Pattern 38, 40; Figures 5 and S19).

Mitochondrial genome rearrangements in Neobatrachia showed extensive diversity, driven by tandem duplications, transpositions, inversions, and pseudogenization (Figures 5 and S20). Tandem *trnM* duplications (forming the *IQMM* tRNA cluster) dominated Dicroglossidae and Ceratobatrachidae (Pattern 6, 9–11, 24, 26–33; Figures 5 and S20), while *Zhangixalus chenfui* (Rhacophoridae) uniquely possessed three *trnM* copies (Pattern 57; Figures 5 and S20). Non‐tandem *trnM* duplications occurred in Mantellidae, *Heleophryne regis* , and *Hyperolius marmoratus* , with additional pseudogenized *trnM* in *Heleophryne regis* and *Hyperolius marmoratus* (Pattern 12, 34, 36; Figures 5 and S20). Transposition events included *trnH* repositioning downstream of CR in most *Odorrana* species (Pattern 4, 50, 51; absent in *O. schmackeri* , Pattern 52; Figures 5 and S20), *trnL1* relocation downstream of *trnT* in *Polypedates*, *Zhangixalus*, and *Rhacophorus* (Pattern 7, 8, 57, 58; Figures 5 and S20), and *nad5* translocation downstream of CR in Mantellidae, Rhacophoridae (excluding *Gracixalus yunnanensis* and *Rhacophorus rhodopus* ), and Dicroglossidae (including *Fejervarya*, *Occidozyga*, *Hoplobatrachus*, *Minervarya* and *Euphlyctis hexadactyla* ) (Pattern 7–12, 16, 26, 31, 56–58; Figures 5 and S20). CR dynamics involved loss in *Dryophytes andersonii* , *D. femoralis* , *Pseudis Tocantins* , 15 *Ptychadena* species, and *Anilany helenae* (Pattern 3, 42; Figures 5 and S20) and repositioning downstream of *trnT* in *G. yunnanensis* and three *Ischnocnema* species (Pattern 18–20, 55; Figures 5 and S20). Lineage‐specific transpositions included *trnL1* downstream of *trnP* in *Fejervarya* (Pattern 9; Figures 5 and S20), *trnN* downstream of *trnY* in *Occidozyga* and divergent *trnT* relocations in Mantellidae (downstream of *trnL2*) (Pattern 11, 12; Figures 5 and S20). Afrobatrachia showed *trnA* transposition downstream of *trnN*, except in *Breviceps mossambicus* (*trnN* upstream of *trnW*) (Pattern 17, 21–23, 35, 36; Figures 5 and S20), while *Occidozyga* species shared *trnN* transposition downstream of *trnY* (Pattern 11; Figures 5 and S20). Other transposition events were unique to single species (Figures 5 and S20), such as CR transposition in *Breviceps adspersus* (Pattern 21), multi‐gene transpositions in *Cornufer vitianus* (transposition of *trnH*, *trnS1*, CR, *trnT*, *trnY, trnC*; Pattern 24), etc. Inversion events included *trnP*/*trnT* in *Hyperolius marmoratus* (Pattern 36), *trnP* in *Rana pyrenaica* (Pattern 53), and genome‐wide inversions in *I. semipalmdata* (Pattern 54; Figures 5 and S20). Inverse transposition events only happened in *Limnonectes blythii* (*trnC*; Pattern 28) and *Hyperolius marmoratus* (*trnT*‐*trnP*; Pattern 36). In addition, gene loss was also identified in a subset of species (Table S4). Notably, the *atp8* gene was absent in all examined *Polypedates* species and *Ischnocnema henselii* (Patterns 7, 19; Figures 5 and S20, Table S4). A total of 28 TDRL events contributed to nontandem duplications and structural diversity, highlighting the dynamic interplay of lineage‐specific adaptation and stochastic genomic plasticity in Neobatrachia.

## 4 Discussion

### 4.1 Phylogenetic Relationships Among Anura

In this study, phylogenetic relationships within Anura were reconstructed using Bayesian inference (BI) and maximum likelihood (ML) methods. Our analysis utilized four datasets derived from published mitochondrial genomes: (1) 24NT dataset (13,012 bp), comprising 11 PCGs (including third codon positions), 11 tRNAs, and two rRNAs; (2) 24NTS dataset (9862 bp) with 11 PCGs (excluding third codon positions), 11 tRNAs, and two rRNAs; (3) 11NT dataset (9450 bp), covering nucleotides for 11 PCGs; and (4) 11AA dataset (3150 aa), based on the amino acids of 11 PCGs. The resulting topologies inferred from these datasets were largely consistent, although minor conflicts in node support for branching order were observed. Our findings are in agreement with recent large‐scale phylogenies (e.g., Pyron and Wiens 2011; Zhang et al. 2013; Feng et al. 2017; Jetz and Pyron 2018; Hime et al. 2021; Zhang et al. 2021; Portik, Streicher, Blackburn, et al. 2023, Portik, Streicher, and Wiens 2023), thereby reinforcing the consensus on deep anuran relationships while also shedding light on specific clades within Neobatrachia where discordances remain unresolved. Detailed comparisons of these relationships with other researches are provided in the Supporting Information File 1.

### 4.2 Divergence Time Estimation

Estimates of divergence times for key anuran evolutionary nodes show both consistency and discrepancies across studies. The origin of crown Anura was dated to the Late Permian (~262.64 Mya; 95% HPD: 252.16–272.78 Mya), overlapping with the estimates from Feng et al. (2017; 266.8–274.7 Mya) and Hime et al. (2021; 268.8–275.3 Mya), but is younger than those from Zhang et al. (2013; 280 Mya) and Jetz and Pyron (2018; 286.90 Mya). The basal anuran split (Leiopelmatoidea vs. other lineages) was estimated at ~221.09 Mya (202–239.28 Mya), which is consistent with Jetz and Pyron (2018; 222.11 Mya) but older than the estimates from Feng et al. (2017; 210 Mya), Hime et al. (2021; 213.9 Mya), and Portik, Streicher, and Wiens (2023; 179.34 Mya). Notably, Zhang et al. (2013) proposed an even older divergence (~243.7 Mya) for this node. The origin of crown Neobatrachia, defined by the Pelobatoidea–Neobatrachia split, was estimated at around 187.24 Mya (171.92–203.49 Mya). This estimate falls between that of Zhang et al. (2013; 206.5 Mya) and younger estimates from studies such as Feng et al. (2017; 177.6 Mya) and Portik, Streicher, and Wiens (2023; 159.69 Mya). Most studies agree that major neobatrachian divergences occurred during the Jurassic period (Zhang et al. 2013; Feng et al. 2017; Hime et al. 2021; Jetz and Pyron 2018), contrasting with Portik, Streicher, and Wiens (2023), who inferred a Cretaceous crown age. These discrepancies likely reflect differences in fossil calibrations, molecular clock models, and taxon sampling across studies.

Our divergence time estimates for Neobatrachia are close to those of Zhang et al. (2013) and Jetz and Pyron (2018), but older than the proposals from Hime et al. (2021), Feng et al. (2017), and Portik, Streicher, and Wiens (2023). The Cretaceous‐Paleogene (K‐Pg) boundary (~66 Mya) is a key phase in amphibian evolution, associated with the origin and rapid diversification of major clades like Hyloidea, Ranoidea, and Microhylidae. While Roelants et al. (2007) inferred pre‐K‐Pg origins for Ranoidea and Microhylidae (using a five‐gene dataset) and post‐K‐Pg diversification for Hyloidea, Feng et al. (2017) and Hime et al. (2021) suggested that all three clades radiated explosively near the K‐Pg boundary, based on multi‐locus data. In contrast, our results, which are consistent with Zhang et al. (2013), indicate that these lineages mostly diverged before the K‐Pg boundary. These differences in timing may be due to variations in fossil calibrations, molecular datasets, or analytical methods used in different studies. This highlights the need for integrative approaches to better understand the pace of anuran diversification.

The older divergence times inferred in our study compared to prior works (e.g., Feng et al. 2017; Hime et al. 2021; Portik, Streicher, and Wiens 2023) may stem from three factors: limited outgroup sampling, dataset scale, and mitochondrial DNA saturation. First, we included only a few salamanders and caecilians, whereas Feng et al. (2017) and Hime et al. (2021) incorporated diverse outgroups (e.g., lungfish, coelacanths, amniotes), which can anchor divergence estimates more robustly. Second, our 24‐gene mitochondrial matrix, while similar to Zhang et al. (2013), is smaller than the multi‐locus nuclear‐mitochondrial datasets used in recent studies, potentially reducing phylogenetic resolution. Third, mtDNA's rapid substitution rates can lead to underestimation of deep divergences due to homoplasy, contrasting with slower‐evolving nuclear genes. As highlighted by Zhang et al. (2013), mitochondrial‐nuclear substitution rate disparities necessitate caution when interpreting divergence times. Future studies should integrate nuclear loci with broader taxon sampling to reconcile these discrepancies and refine evolutionary timelines.

### 4.3 Gene Rearrangement of the Anura Mitochondrial Genomes and Their Significance for the Phylogeny

Mitochondrial gene order rearrangements serve as valuable phylogenetic markers in amphibians, particularly Neobatrachian frogs, which exhibit more frequent and lineage‐specific rearrangements compared to other vertebrates (Kurabayashi and Sumida 2013; Xia et al. 2014; Chen et al. 2017; Zhang et al. 2021). For example, *Leptobrachium* and *Oreolalax* within the Megophryidae family shared a unique *trnW* transposition upstream of CR, which was proposed as a synapomorphy for these genera (Luo et al. 2022). Similarly, *Odorrana* species, except for *O. schmackeri* , which lacks *trnH*, exhibited a genus‐specific *trnH* transposition downstream of CR (Kakehashi et al. 2013). *Fejervarya* species were defined by a *trnL1* transposition downstream of *trnP*, forming the *TPL1F* tRNA cluster (Chen et al. 2017), while *Polypedates*, *Zhangixalus*, and *Rhacophorus* shared a *trnL1* transposition downstream of *trnT* (*TL1PF* tRNA cluster; Cui et al. 2022). The subfamily Occidozyginae (*Occidozyga*) uniquely positioned *trnN* downstream of *trnY*, and *Mantella* species shared *trnI* and *trnT* transpositions, likely synapomorphic for the genus. Representative species of *Leptobrachella* shared ‘*trnP*/*trnV’* transpositions and *trnW* loss, further emphasizing lineage‐specific patterns. However, homoplastic rearrangements were evident in phylogenetically distant species (Wake et al. 2011). The *trnM* tandem duplication (*IQMM* tRNA cluster) occurred convergently in Dicroglossidae and Megophryidae (Zhang et al. 2021), while *trnA* transposition downstream of *trnN* was commonly observed in most afrobatrachians, except *B. mossambicus* (*NWACY* tRNA cluster) (Kurabayashi and Sumida 2013; Hemmi et al. 2020). Convergent *nad5* transpositions downstream of CR appeared in Mantellidae, Rhacophoridae (excluding *Gracixalus yunnanensis* ), and Dicroglossidae (Ren et al. 2009; Chen et al. 2017), and *trnP* transpositions occurred in distantly related *E. hexadactyla* (Dicroglossidae) and *Hemisus marmoratus* (Hemisotidae), reflecting independent evolutionary events (Ren et al. 2009). These patterns highlight mitochondrial genome plasticity, where lineage‐defining synapomorphies coexist with homoplastic rearrangements driven by convergent evolution.

The evolution of mitochondrial genome in amphibians revealed shared and lineage‐specific structural dynamics. Nontandem duplications have been detected in both Archaebatrachia and Neobatrachia. For example, nontandem duplications of *trnM* were found in *Leptobrachella* (Archaebatrachia) and *Mantella* (Neobatrachia) exhibited, and nontandem duplications of CR occurred in *Leiopelma hochstetteri* , *Oreolalax schmidti* , and some Raniodea species. These duplicated elements, inserted at different genomic positions, occasionally degenerate into pseudogenes, suggesting independent mechanistic origin (Zhang et al. 2021). Gene losses were rare but phylogenetically informative. The *atp8* gene was absent in *Polypedates* and *Ischnocnema henselii* , indicating potential homoplasy (Taucce et al. 2018; Cui et al. 2022). Species‐specific losses, such as *trnA* and *trnN* in *Limnonectes bannaensis* , and *nad5* in *Rhacophorus rhodopus* (Zhang et al. 2009; Chen, Qin, et al. 2022), might reflect lineage adaptation or result from annotation errors. Low‐coverage regions or flawed sequencing strategies can lead to incorrect reports of gene loss. Although we have checked and re‐annotated all sequences used in this study, some genes located in low‐coverage regions may have been missed, such as the loss of *trnT* in *Ischnocnema guentheri* (Taucce et al. 2018). Additionally, Zhang et al. (2005) initially reported the absence of the *nad5* gene in *Polypedates megacephalus* , but this finding was later questioned by Kurabayashi et al. (2006), who attributed it to an assembly error caused by an unsuitable sequencing strategy. These cases suggested the need for stringent quality control in mitogenomic studies. As emphasized by Sangster and Luksenburg (2021), standardized validation protocols, ensuring accurate annotation, assembly, and coverage, are critical for improving data fidelity and utility in evolutionary research.

In conclusion, species exhibiting the same type of mitogenome rearrangement were often found to belong to closely related taxa, as demonstrated by previous studies on mitogenome rearrangements across avian, amphibian, and insect species (Haring et al. 2001; Zhang et al. 2021; Gaugel et al. 2023). However, some shared rearrangement events have not been lineage‐specific, suggesting convergence, parallelism, or reversals, which were termed homoplasy (Wake et al. 2011). Additionally, certain rearrangements were unique to individual species. Given the limited scope of taxonomic sampling to date, the prevalence of these rearrangements in related species remains largely uncertain. Further research is essential to gain a comprehensive understanding of the evolution of gene order and associated traits in Anura.

## 5 Conclusions

This study reconstructed the evolutionary trajectory of Anura through comprehensive mitogenomic analyses, resolving longstanding phylogenetic ambiguities and revealing dynamic patterns of mitochondrial gene order evolution. Phylogenomic reconstruction of 277 mitogenomes identified five principal clades: Leiopelmatoidea (basal), Discoglossoidea, Pipoidea, Pelobatoidea, and the crown group Neobatrachia. Archaeobatrachians were resolved as paraphyletic, with successive divergence events preceding the monophyletic Neobatrachia, which bifurcated into two subclades, a composite lineage comprising Calyptocephalellidae+Myobatrachoidea and Hyloidea, and Ranoidea. While congruent with recent phylogenies, persistent topological incongruences within Hyloidea and Ranoidea underscore unresolved complexities in their early diversification dynamics. Comparative genomic analyses revealed 58 distinct mitochondrial gene arrangement patterns, including phylogenetically informative synapomorphic rearrangements, highlighting the structural plasticity of anuran mitogenomes as both evolutionary markers and drivers of diversification. Divergence time estimation traced anuran origins to the Early‐Late Triassic boundary, with major Neobatrachian radiations occurring from the Late Cretaceous to Early Neogene, coinciding with pivotal paleoenvironmental shifts. These findings establish a robust framework for exploring mitogenomic structure‐ecological adaptation interplay and provide novel insights into the mechanistic basis of anuran diversification. The documented genomic innovations offer valuable markers for amphibian phylogenomics, inform conservation strategies for threatened lineages, and advance ecosystem‐level evolutionary syntheses.

## Author Contributions

**Jiaoying He:** conceptualization (equal), data curation (equal), formal analysis (lead), methodology (equal), validation (equal), visualization (equal), writing – original draft (lead), writing – review and editing (equal). **Zike Li:** data curation (equal), investigation (equal), methodology (equal), resources (equal), validation (equal). **Qingya Yang:** data curation (equal), investigation (equal), methodology (equal), resources (equal), validation (equal). **Mengyao Zhu:** data curation (equal), investigation (equal), methodology (equal), resources (equal), validation (equal). **Baiyun Xue:** data curation (equal), investigation (equal), methodology (equal), resources (equal), validation (equal). **Yinmeng Hou:** conceptualization (equal), data curation (equal), methodology (equal), supervision (equal), validation (equal), writing – review and editing (equal). **Ganggang Yang:** conceptualization (equal), data curation (equal), investigation (equal), methodology (equal), project administration (equal), resources (equal), supervision (equal), validation (equal), writing – review and editing (equal). **Xiaohong Chen:** conceptualization (equal), data curation (equal), funding acquisition (equal), investigation (equal), methodology (equal), project administration (equal), resources (equal), supervision (equal), validation (equal), writing – original draft (equal), writing – review and editing (lead). **Zhuo Chen:** conceptualization (lead), data curation (lead), formal analysis (lead), funding acquisition (lead), investigation (lead), methodology (equal), project administration (equal), resources (lead), software (lead), supervision (lead), validation (equal), visualization (equal), writing – original draft (equal), writing – review and editing (lead).

## Funding

This work was supported by the National Natural Science Foundation of China, 31601848, 32270440, 32570492, U21A20192.

## Disclosure

*Animal Research*: This study has not involved animal experiments.

## Conflicts of Interest

The authors declare no conflicts of interest.

## Supporting information


**Figure S1:** Phylogenetic distribution of start codon types across 13 protein‐coding genes in Anura based on maximum‐likelihood phylogeny inferred from 24NT dataset.

**Figure S2:** Phylogenetic distribution of stop codon types across 13 protein‐coding genes in Anura based on maximum‐likelihood phylogeny inferred from 24NT dataset.

**Figure S3:** Maximum likelihood phylogeny based on 24NTS dataset. Species were collapsed to the family level as described in Figure 3. Family‐level taxonomy follows AmphibiaWeb (2024).

**Figure S4:** Maximum likelihood phylogeny based on 11NT dataset. Species were collapsed to the family level as described in Figure 3. Family‐level taxonomy follows AmphibiaWeb (2024).

**Figure S5:** Maximum likelihood phylogeny based on 11AA dataset. Species were collapsed to the family level as described in Figure 3. Family‐level taxonomy follows AmphibiaWeb (2024).

**Figure S6:** Bayesians inference phylogeny based on 24NT dataset. Species were collapsed to the family level as described in Figure 3. Family‐level taxonomy follows AmphibiaWeb (2024).

**Figure S7:** Bayesians inference phylogeny based on 24NTS dataset. Species were collapsed to the family level as described in Figure 3. Family‐level taxonomy follows AmphibiaWeb (2024).

**Figure S8:** Bayesians inference phylogeny based on 11NT dataset. Species were collapsed to the family level as described in Figure 3. Family‐level taxonomy follows AmphibiaWeb (2024).

**Figure S9:** Bayesians inference phylogeny based on 11AA dataset. Species were collapsed to the family level as described in Figure 3. Family‐level taxonomy follows AmphibiaWeb (2024).

**Figure S10:** Phylogenetic relationships of frogs inferred from the 24NT dataset. The dataset was analyzed with partitioned ML methods.

**Figure S11:** Phylogenetic relationships of frogs inferred from the 24NT dataset. The dataset was analyzed with partitioned BI methods.

**Figure S12:** Phylogenetic relationships of frogs inferred from the 24NTS dataset. The dataset was analyzed with partitioned ML methods.

**Figure S13:** Phylogenetic relationships of frogs inferred from the 24NTS dataset. The dataset was analyzed with partitioned BI methods.

**Figure S14:** Phylogenetic relationships of frogs inferred from the 11NT dataset. The dataset was analyzed with partitioned ML methods.

**Figure S15:** Phylogenetic relationships of frogs inferred from the 11NT dataset. The dataset was analyzed with partitioned BI methods.

**Figure S16:** Phylogenetic relationships of frogs inferred from the 11AA dataset. The dataset was analyzed with partitioned ML methods.

**Figure S17:** Phylogenetic relationships of frogs inferred from the 11AA dataset. The dataset was analyzed with partitioned BI methods.

**Figure S18:** Evolutionary timetree of Anura derived from MCMCTREE in PAML, utilizing the concatenated nucleotide dataset that integrated 11 protein‐coding genes with all third codon positions, two rRNAs, and the concatenated 11 tRNAs (24NT dataset). The clade designations corresponded to those in Table S8. Nodes indicated by red boxes have been calibrated with prior constraint distributions, while blue boxes denoted divergences dates estimated without prior calibration constraints. The bounds of the boxes represented the 95% highest posterior density (HPD) for each node.

**Figure S19:** The gene rearrangement of Archaeobatrachia species. The gene order patterns of archaeobatrachian species were compared with Pattern 2 (labeled as the typical archaeobatrachian arrangement), respectively.

**Figure S20:** The gene rearrangement of Neobatrachia species. The gene order patterns of neobatrachian species were compared with Pattern 1 (labeled as the typical neobatrachian arrangement), respectively.

**Data S1:** Supporting Information.

**Table S1:** List of taxonomic samples and sequences used in this study.

**Table S2:** Best partitioning schemes and substitution models selected by ModelFinder.

**Table S3:** Nucleotide composition analysis based on 277 anuran species.

**Table S4:** Anura species with mitogenomes duplication or loss in this study.

**Table S5:** Start codons in 13 protein‐coding genes in mitochondrial genomes of 277 Anura species.

**Table S6:** Stop codons in 13 protein‐coding genes in mitochondrial genomes of 277 Anura species.

**Table S7:** The results of relative synonymous codon usage analysis for 13 PCGs of anuran species. All frequencies were averaged over all taxa. Relative synonymous codon usage was given in parentheses following the codon frequency. * represent stop codon.

**Table S8:** Divergence times of the major lineages analyzed in this study, estimated from Maximum likelihood phylogenetic analyses of the concatenated 24NT dataset.

## Data Availability

The datasets analyzed during the current study are available in the NCBI, with accession numbers being provided in Table S1.
