# Supplementary figures and images for: Mitochondrial Phylogenomics and Genome Evolution in Anura: Insights From Structure and Gene Order Rearrangements
Source: Ecol Evol. 2026 Mar 30;16(4):e73370. doi: 10.1002/ece3.73370 (PMC13107284; doi:10.1002/ece3.73370)

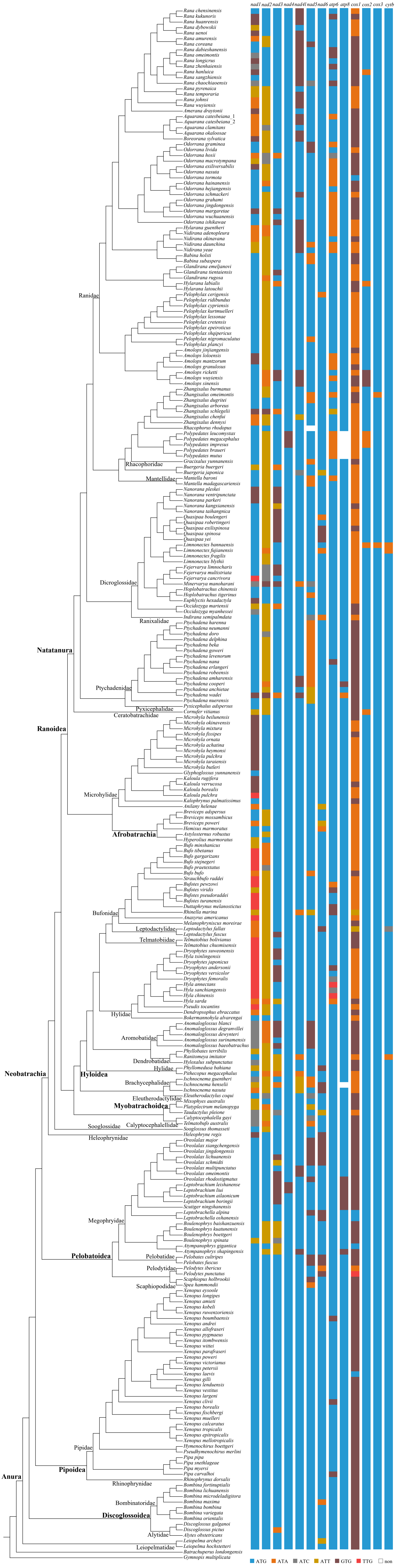

Supplement: Supplementary file 1 — Figure S1: Phylogenetic distribution of start codon types across 13 protein‐coding genes in Anura based on maximum‐likelihood phylogeny inferred from 24NT dataset. [file ECE3-16-e73370-s018.pdf]

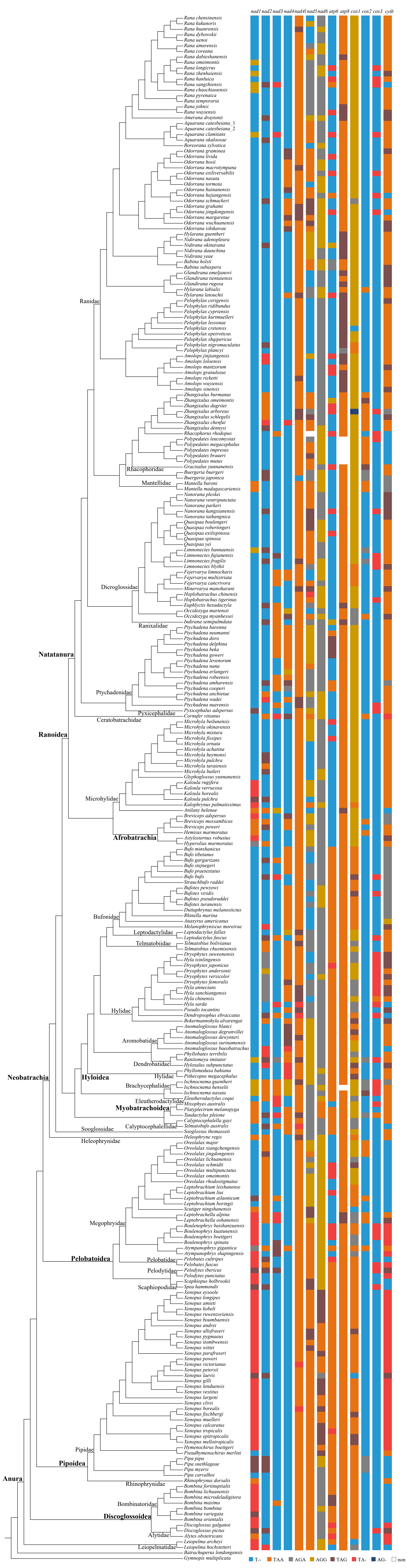

Supplement: Supplementary file 2 — Figure S2: Phylogenetic distribution of stop codon types across 13 protein‐coding genes in Anura based on maximum‐likelihood phylogeny inferred from 24NT dataset. [file ECE3-16-e73370-s024.pdf]

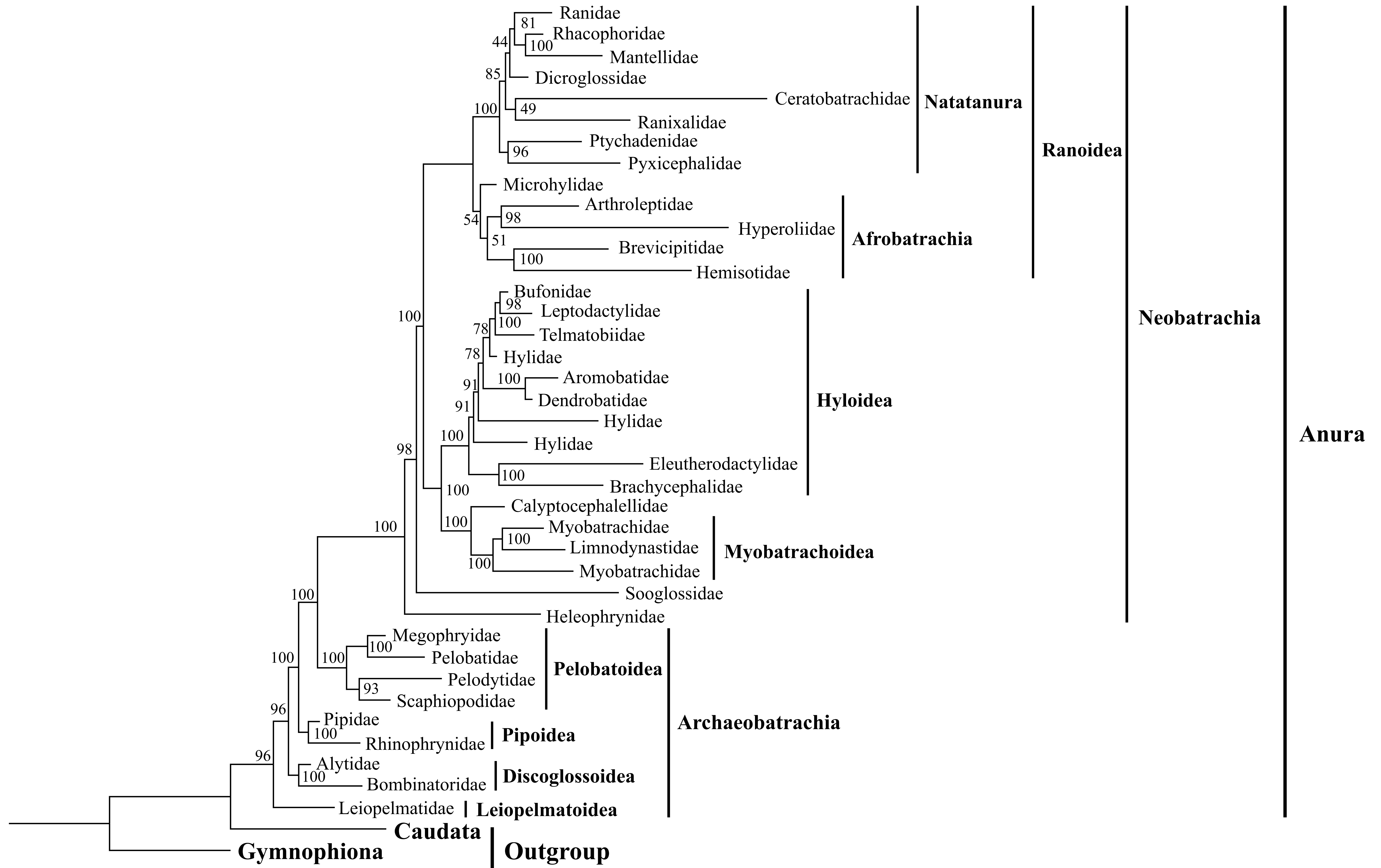

Supplement: Supplementary file 3 — Figure S3: Maximum likelihood phylogeny based on 24NTS dataset. Species were collapsed to the family level as described in Figure 3. Family‐level taxonomy follows AmphibiaWeb (2024). [file ECE3-16-e73370-s008.pdf]

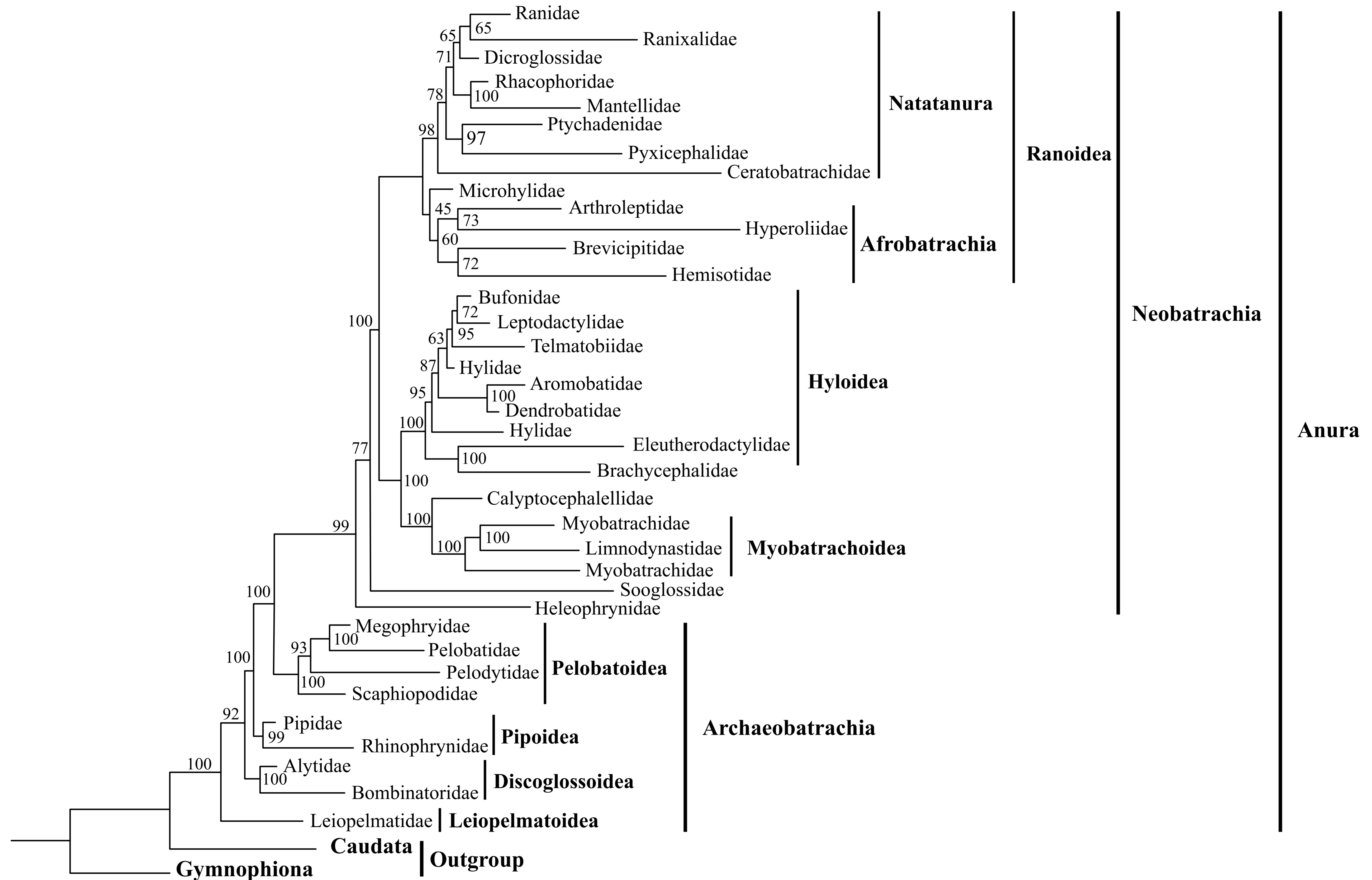

0.3

Supplement: Supplementary file 4 — Figure S4: Maximum likelihood phylogeny based on 11NT dataset. Species were collapsed to the family level as described in Figure 3. Family‐level taxonomy follows AmphibiaWeb (2024). [file ECE3-16-e73370-s011.pdf]

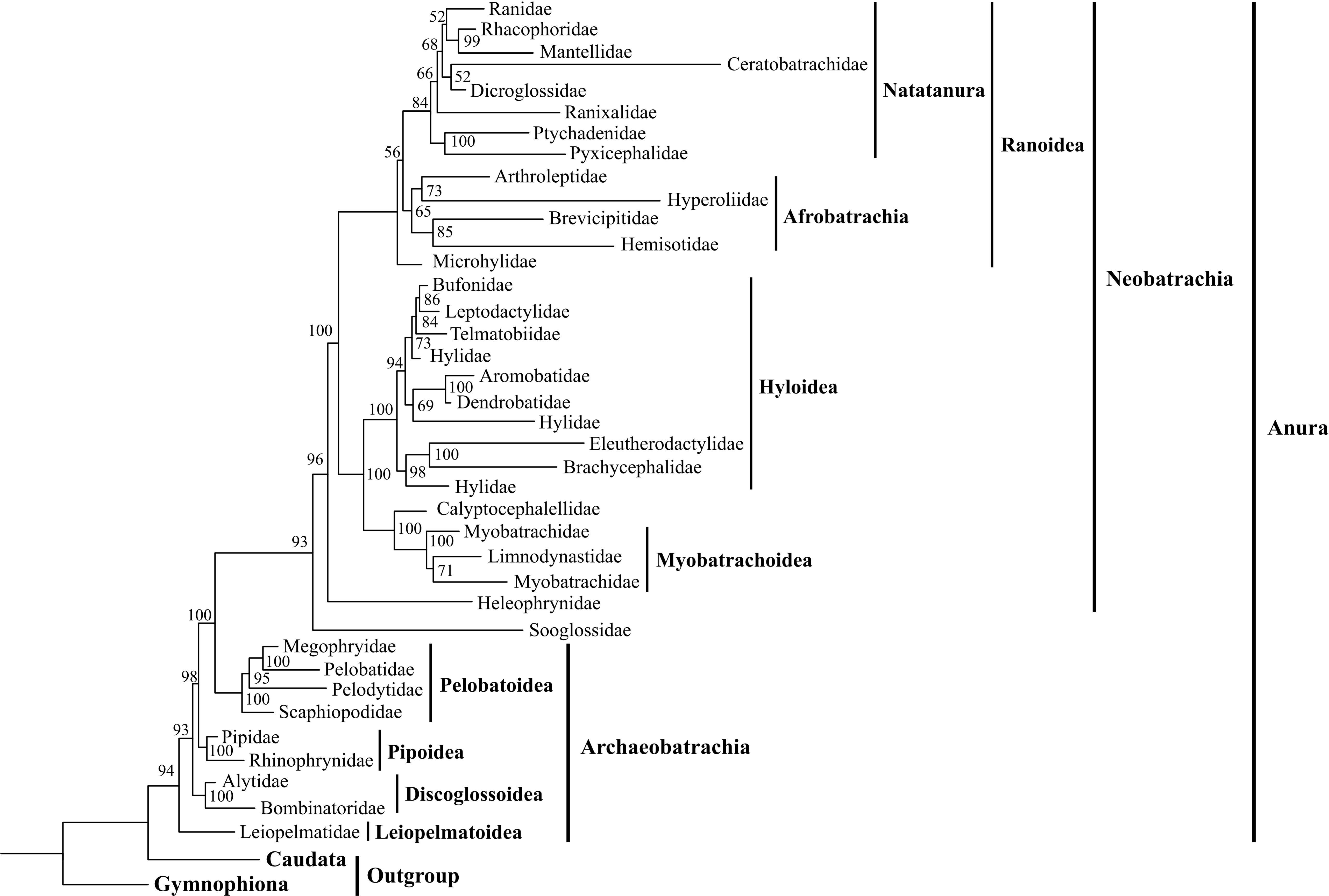

0.2

Supplement: Supplementary file 5 — Figure S5: Maximum likelihood phylogeny based on 11AA dataset. Species were collapsed to the family level as described in Figure 3. Family‐level taxonomy follows AmphibiaWeb (2024). [file ECE3-16-e73370-s028.pdf]

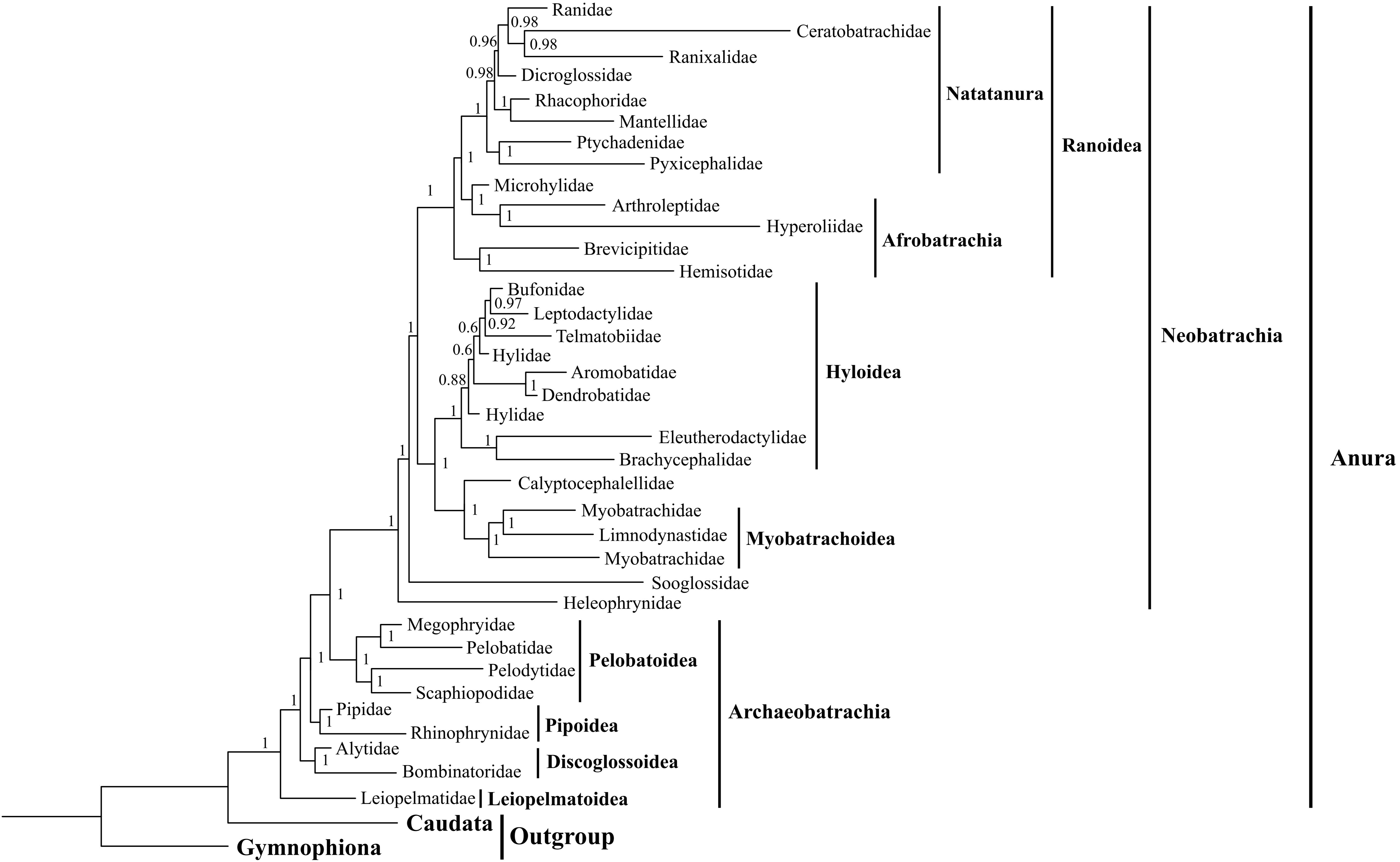

0.3

Supplement: Supplementary file 6 — Figure S6: Bayesians inference phylogeny based on 24NT dataset. Species were collapsed to the family level as described in Figure 3. Family‐level taxonomy follows AmphibiaWeb (2024). [file ECE3-16-e73370-s022.pdf]

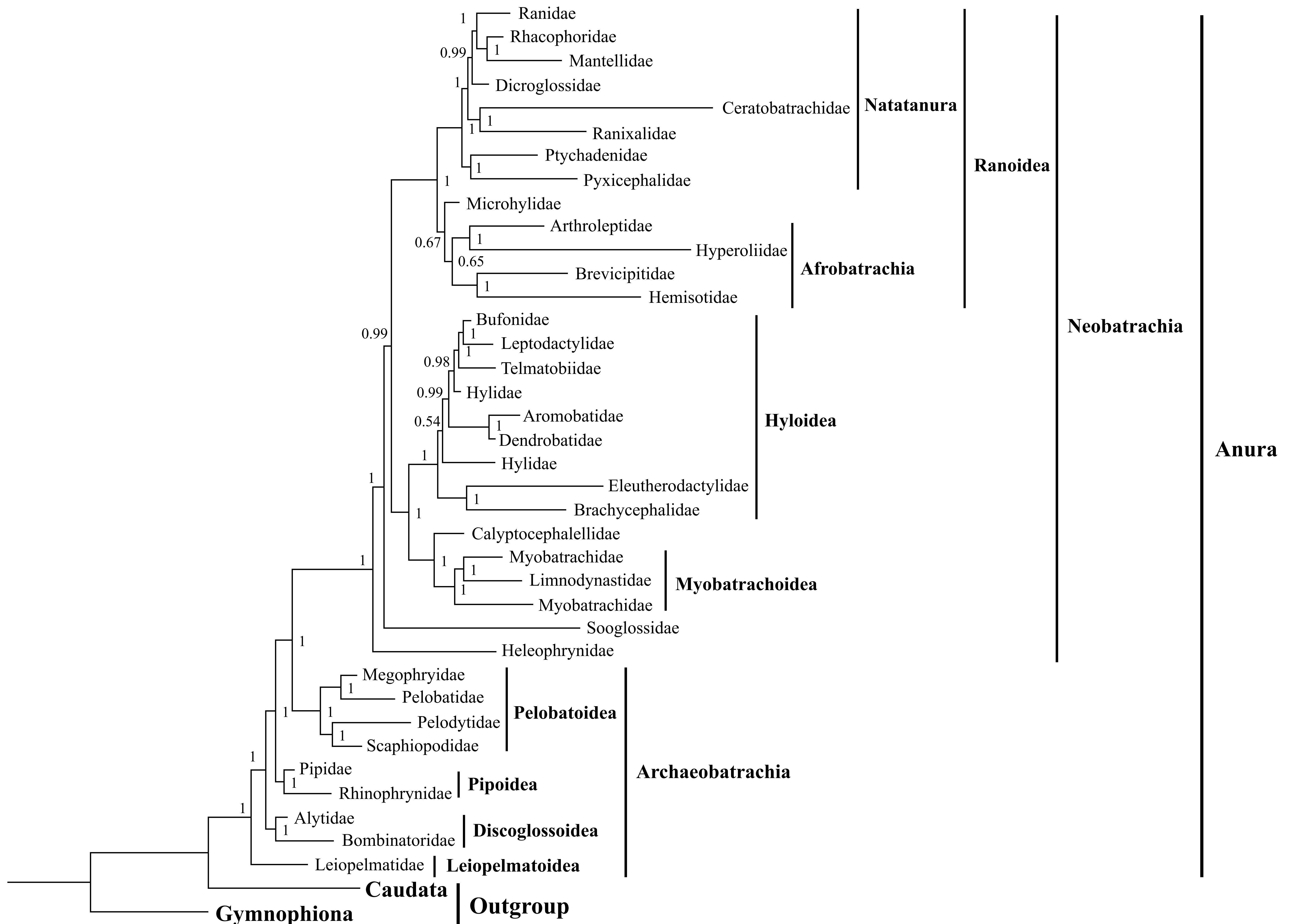

Supplement: Supplementary file 7 — Figure S7: Bayesians inference phylogeny based on 24NTS dataset. Species were collapsed to the family level as described in Figure 3. Family‐level taxonomy follows AmphibiaWeb (2024). [file ECE3-16-e73370-s007.pdf]

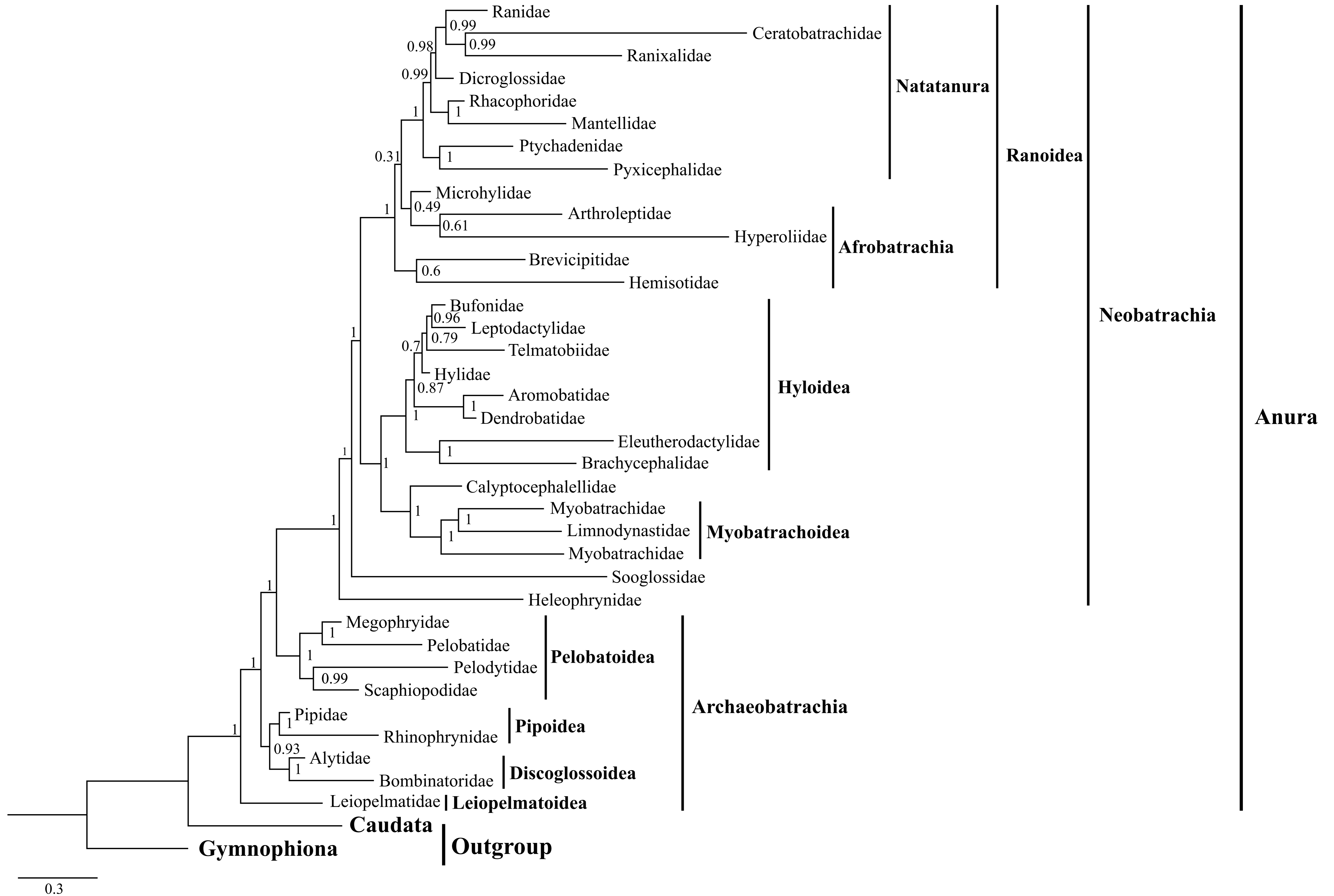

Supplement: Supplementary file 8 — Figure S8: Bayesians inference phylogeny based on 11NT dataset. Species were collapsed to the family level as described in Figure 3. Family‐level taxonomy follows AmphibiaWeb (2024). [file ECE3-16-e73370-s027.pdf]

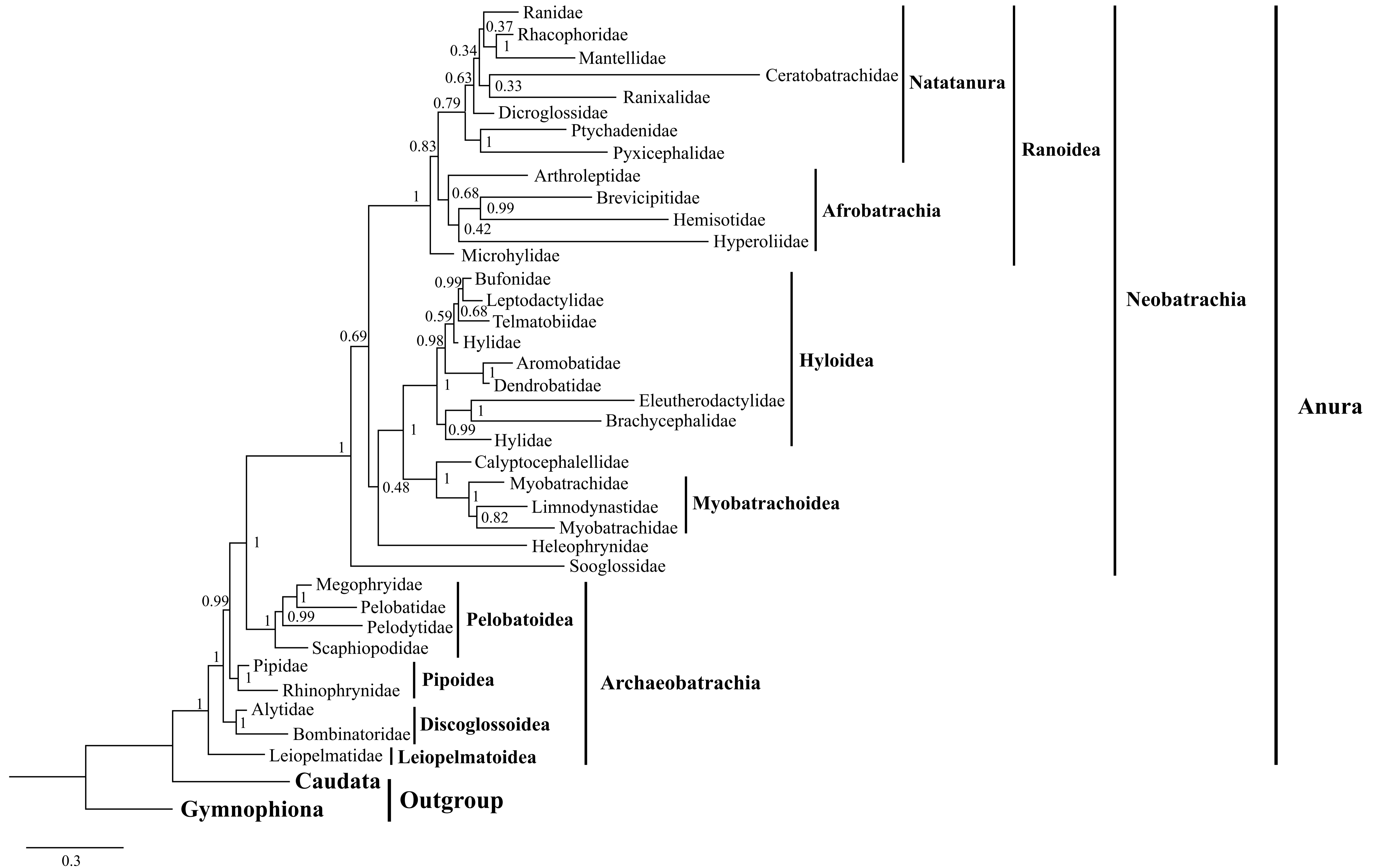

Supplement: Supplementary file 9 — Figure S9: Bayesians inference phylogeny based on 11AA dataset. Species were collapsed to the family level as described in Figure 3. Family‐level taxonomy follows AmphibiaWeb (2024). [file ECE3-16-e73370-s012.pdf]

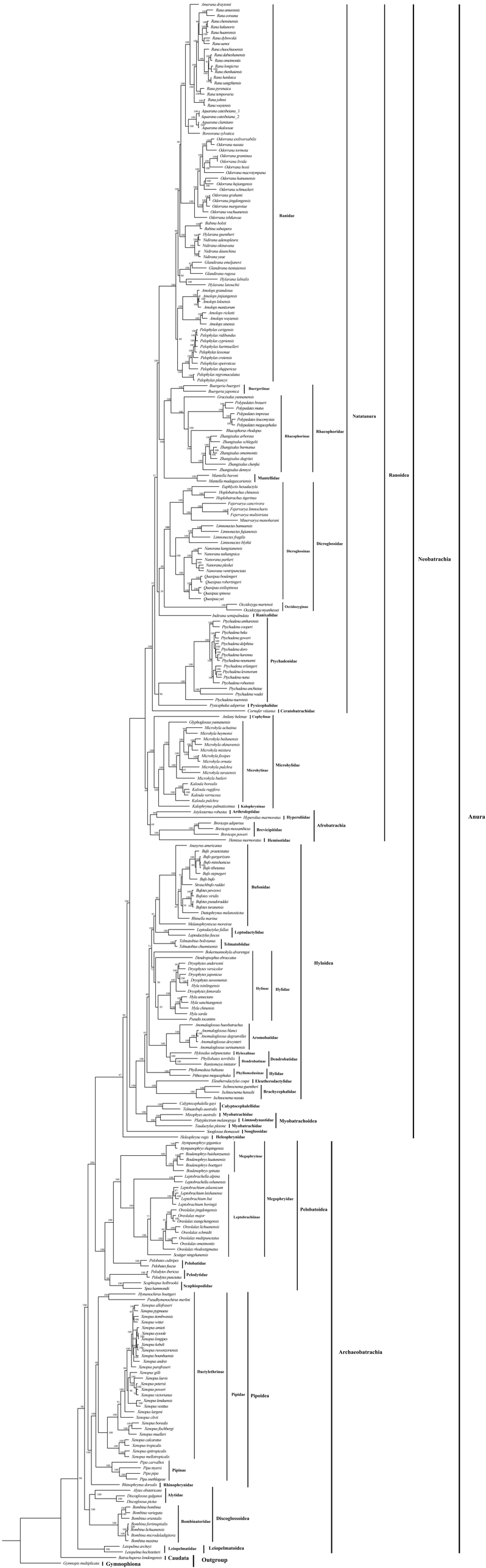

Supplement: Supplementary file 10 — Figure S10: Phylogenetic relationships of frogs inferred from the 24NT dataset. The dataset was analyzed with partitioned ML methods. [file ECE3-16-e73370-s019.pdf]

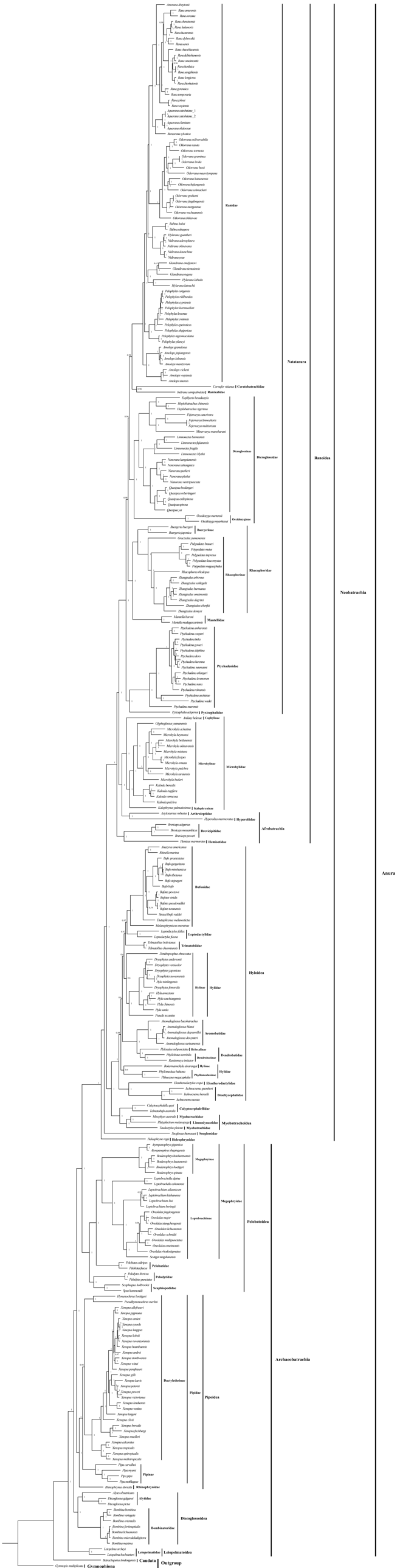

Supplement: Supplementary file 11 — Figure S11: Phylogenetic relationships of frogs inferred from the 24NT dataset. The dataset was analyzed with partitioned BI methods. [file ECE3-16-e73370-s006.pdf]

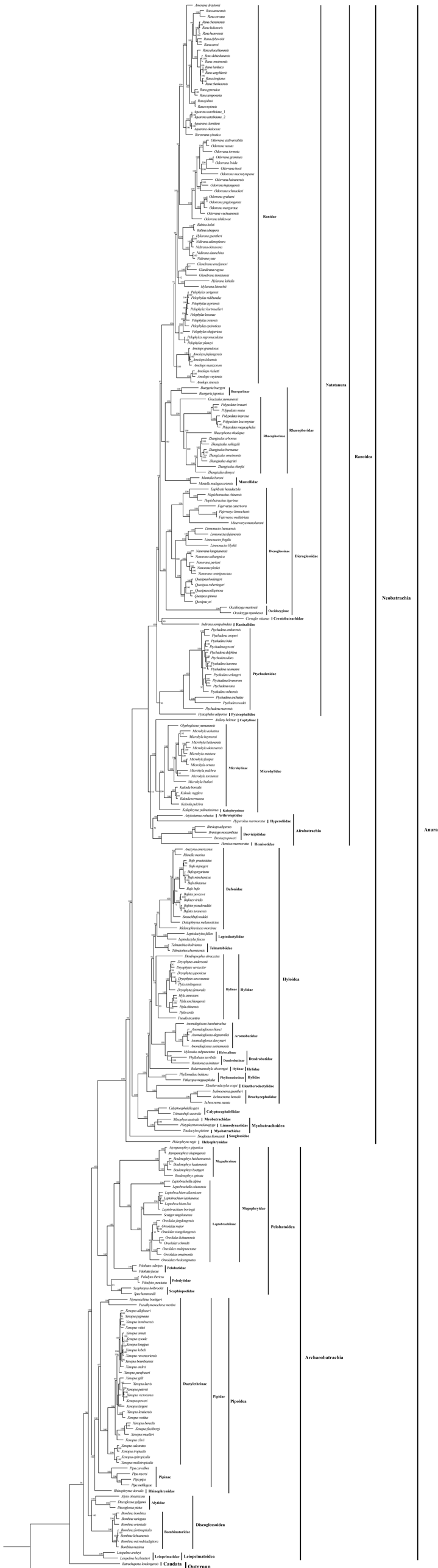

Supplement: Supplementary file 12 — Figure S12: Phylogenetic relationships of frogs inferred from the 24NTS dataset. The dataset was analyzed with partitioned ML methods. [file ECE3-16-e73370-s009.pdf]

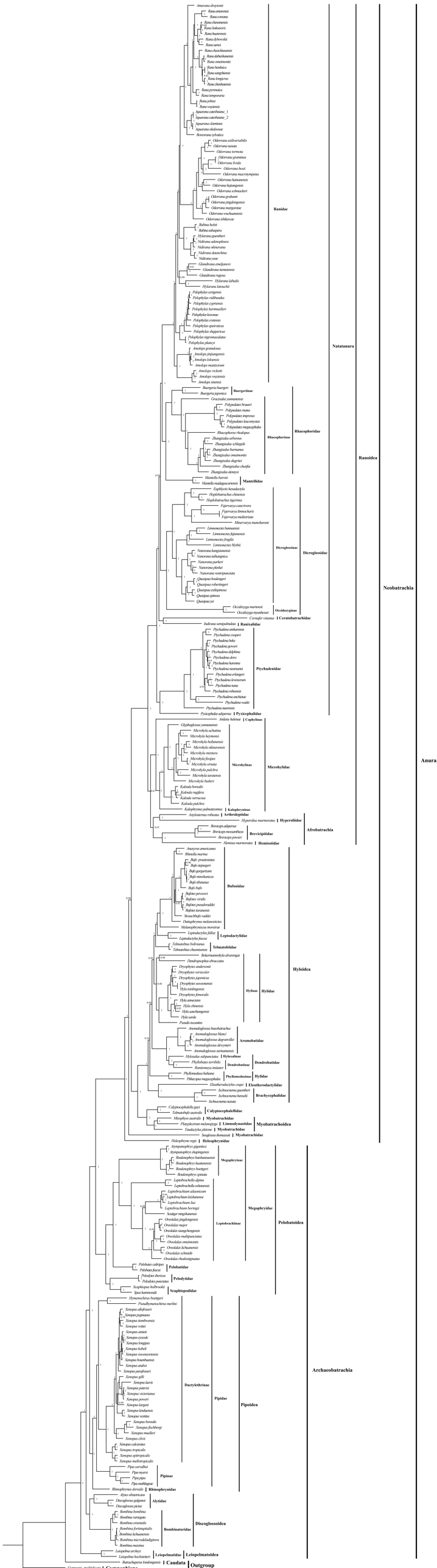

Supplement: Supplementary file 13 — Figure S13: Phylogenetic relationships of frogs inferred from the 24NTS dataset. The dataset was analyzed with partitioned BI methods. [file ECE3-16-e73370-s023.pdf]

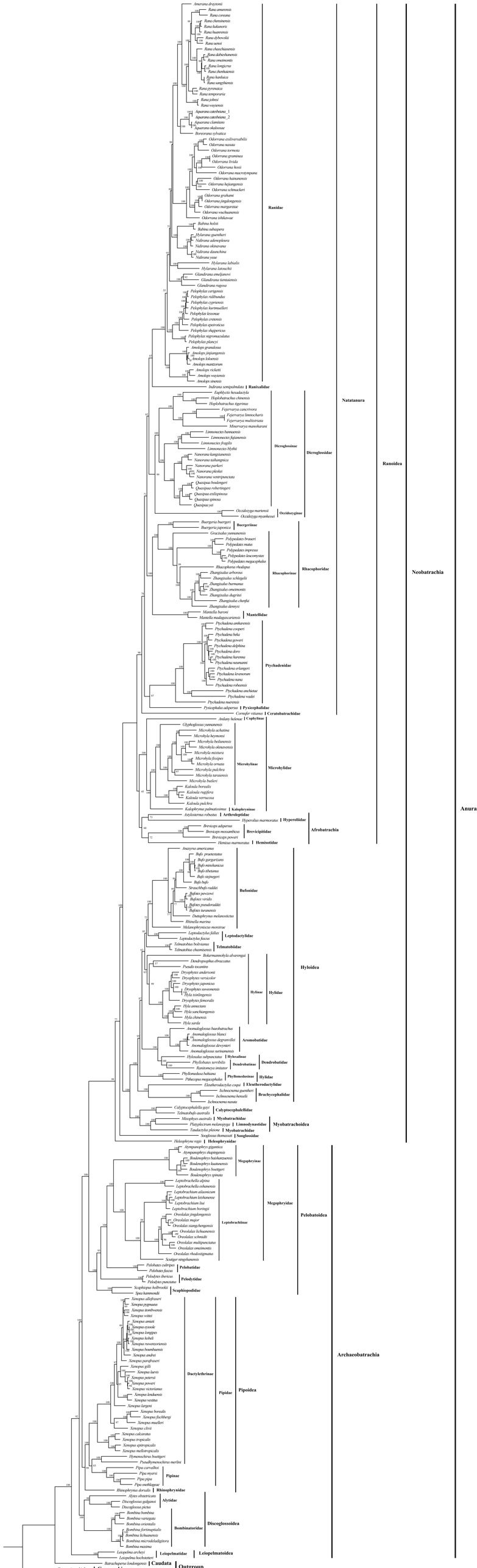

Supplement: Supplementary file 14 — Figure S14: Phylogenetic relationships of frogs inferred from the 11NT dataset. The dataset was analyzed with partitioned ML methods. [file ECE3-16-e73370-s017.pdf]

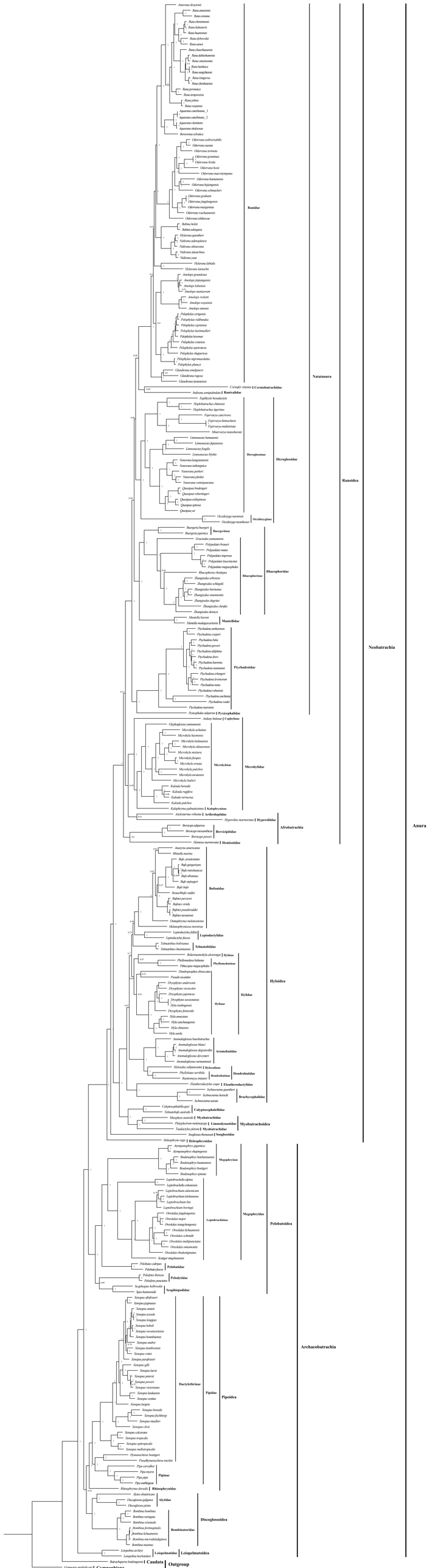

Supplement: Supplementary file 15 — Figure S15: Phylogenetic relationships of frogs inferred from the 11NT dataset. The dataset was analyzed with partitioned BI methods. [file ECE3-16-e73370-s002.pdf]

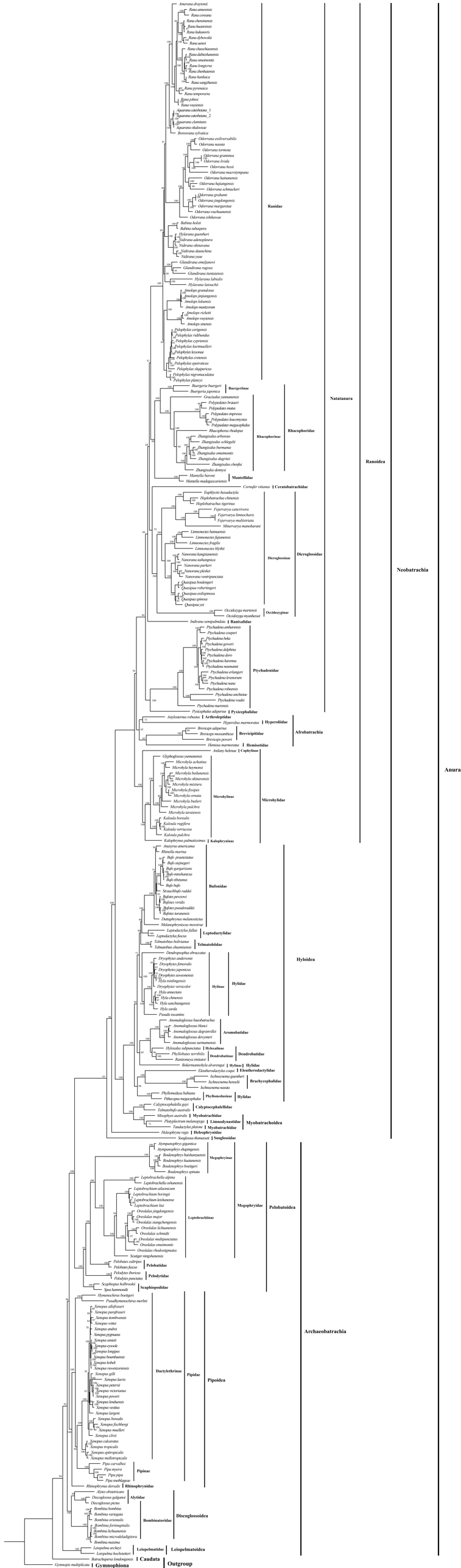

Supplement: Supplementary file 16 — Figure S16: Phylogenetic relationships of frogs inferred from the 11AA dataset. The dataset was analyzed with partitioned ML methods. [file ECE3-16-e73370-s013.pdf]

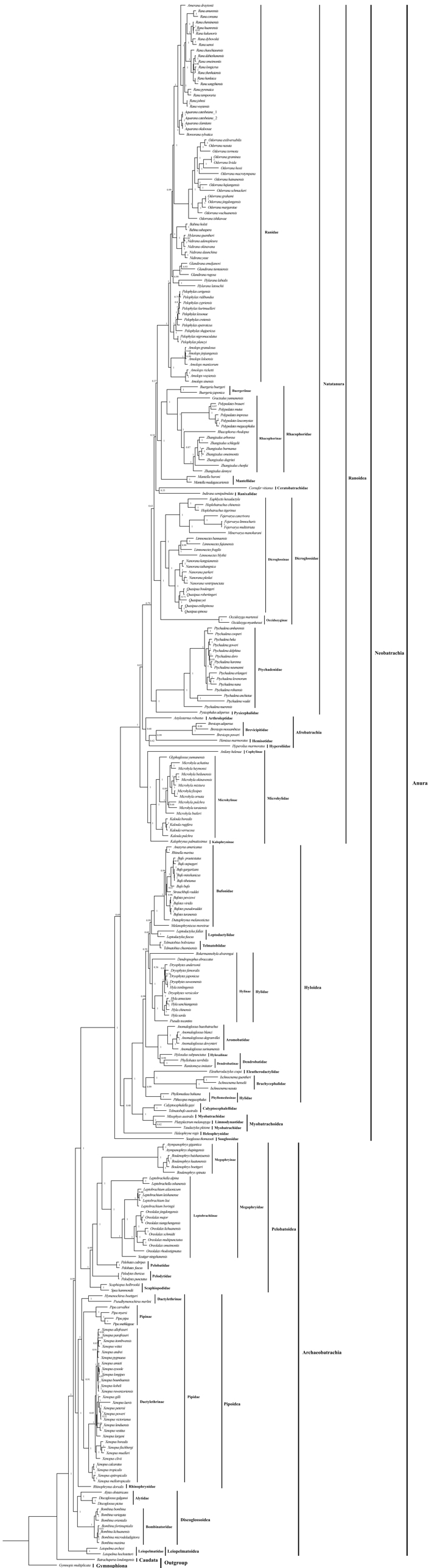

Supplement: Supplementary file 17 — Figure S17: Phylogenetic relationships of frogs inferred from the 11AA dataset. The dataset was analyzed with partitioned BI methods. [file ECE3-16-e73370-s026.pdf]
