# Supplementary material for: Mitochondrial Phylogenomics and Genome Evolution in Anura: Insights From Structure and Gene Order Rearrangements
Source: Ecol Evol. 2026 Mar 30;16(4):e73370. doi: 10.1002/ece3.73370 (PMC13107284; doi:10.1002/ece3.73370)
Supplement: Supplementary file 19 — Figure S19: The gene rearrangement of Archaeobatrachia species. The gene order patterns of archaeobatrachian species were compared with Pattern 2 (labeled as the typical archaeobatrachian arrangement), respectively. [file ECE3-16-e73370-s014.pdf]

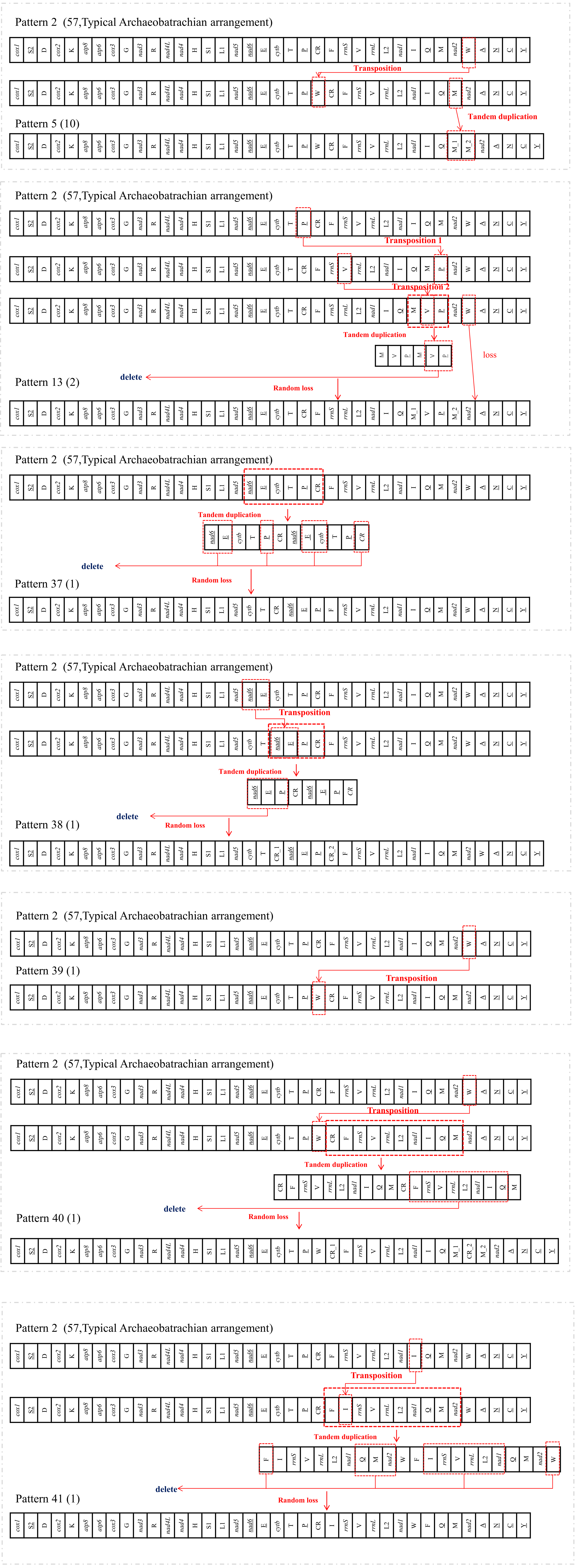

Pattern 2 (57,Typical Archaeobatrachian arrangement)
Transposition
Tandem duplication
Pattern 5 (10)
Pattern 2 (57,Typical Archaeobatrachian arrangement)
Transposition 1
Transposition 2
Tandem duplication
loss
delete
Random loss
Pattern 13 (2)
Pattern 2 (57,Typical Archaeobatrachian arrangement)
Tandem duplication
delete
Random loss
Pattern 37 (1)
Pattern 2 (57,Typical Archaeobatrachian arrangement)
Transposition
Tandem duplication
delete
Random loss
Pattern 38 (1)
Pattern 2 (57,Typical Archaeobatrachian arrangement)
Pattern 39 (1)
Transposition
Pattern 2 (57,Typical Archaeobatrachian arrangement)
Transposition
Tandem duplication
delete
Random loss
Pattern 40 (1)
Pattern 2 (57,Typical Archaeobatrachian arrangement)
Transposition
Tandem duplication
delete
Random loss
Pattern 41 (1)
