# Supplementary material for: Mitochondrial Phylogenomics and Genome Evolution in Anura: Insights From Structure and Gene Order Rearrangements
Source: Ecol Evol. 2026 Mar 30;16(4):e73370. doi: 10.1002/ece3.73370 (PMC13107284; doi:10.1002/ece3.73370)
Supplement: Supplementary file 20 — Figure S20: The gene rearrangement of Neobatrachia species. The gene order patterns of neobatrachian species were compared with Pattern 1 (labeled as the typical neobatrachian arrangement), respectively. [file ECE3-16-e73370-s015.pdf]

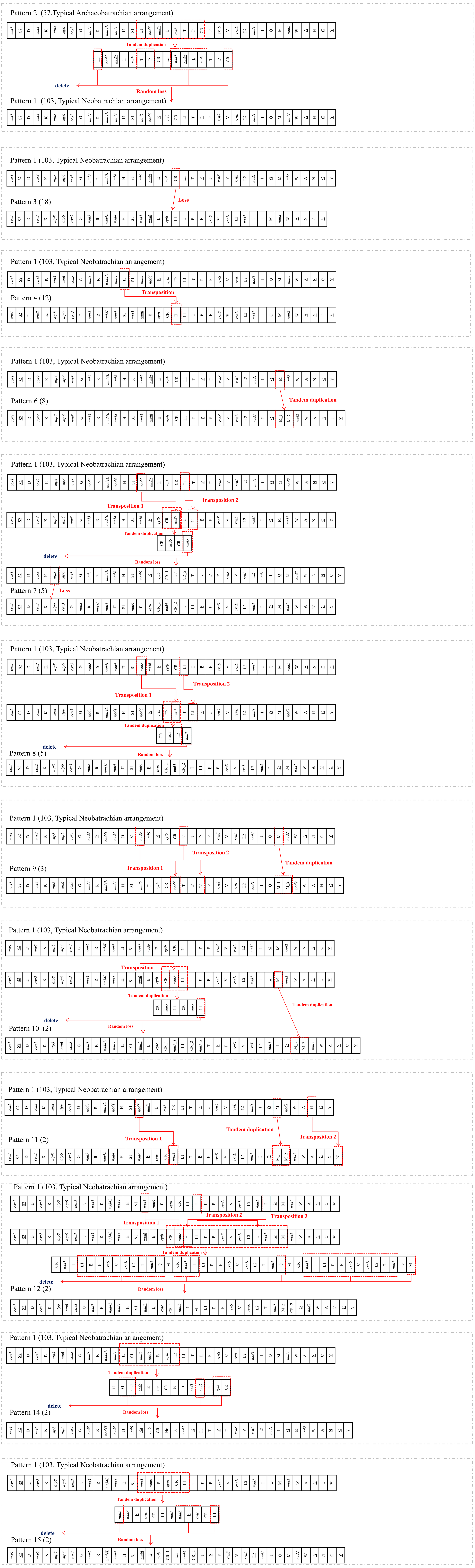

Pattern 2 (57,Typical Archaeobatrachian arrangement)
Tandem duplication
delete
Random loss
Pattern 1 (103, Typical Neobatrachian arrangement)
Pattern 1 (103, Typical Neobatrachian arrangement)
Loss
Pattern 3 (18)
Pattern 1 (103, Typical Neobatrachian arrangement)
Transposition
Pattern 4 (12)
Pattern 1 (103, Typical Neobatrachian arrangement)
Tandem duplication
Pattern 6 (8)
Pattern 1 (103, Typical Neobatrachian arrangement)
Transposition 1
Transposition 2
Tandem duplication
delete
Random loss
Pattern 7 (5)
Loss
Pattern 1 (103, Typical Neobatrachian arrangement)
Transposition 2
Transposition 1
Tandem duplication
delete
Pattern 8 (5)
Random loss
Pattern 1 (103, Typical Neobatrachian arrangement)
Transposition 2
Transposition 1
Tandem duplication
Pattern 9 (3)
Pattern 1 (103, Typical Neobatrachian arrangement)
Transposition
Tandem duplication
Tandem duplication
delete
Random loss
Pattern 10 (2)
Pattern 1 (103, Typical Neobatrachian arrangement)
Tandem duplication
Transposition 1
Transposition 2
Pattern 11 (2)
Pattern 1 (103, Typical Neobatrachian arrangement)
Transposition 2
Transposition 3
Transposition 1
Tandem duplication
delete
Random loss
Pattern 12 (2)
Pattern 1 (103, Typical Neobatrachian arrangement)
Tandem duplication
delete
Random loss
Pattern 14 (2)
Pattern 1 (103, Typical Neobatrachian arrangement)
Tandem duplication
delete
Random loss
Pattern 15 (2)

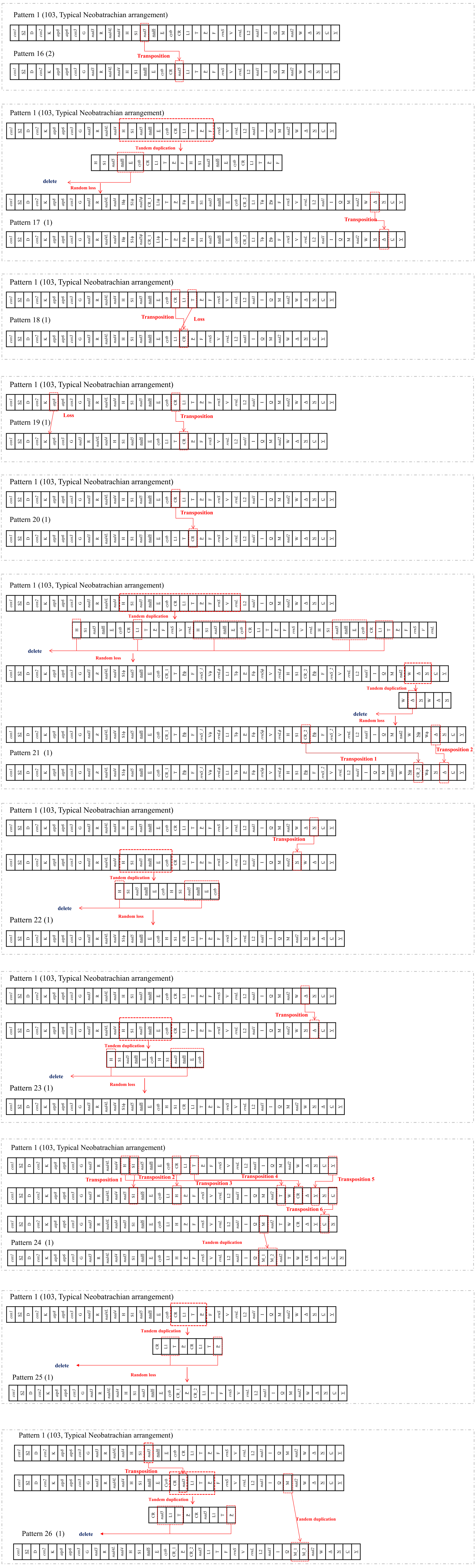

Pattern 1 (103, Typical Neobatrachian arrangement)
Pattern 16 (2)
Transposition
Pattern 1 (103, Typical Neobatrachian arrangement)
Tandem duplication
delete
Random loss
Transposition
Pattern 17 (1)
Pattern 1 (103, Typical Neobatrachian arrangement)
Pattern 18 (1)
Transposition
Loss
Pattern 1 (103, Typical Neobatrachian arrangement)
Loss
Pattern 19 (1)
Transposition
Pattern 1 (103, Typical Neobatrachian arrangement)
Transposition
Pattern 20 (1)
Pattern 1 (103, Typical Neobatrachian arrangement)
Tandem duplication
delete
Random loss
Tandem duplication
delete
Random loss
Transposition 1
Transposition 2
Pattern 21 (1)
Pattern 1 (103, Typical Neobatrachian arrangement)
Transposition
Tandem duplication
delete
Random loss
Pattern 22 (1)
Pattern 1 (103, Typical Neobatrachian arrangement)
Transposition
Tandem duplication
delete
Random loss
Pattern 23 (1)
Pattern 1 (103, Typical Neobatrachian arrangement)
Transposition 1
Transposition 2
Transposition 3
Transposition 4
Transposition 5
Transposition 6
Pattern 24 (1)
Tandem duplication
Pattern 1 (103, Typical Neobatrachian arrangement)
Tandem duplication
delete
Random loss
Pattern 25 (1)
Pattern 1 (103, Typical Neobatrachian arrangement)
Transposition
Tandem duplication
Tandem duplication
Pattern 26 (1)
delete

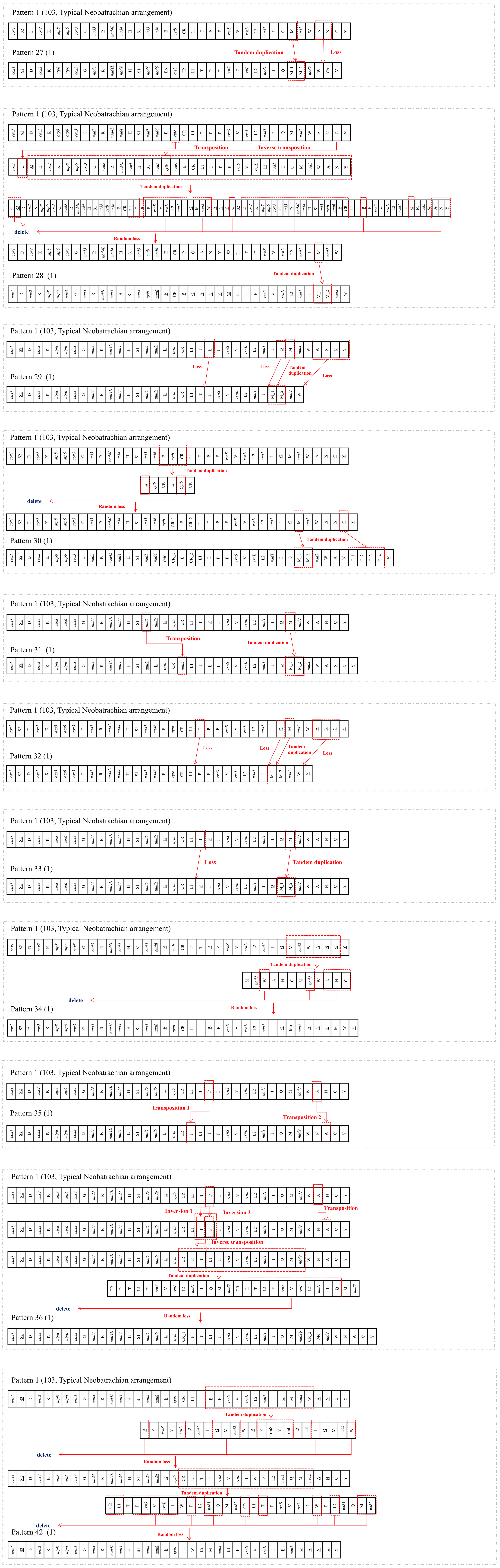

Pattern 1 (103, Typical Neobatrachian arrangement)
Pattern 27 (1)
Tandem duplication
Loss
Pattern 1 (103, Typical Neobatrachian arrangement)
Transposition
Inverse transposition
Tandem duplication
delete
Random loss
Pattern 28 (1)
Tandem duplication
Pattern 1 (103, Typical Neobatrachian arrangement)
Loss
Loss
Tandem duplication
Loss
Pattern 29 (1)
Pattern 1 (103, Typical Neobatrachian arrangement)
Tandem duplication
delete
Random loss
Pattern 30 (1)
Tandem duplication
Pattern 1 (103, Typical Neobatrachian arrangement)
Transposition
Tandem duplication
Pattern 31 (1)
Pattern 1 (103, Typical Neobatrachian arrangement)
Loss
Loss
Tandem duplication
Loss
Pattern 32 (1)
Pattern 1 (103, Typical Neobatrachian arrangement)
Loss
Tandem duplication
Pattern 33 (1)
Pattern 1 (103, Typical Neobatrachian arrangement)
Tandem duplication
delete
Random loss
Pattern 34 (1)
Pattern 1 (103, Typical Neobatrachian arrangement)
Transposition 1
Transposition 2
Pattern 35 (1)
Pattern 1 (103, Typical Neobatrachian arrangement)
Inversion 1
Inversion 2
Transposition
Inverse transposition
Tandem duplication
delete
Random loss
Pattern 36 (1)
Pattern 1 (103, Typical Neobatrachian arrangement)
Tandem duplication
delete
Random loss
Tandem duplication
delete
Random loss
Pattern 42 (1)

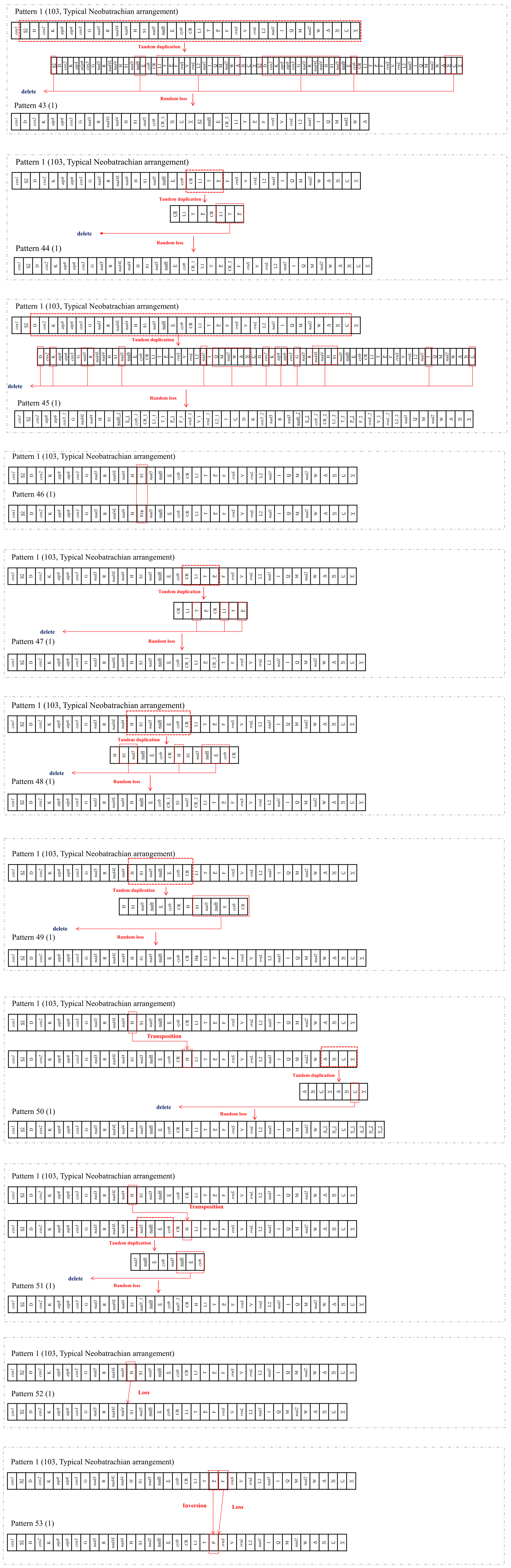
Pattern 1 (103, Typical Neobatrachian arrangement)
Tandem duplication
Random loss
delete
Pattern 43 (1)
Pattern 1 (103, Typical Neobatrachian arrangement)
Tandem duplication
delete
Random loss
Pattern 44 (1)
Pattern 1 (103, Typical Neobatrachian arrangement)
Tandem duplication
delete
Random loss
Pattern 45 (1)
Pattern 1 (103, Typical Neobatrachian arrangement)
Pattern 46 (1)
Pattern 1 (103, Typical Neobatrachian arrangement)
Tandem duplication
delete
Random loss
Pattern 47 (1)
Pattern 1 (103, Typical Neobatrachian arrangement)
Tandem duplication
delete
Random loss
Pattern 48 (1)
Pattern 1 (103, Typical Neobatrachian arrangement)
Tandem duplication
delete
Random loss
Pattern 49 (1)
Pattern 1 (103, Typical Neobatrachian arrangement)
Transposition
Tandem duplication
delete
Random loss
Pattern 50 (1)
Pattern 1 (103, Typical Neobatrachian arrangement)
Transposition
Tandem duplication
delete
Random loss
Pattern 51 (1)
Pattern 1 (103, Typical Neobatrachian arrangement)
Loss
Pattern 52 (1)
Pattern 1 (103, Typical Neobatrachian arrangement)
Inversion
Loss
Pattern 53 (1)

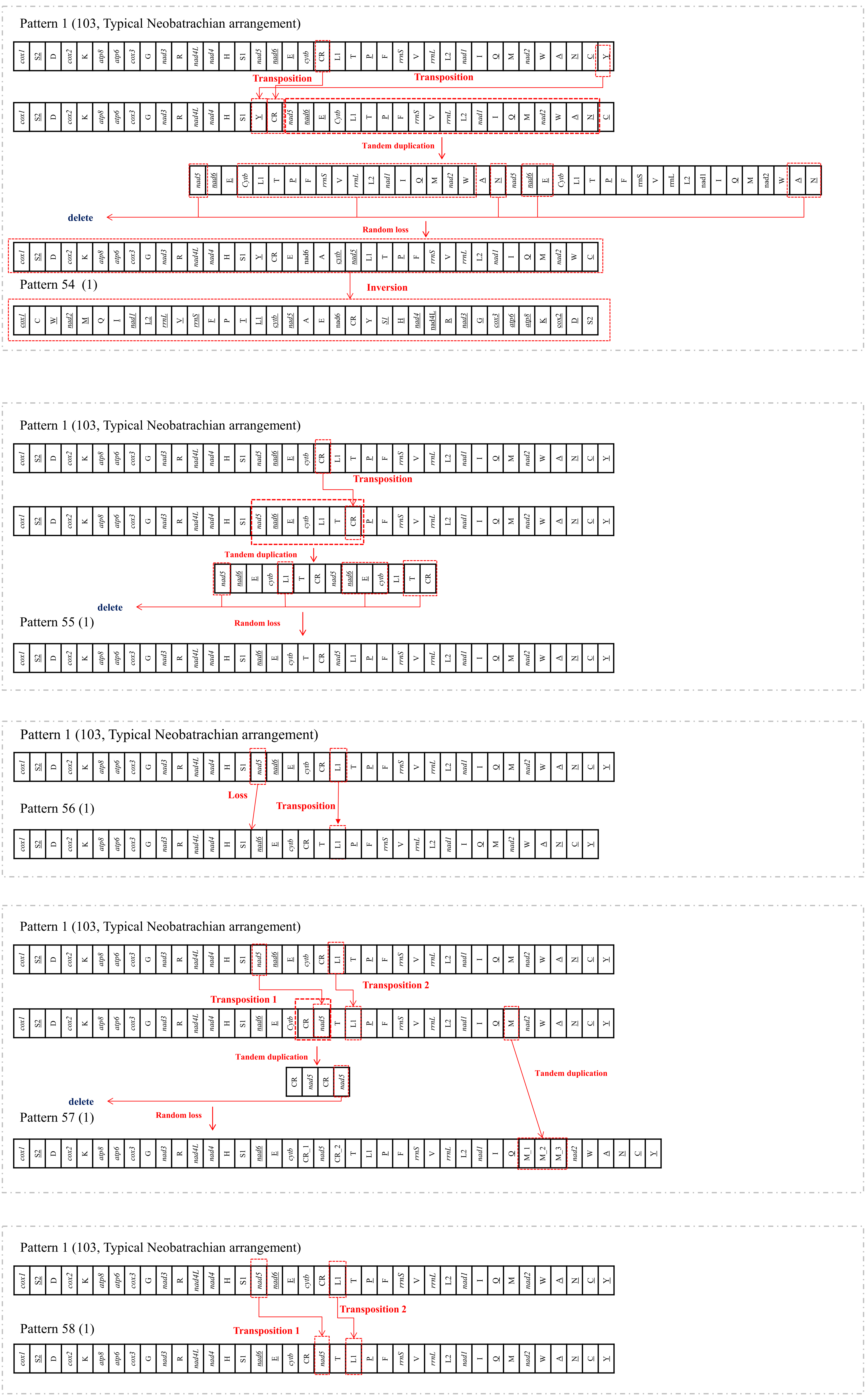
Pattern 1 (103, Typical Neobatrachian arrangement)
Transposition
Transposition
Tandem duplication
delete
Random loss
Pattern 54 (1)
Inversion
Pattern 1 (103, Typical Neobatrachian arrangement)
Transposition
Tandem duplication
delete
Random loss
Pattern 55 (1)
Pattern 1 (103, Typical Neobatrachian arrangement)
Loss
Transposition
Pattern 56 (1)
Pattern 1 (103, Typical Neobatrachian arrangement)
Transposition 2
Transposition 1
Tandem duplication
Tandem duplication
delete
Random loss
Pattern 57 (1)
Pattern 1 (103, Typical Neobatrachian arrangement)
Transposition 2
Transposition 1
Pattern 58 (1)
