# Supplementary material for: Mitochondrial Phylogenomics and Genome Evolution in Anura: Insights From Structure and Gene Order Rearrangements
Source: Ecol Evol. 2026 Mar 30;16(4):e73370. doi: 10.1002/ece3.73370 (PMC13107284; doi:10.1002/ece3.73370)
Supplement: Supplementary file 21 — Data S1: Supporting Information. [file ECE3-16-e73370-s025.docx]

**Supplementary Results**

Phylogenetic relationships among Anura

The sampled anuran frogs formed a strongly supported monophyletic group, with five major clades consistently identified across most analyses of the four datasets (24NT, 24NTS, 11NT, and 11AA), except for the BI analysis of the 11NT dataset. These clades include Leiopelmatoidea (Leiopelmatidae), Discoglossoidea (Alytidae + Bombinatoridae), Pipoidea (Rhinophrynidae + Pipidae), Pelobatoidea (Scaphiopodidae + Pelodytidae + Pelobatidae + Megophryidae), and Neobatrachia (Figures 3 and S3-S9). In contrast, the BI analysis based on the 11NT dataset recovered an alternative grouping, Discoglossoidea + Pipoidea, though with weak support (PP=0.93; Figure S6). Archaeobatrachian frogs were consistently resolved as successively branching lineages, with Leiopelmatoidea diverging first, followed by either Discoglossoidea, Pipoidea, and Pelobatoidea (24NT, 24NTS, 11AA datasets, and ML tree of 11NT) or Discoglossoidea + Pipoidea, and Pelobaroidea (BI tree of 11NT) (Figures 3 and S3-S9). The monophyly of the sampled archaeobatrachian families and genera was strongly supported across all analyses of the four datasets (Figures 3 and S3-S9). Topologies derived from the 24NT and 24NTS datasets, as well as the BI tree of the 11NT dataset, consistently divided the superfamily Pelobatoidea into two distinct clades: one comprising Scaphiopodidae and Pelodytidae, and the other containing Pelobatidae and Megophryidae (Figures 3, S3 and S6-S8). However, the 11AA dataset and ML analysis of the 11NT dataset reconstructed the relationships among these families as (Scaphiopodidae, (Pelodytidae, (Pelobatidae, Megophryidae))) (Figures S4, S5 and S9).

Phylogenetic analyses resolved Neobatrachia as the dominant clade, with family-level relationships showing dataset-dependent variations. When analyzing nucleotide datasets (24NT, 24NTS, and 11NT), Heleophrynidae (*Heleophryne regis*) was identified as the sister group to all other neobatrachian species, with Sooglossidae subsequently diverging from the remaining neobatrachians (Figures 3, S3, S4, and S6-S8). In contrast, ML analyses of amino acid datasets, however, inverted this order, placing Sooglossidae basal to Heleophrynidae (Figures S5 and S9). BI analyses of amino acid data weakly grouped Heleophrynidae with a clade Calyptocephatellidae + Myobatrachoidea + Hyloidea (PP=0.48, Figure S9). Despite these topological conflicts, two major neobatrachian lineages were consistently recovered across datasets (excluding BI analyses of the 11AA dataset): one comprising Calyptocephatellidae + Myobatrachoidea + Hyloidea and the other constituting the Ranoidea lineage (Figures 3 and S3-S9).

Within Neobatrachia, Calyptocephatellidae and Myobatrachoidea were consistently resolved as sister taxa to a clade comprising Hyloidea, with strong supports across all analyses (BP=100, PP=1.00; Figures 3 and S3-S9). Within Hyloidea, nucleotide datasets resolved Eleutherodactylidae + Brachycephalidae as basal split (BP=100, PP=1.00, Figures 3, S3, S4, and S6-S8), with remaining hyloids forming a group comprising Aromobatidae, Dendrobatidae, Leptodactylidae, Hylidae, Bufonidae, and Telmatobiidae. Relationships within these taxa varied slightly depending on the dataset and analytical method. For nucleotide datasets, Phyllomedusinae (Hylidae) or Phyllomedusinae + *Bokermannohyla alvarengai* (Hylidae) occupied the basal position in Hylidae, depending on the analysis (e.g., ML analyses of 24NT and 11NT datasets, BI analysis of the 24NTS dataset). In contrast, amino acid tress grouped Phyllomedusinae (Hylidae) with Eleutherodactylidae + Brachycephalidae, forming the sister clade to all other hyloids (Figures S5 and S9). Consistently, Aromobatidae and Dendrobatidae were recovered as sister taxa across all analyses (BP = 100, PP = 1.00; Figures 3 and S3–S9), while the remaining families followed the topology: (Hylidae, (Telmatobiidae, (Leptodactylidae, Bufonidae))). All families except Hylidae were monophyletic. Hylidae exhibited paraphyly in most analyses due to the early divergence of Phyllomedusinae or Phyllomedusinae + *Bokermannohyla alvarengai* (Figures 3, S3–S7, S9–S14, S16, and S17), with only BI analysis of the 11NT dataset weakly supporting its monophyly (PP = 0.69; Figures S8 and S15).

Phylogenetic relationships within Ranoidea, comprising Microhylidae, Afrobatrachia (i.e., Brevicepitidae, Hemisotidae, Arthroleptidae, and Hyperoliidae), and Natatanura, varied across datasets and analytical methods. Nucleotide-based ML analyses (24NT, 11NT, 24NTS) resolved Microhylidae as the sister taxon to Afrobatrachia (Figures 3, S3–S4, and S7), while 11AA datasets (ML/BI) placed Microhylidae as the sister group to (Afrobatrachia + Natatanura) (Figures S5 and S9). Afrobatrachia itself was inconsistently supported: ML analyses weakly supported its monophyly (Figures 3, S3–S5, S7, S9), but BI trees for 24NT and 11NT datasets rendered it polyphyletic, with Brevicepitidae + Hemisotidae diverging first and the remaining Afrobatrachia (Arthroleptidae + Hyperoliidae) grouping with Microhylidae + Natatanura (Figures S6 and S8). Internal relationships within Afrobatrachia largely followed ((Arthroleptidae, Hyperoliidae), (Brevicepitidae, Hemisotidae)) across most analyses, except BI trees (24NT, 11NT, 11AA), where Arthroleptidae emerged as the basal lineage in 11AA dataset (Figure S9).

The Natatanura clade, a major radiation of extant anurans, comprised two primary groups in our study, i.e., African-centered lineages (Pyxicephalidae and Ptychadenida) and Asian-centered lineages (Ranixalidae, Dicroglossidae, Mantellidae, Ceratobatrachidae, Rhacophoridae, and Ranidae). Monophyly of both groups was supported in most analyses (Figures 3 and S3-S9), except ML analyses of the 24NT and 11NT datasets, which resolved Asian lineages as non-monophyletic due to the early divergence of Ceratobatrachidae from the African clade (Figures 3 and S4). Relationships among the Asian families were largely unresolved, except to the consistent placement of Mantellidae as the sister group to Rhacophoridae, supported by all analyses (BP=99 or 100, PP=1.00; Figures 3 and S3-S9). The clade (Rhacophoridae, Mantellidae) was grouped with Ranidae in analyses of the 24NTS and 11AA datasets, as well as in the ML analysis of the 24NT dataset (Figures 3, S3, S5, S7, and S9). In contrast, BI analyses of the 24NT and 11NT datasets placed Ceratobatrachidae + Ranixalidae with Ranidae, positioning the (Rhacophoridae, Mantellidae) clade in a basal position within the Asian families (Figures S6 and S8). Relationships among these families were complex, with most branches exhibiting weak support (Figures 3 and S3-S9). These inconsistencies, coupled with low bootstrap/posterior probabilities, suggested that denser taxon sampling or genomic data may be needed to clarify Natatanura’s internal phylogeny.

While most genera were recovered as monophyletic in our analyses, taxonomic inconsistences persisted in six genera across three families: Ranidae, Dicroglossidae, and Hylidae. Within Ranidae, *Hylarana guentheri* was nested with *Nidirana*, forming the clade ((*N. daunchina*, *N. yeae*), (*N. okinavana*, (*N. adenopleura*, *H. guentheri*))). Within Hylidae, *Hyla tsinlingensis* was grouped with *Dryophytes*, challenging its current classification.

**Supplementary discussion**

Phylogenetic Relationships among Anura

Our phylogenetic reconstruction of Anura is consistent with recent large-scale studies (Pyron et al. 2011; Zhang et al. 2013; Feng et al. 2017; Hime et al. 2021; Zhang et al. 2021; Portik et al. 2023a, b), which also recovered the deep topology (Leiopelmatoidea, (Discoglossoidea, (Pipoidea, (Pelobatoidea, Neobatrachia)))). This topology was strongly supported across datasets, except for the BI analysis of the 11NT dataset. Interestingly, Zhang et al. (2021) reported results similar to our BI topology of the 11NT dataset, where Discoglossoidea grouped with Pipoidea, and the clade (Leiopelmatoidea, (Discoglossoidea, Pipoidea)) clustered with Neobatrachia. This discrepancy may arise from differences in taxon sampling, such as the absence of Pelobatoidea in Zhang et al. (2021), and molecular marker selection. Within Pelobatoidea, the nucleotide and amino acid datasets of protein-coding genes (excluding the BI analysis of the 11NT dataset) resolved Scaphiopodidae as the basal group, followed by Pelodytidae, Pelobatidae, and Megophryidae, consistent with prior studies (Pyron et al. 2011; Zhang et al. 2013; Feng et al. 2017; Hime et al. 2021; Portik et al. 2023a, b). In contrast, the 24NT, 24NTS, and BI analyses of the 11NT dataset grouped Scaphiopodidae + Pelodytidae as sister to Pelobatidae + Megophryidae, highlighting dataset sensitivity in resolving intra-Peobatoidea relationships.

Within Neobatrachia, our nucleotide datasets strongly supported Heleophrynidae as the sister lineage to all other Neobatrachians, consistent with recent phylogenies (Pyron et al. 2011; Zhang et al. 2013; Feng et al. 2017; Jetz and Pyron 2018; Hime et al. 2021; Zhang et al. 2021; Portik et al. 2023a, b). Similarly, Sooglossidae was resolved as the next divergent clade after Heleophrynidae, aligning with Pyron et al. (2011) and Zhang et al. (2021), but conflicting with studies placing Sooglossidae sister to Ranoidea (Zhang et al. 2013; Feng et al. 2017; Jetz and Pyron 2018; Hime et al. 2021; Portik et al. 2023b). The clade Calyptocephalellidae + Myobatrachoidea was recovered as sister to Hyloidea in our analyses, corroborating the findings from other studies (e.g., Feng et al. 2017; Jetz and Pyron 2018; Hime et al. 2021; Zhang et al. 2021; Portik et al. 2023b), whereas Portik et al. (2023a) proposed a different relationship (Myobatrachoidea, (Calyptocephalellidae, Hyloidea)) using UCE data. Finally, Ranoidea was robustly monophyletic and sister to (Hyloidea, (Myobatrachoidea, Calyptocephalellidae)), resolving a key node with high support.

Within Hyloidea, our analyses (excluding the 11AA dataset) strongly supported Eleutherodactylidae + Brachycephalidae as the sister group to all other hyloid frogs, which is consistent with previous studies (Darst and Cannatella 2004; Frost et al. 2006; Roelants et al. 2007; Pyron et al. 2011; Zhang et al. 2013). However, recent researches (Feng et al. 2017; Hime et al. 2021; Portik et al. 2023a, b) instead positioned Telmatobiidae as the basal lineage, with Eleutherodactylidae and Brachycephalidae diverging later. Additionally, Jetz and Pyron (2018) placed Dendrobatidae as the earliest-diverging hyloid lineage, adding to the conflicting results. In our study, Leptodactylidae and Bufonidae formed a stable clade, a result shared by most recent works (Zhang et al. 2013; Feng et al. 2017; Jetz and Pyron 2018; Hime et al. 2021; Portik et al. 2023a, b), which contrasts with Pyron et al. (2011) who found Bufonidae + Dendrobatidae, and Zhang et al. (2021), who reported Bufonidae as sister to Telmatobiidae + Hylidae. Hylidae itself showed persistent discordance: our analysis rejected its monophyly due to successive subfamily branching, while other studies upheld monophyly but with conflicting placements, either as the second-diverging hyloid lineage (Feng et al. 2017; Hime et al. 2021; Portik et al. 2023a, b) or as sister to Leptodactylidae + Bufonidae (Jetz and Pyron 2018). These unresolved relationships underscore the limited consensus on deep Hyloidea phylogeny, likely influenced by variations in taxon sampling, molecular markers, and analytical methods.

Within Ranoidea, our analyses (with the exeption of the BI analysis of the 24NT and 11NT datasets) identified three major clades: Microhylidae, Afrobatrachia, and Natatanura, a result that aligns with previous studies (Pyron et al. 2011; Zhang et al. 2013; Feng et al. 2017; Jetz and Pyron 2018; Hime et al. 2021; Zhang et al. 2021; Portik et al. 2023a, b). Nucleotide datasets indicated that Microhylidae + Afrobatrachia are sister to Natatanura, a finding that is consistent with Pyron et al. (2011), Zhang et al. (2013) and Jetz and Pyron (2018). In contrast, amino acid datasets and recent phylogenies (Feng et al. 2017; Hime et al. 2021; Zhang et al. 2021; Portik et al. 2023a, b) suggested that Microhylidae was sister to (Afrobatrachia + Natatanura). Relationships within Afrobatrachia largely reflected consensus topologies (Brevicepitidae + Hemisotidae as sister to Arthroleptidae + Hyperoliidae, Zhang et al. 2013; Feng et al. 2017; Jetz and Pyron 2018; Hime et al. 2021; Zhang et al. 2021; Portik et al. 2023a, b), with the exception of the BI analysis of the 11AA dataset, which positioned Arthroleptidae as the basal lineage.

Our analyses of the phylogenetic relationships within Natatanura revealed significant inconsistencies across different studies. Following the classification framework provided by Feng et al. (2017) and Portik et al. (2023b), we grouped the species into two main lineages: the African-centered Pyxicephalidae + Ptychadenidae and the Asian-centered lineages, which include Ranixalidae, Ceratobatrachidae, Dicroglossidae, Ranidae, Mantellidae, and Rhacophoridae. The majority of our analyses confirmed the monophyly of both lineages and indicated that the African clade diverged first within Natatanura, which was consistent with Feng et al. (2017), Hime et al. (2021), and Portik et al. (2023b). However, Jetz and Pyron (2018) and Portik et al. (2023a) proposed a paraphyletic Asian group, with Pyxicephalidae and Ptychadenidae diverging sequentially. Similarly, our BI trees from the 24NT and 11NT datasets rejected the monophyly of the Asian groups, as Ceratobatrachidae branching before the African clade, a pattern that is consistent with Zhang et al. (2013), who found Ptychadenidae nested within the Asian lineages.

The phylogenetic relationships among Asian-centered Natatanura families (Ranixalidae, Ceratobatrachidae, Dicroglossidae, Ranidae, Mantellidae, and Rhacophoridae) showed inconsistencies depending on the dataset used. The ML analysis of the 24NT dataset showed that Ranixalidae + Dicroglossidae were sister to (Ranidae, (Rhacophoridae, Mantellidae)), consistent with several recent studies (Pyron et al. 2011; Feng et al. 2017; Jetz and Pyron 2018; Hime et al. 2021; Zhang et al. 2021; Portik et al. 2023b), even though some studies in (Feng et al. 2017; Hime et al. 2021; Zhang et al. 2021) did not include Ranixalidae. The 24NTS dataset mirrored Feng et al. (2017), Hime et al. (2021), and Zhang et al. (2021) but grouped Ranixalidae + Ceratobatrachidae, in line with Portik et al. (2023a). In contrast, 11NT dataset produced divergent topologies: ML placed Rhacophoridae + Mantellidae as sister to (Dicroglossidae, (Ranidae, Ranixalidae), while BI clustered Ceratobatrachidae + Ranixalidae with Ranidae. The 11AA dataset further conflicted, swapping the positions of Ranixalidae and Dicroglossidae between ML and BI trees. These topological differences, potentially driven by lineage-specific substitutions rates and genetic distances (Ding et al. 2019), underscore the challenges in resolving rapid radiations within Natatanura using only mitochondrial data.

**References**

Ding, S. M., W. H. Li, Y. Wang, S. L. Cameron, D. Murányi, D. Yang. 2019. “The phylogeny and evolutionary timescale of stoneflies (Insecta: Plecoptera) inferred from mitochondrial genomes.” *Molecular Phylogenetics and Evolution* 135: 123–135. <https://doi.org/10.1016/j.ympev.2019.03.005>.

Darst, C. R., D. C. Cannatella. 2004. “Novel relationships among hyloid frogs inferred from 12S and 16S mitochondrial DNA sequences.” *Molecular Phylogenetics and Evolution* 31: 462–475. <https://doi.org/10.1016/j.ympev.2003.09.003>.

Feng, Y. J., D. C. Blackburn, D. Liang, D. M. Hillis, D. B. Wake, D. C. Cannatella, P. Zhang. 2017. “Phylogenomics reveals rapid, simultaneous diversification of three major clades of Gondwanan frogs at the Cretaceous-Paleogene boundary. *Proceedings of the National Academy of Sciences of the United States of America* 114, no. 29: E5864–5870. <https://doi.org/10.1073/pnas.1704632114>.

Frost, D. R., T. Grant, J. Faivovich, R. H. Bain, A. Haas, C. F. B. Haddad, R. O. De Sa, A. Channing, M. Wilkinson, S. C. Donellan, C. J. Raxworthy, J. A. Campbell, B. L. Blotto, P. Moler, R. C. Drewes, R. A. Nussbaum, J. D. Lynch, D. M. Green, A. W. C. Wheeler. 2006. “The amphibian tree of life.” *Bulletin American Museum of Natural History* 297: 1–370. [http://dx.doi.org/10.1206/0003-0090(2006)297[0001:TATOL]2.0.CO;2](http://dx.doi.org/10.1206/0003-0090(2006)297%5b0001:TATOL%5d2.0.CO;2).

Hime, P. M., A. R. Lemmon, E. C. M. Lemmon, E. Prendini, J. M. Brown, R. C. Thomson, J. D. Kratovil, B. P. Noonan, R. A. Pyron, P. L. V. Peloso, M. L. Kortyna, J. S. Keogh, S. C Donnellan, R. L. Mueller, C. J. Raxworthy, K. Kunte, S. R. Ron, S. Das, N. Gaitonde, D. M. Green, J. Labisko, J. Che, D. W. Weisrock. 2021. “Phylogenomics reveals ancient gene tree discordance in the amphibian tree of life.” *Systematic Biology* 70, no. 1: 49–66. <https://doi.org/10.1093/sysbio/syaa034>.

Jetz, W., and R. A. Pyron. 2018. “The interplay of past diversification and evolutionary isolation with present imperilment across the amphibian tree of life.” *Nature Ecology and Evolution* 2, no. 5: 850–858. <https://doi.org/10.1038/s41559-018-0515-5>.

Portik, D. M., J. W. Streicher, D. C. Blackburn, D. S. Moen, C. R. Hutter, J. J. Wiens. 2023. “Redefining possible: combining phylogenomic and supersparse data in frogs.” *Molecular Biology and Evolution* 40, no. 5: msad109. <https://doi.org/10.1093/molbev/msad109>.

Portik, D. M., J. W. Streicher, and J. J. Wiens. 2023. “Frog phylogeny: A time-calibrated, species-level tree based on hundreds of loci and 5,242 species.” *Molecular Phylogenetics and Evolution* 188: 107907. <https://doi.org/10.1016/j.ympev.2023.107907>.

Pyron, R. A., and J. J. Wiens. 2011. “A large-scale phylogeny of Amphibia including over 2800 species, and a revised classification of extant frogs, salamanders, and caecilians.” *Molecular Phylogenetics and Evolution* 61, no. 2: 543–583. <https://doi.org/10.1016/j.ympev.2011.06.012>.

Roelants, K., D. J. Gower, M. Wilkinson, S. P. Loader, S. D. Biju, K. Guillaume, L. Moriau, F. Bossuyt. 2007. “Global patterns of diversification in the history of modern amphibians.” *Proceedings of the National Academy of Sciences of the United States of America* 104, no. 3: 887–892. <https://doi.org/10.1073/pnas.0608378104>.

Zhang, P., D. Liang, R. L. Mao, D. M. Hillis, D. B. Wake, D. C. Cannatella. 2013. “Efficient sequencing of Anuran mtDNAs and a mitogenomic exploration of the phylogeny and evolution of frogs.” *Molecular Biology and Evolution* 30, no. 8: 1899–1915. <https://doi.org/10.1093/molbev/mst091>.

Zhang, J. F., G. P. Miao, S. J. Hu, Q. Sun, H. W. Ding, Z. C Ji, P. Guo, S. B. Yan, C. R. Wang, X. Z. Kan, L. W. Nie. 2021. “Quantification and evolution of mitochondrial genome rearrangement in Amphibians.” *BMC ecology and evolution* 21, no. 1: 19. <https://doi.org/10.1186/s12862-021-01755-3>.
