# Supplementary material for: Mitochondrial Phylogenomics and Genome Evolution in Anura: Insights From Structure and Gene Order Rearrangements
Source: Ecol Evol. 2026 Mar 30;16(4):e73370. doi: 10.1002/ece3.73370 (PMC13107284; doi:10.1002/ece3.73370)
Supplement: Supplementary file 22 — Table S1: List of taxonomic samples and sequences used in this study. [file ECE3-16-e73370-s004.docx]

| **Order** | **Superfamily** | **Family** | **Subfamily** | **Genus** | **Species** | **GenBank No.** |
| --- | --- | --- | --- | --- | --- | --- |
| Anura |  | Alytidae |  | *Alytes* | *Alytes obstetricans* | NC_006688.1 |
| Anura |  | Alytidae |  | *Discoglossus* | *Discoglossus galganoi* | NC_006690.1 |
| Anura |  | Alytidae |  | *Discoglossus* | *Discoglossus pictus* | CM050223.1 |
| Anura |  | Arthroleptidae | Astylosterninae | *Astylosternus* | *Astylosternus robustus* | NC_023382.1 |
| Anura |  | Bombinatoridae |  | *Bombina* | *Bombina bombina* | NC_042501.1 |
| Anura |  | Bombinatoridae |  | *Bombina* | *Bombina fortinuptialis* | NC_006402.1 |
| Anura |  | Bombinatoridae |  | *Bombina* | *Bombina lichuanensis* | NC_021477.1 |
| Anura |  | Bombinatoridae |  | *Bombina* | *Bombina maxima* | NC_011049.1 |
| Anura |  | Bombinatoridae |  | *Bombina* | *Bombina microdeladigitora* | NC_021476.1 |
| Anura |  | Bombinatoridae |  | *Bombina* | *Bombina orientalis* | NC_006689.1 |
| Anura |  | Bombinatoridae |  | *Bombina* | *Bombina variegata* | NC_009258.1 |
| Anura | Brachycephaloidea | Brachycephalidae |  | *Ischnocnema* | *Ischnocnema guentheri* | MH492737.1 |
| Anura | Brachycephaloidea | Brachycephalidae |  | *Ischnocnema* | *Ischnocnema henselii* | MH492733.1 |
| Anura | Brachycephaloidea | Brachycephalidae |  | *Ischnocnema* | *Ischnocnema nasuta* | MH492734.1 |
| Anura | Brachycephaloidea | Eleutherodactylidae | Eleutherodactylinae | *Eleutherodactylus* | *Eleutherodactylus coqui* | CM069431.1 |
| Anura |  | Brevicipitidae |  | *Breviceps* | *Breviceps adspersus* | NC_023379.1 |
| Anura |  | Brevicipitidae |  | *Breviceps* | *Breviceps mossambicus* | LC498571.1 |
| Anura |  | Brevicipitidae |  | *Breviceps* | *Breviceps poweri* | LC498572.1 |
| Anura |  | Bufonidae |  | *Anaxyrus* | *Anaxyrus americanus* | NC_047224.1 |
| Anura |  | Bufonidae |  | *Bufo* | *Bufo bufo* | LR991678.1 |
| Anura |  | Bufonidae |  | *Bufo* | *Bufo gargarizans* | NC_008410.1 |
| Anura |  | Bufonidae |  | *Bufo* | *Bufo minshanicus* | KM587710.1 |
| Anura |  | Bufonidae |  | *Bufo* | *Bufo praetextatus* | NC_009886.1 |
| Anura |  | Bufonidae |  | *Bufo* | *Bufo stejnegeri* | NC_027686.1 |
| Anura |  | Bufonidae |  | *Bufo* | *Bufo tibetanus* | NC_020048.1 |
| Anura |  | Bufonidae |  | *Bufotes* | *Bufotes pewzowi* | NC_047225.1 |
| Anura |  | Bufonidae |  | *Bufotes* | *Bufotes pseudoraddei* | NC_046047.1 |
| Anura |  | Bufonidae |  | *Bufotes* | *Bufotes viridis* | NC_050665.1 |
| Anura |  | Bufonidae |  | *Bufotes* | *Bufotes turanensis* | NC_062077.1 |
| Anura |  | Bufonidae |  | *Duttaphrynus* | *Duttaphrynus melanostictus* | NC_005794.2 |
| Anura |  | Bufonidae |  | *Melanophryniscus* | *Melanophryniscus moreirae* | NC_037378.1 |
| Anura |  | Bufonidae |  | *Rhinella* | *Rhinella marina* | NC_066225.1 |
| Anura |  | Bufonidae |  | *Strauchbufo* | *Strauchbufo raddei* | NC_028424.1 |
| Anura |  | Calyptocephalellidae |  | *Calyptocephalella* | *Calyptocephalella gayi* | JF703228.1 |
| Anura |  | Calyptocephalellidae |  | *Telmatobufo* | *Telmatobufo australis* | KR349313.1 |
| Anura |  | Ceratobatrachidae | Ceratobatrachinae | *Cornufer* | *Cornufer vitianus* | NC_027671.1 |
| Anura | Dendrobatoidea | Aromobatidae | Anomaloglossinae | *Anomaloglossus* | *Anomaloglossus baeobatrachus* | NC_030054.1 |
| Anura | Dendrobatoidea | Aromobatidae | Anomaloglossinae | *Anomaloglossus* | *Anomaloglossus blanci* | NC_037857.1 |
| Anura | Dendrobatoidea | Aromobatidae | Anomaloglossinae | *Anomaloglossus* | *Anomaloglossus degranvillei* | NC_037856.1 |
| Anura | Dendrobatoidea | Aromobatidae | Anomaloglossinae | *Anomaloglossus* | *Anomaloglossus dewynteri* | NC_037855.1 |
| Anura | Dendrobatoidea | Aromobatidae | Anomaloglossinae | *Anomaloglossus* | *Anomaloglossus surinamensis* | NC_037854.1 |
| Anura | Dendrobatoidea | Dendrobatidae | Dendrobatinae | *Phyllobates* | *Phyllobates terribilis* | NC_037380.1 |
| Anura | Dendrobatoidea | Dendrobatidae | Dendrobatinae | *Ranitomeya* | *Ranitomeya imitator* | CM064416.1 |
| Anura | Dendrobatoidea | Dendrobatidae | Hyloxalinae | *Hyloxalus* | *Hyloxalus subpunctatus* | NC_037379.1 |
| Anura |  | Dicroglossidae | Dicroglossinae | *Euphlyctis* | *Euphlyctis hexadactyla* | NC_014584.1 |
| Anura |  | Dicroglossidae | Dicroglossinae | *Fejervarya* | *Fejervarya cancrivora* | NC_012647.1 |
| Anura |  | Dicroglossidae | Dicroglossinae | *Fejervarya* | *Fejervarya limnocharis* | NC_005055.1 |
| Anura |  | Dicroglossidae | Dicroglossinae | *Fejervarya* | *Fejervarya multistriata* | NC_029754.1 |
| Anura |  | Dicroglossidae | Dicroglossinae | *Hoplobatrachus* | *Hoplobatrachus chinensis* | NC_042258.1 |
| Anura |  | Dicroglossidae | Dicroglossinae | *Hoplobatrachus* | *Hoplobatrachus tigerinus* | NC_014581.1 |
| Anura |  | Dicroglossidae | Dicroglossinae | *Limnonectes* | *Limnonectes bannaensis* | AY899242.1 |
| Anura |  | Dicroglossidae | Dicroglossinae | *Limnonectes* | *Limnonectes blythii* | NC_068685.1 |
| Anura |  | Dicroglossidae | Dicroglossinae | *Limnonectes* | *Limnonectes fragilis* | AY899241.1 |
| Anura |  | Dicroglossidae | Dicroglossinae | *Limnonectes* | *Limnonectes fujianensis* | NC_007440.2 |
| Anura |  | Dicroglossidae | Dicroglossinae | *Minervarya* | *Minervarya manoharani* | KY463521.1 |
| Anura |  | [Dicroglossidae](https://amphibiansoftheworld.amnh.org/Amphibia/Anura/Dicroglossidae) | Dicroglossinae | *Nanorana* | *Nanorana kangxianensis* | MZ895123.1 |
| Anura |  | [Dicroglossidae](https://amphibiansoftheworld.amnh.org/Amphibia/Anura/Dicroglossidae) | Dicroglossinae | *Nanorana* | *Nanorana parkeri* | NC_026789.1 |
| Anura |  | [Dicroglossidae](https://amphibiansoftheworld.amnh.org/Amphibia/Anura/Dicroglossidae) | Dicroglossinae | *Nanorana* | *Nanorana pleskei* | NC_016119.1 |
| Anura |  | [Dicroglossidae](https://amphibiansoftheworld.amnh.org/Amphibia/Anura/Dicroglossidae) | Dicroglossinae | *Nanorana* | *Nanorana taihangnica* | NC_024272.1 |
| Anura |  | [Dicroglossidae](https://amphibiansoftheworld.amnh.org/Amphibia/Anura/Dicroglossidae) | Dicroglossinae | *Nanorana* | *Nanorana ventripunctata* | NC_039094.1 |
| Anura |  | Dicroglossidae | Dicroglossinae | *Quasipaa* | *Quasipaa boulengeri* | NC_021937.1 |
| Anura |  | Dicroglossidae | Dicroglossinae | *Quasipaa* | *Quasipaa exilispinosa* | NC_056269.1 |
| Anura |  | Dicroglossidae | Dicroglossinae | *Quasipaa* | *Quasipaa robertingeri* | KY441640.1 |
| Anura |  | Dicroglossidae | Dicroglossinae | *Quasipaa* | *Quasipaa spinosa* | NC_013270.1 |
| Anura |  | Dicroglossidae | Dicroglossinae | *Quasipaa* | *Quasipaa yei* | NC_024843.1 |
| Anura |  | [Dicroglossidae](https://amphibiansoftheworld.amnh.org/Amphibia/Anura/Dicroglossidae) | Occidozyginae | *Occidozyga* | *Occidozyga martensii* | NC_014685.1 |
| Anura |  | Dicroglossidae | Occidozyginae | *Occidozyga* | *Occidozyga myanhessei* | NC_057992.1 |
| Anura |  | Heleophrynidae |  | *Heleophryne* | *Heleophryne regis* | NC_019998.1 |
| Anura |  | Hemisotidae |  | *Hemisus* | *Hemisus marmoratus* | NC_023380.1 |
| Anura |  | Hylidae | Hylinae | *Bokermannohyla* | *Bokermannohyla alvarengai* | NC_036493.1 |
| Anura |  | Hylidae | Hylinae | *Dendropsophus* | *Dendropsophus ebraccatus* | CM051072.1 |
| Anura |  | Hylidae | Hylinae | *Dryophytes* | *Dryophytes andersonii* | NC_063648.1 |
| Anura |  | Hylidae | Hylinae | *Dryophytes* | *Dryophytes femoralis* | NC_063649.1 |
| Anura |  | Hylidae | Hylinae | *Dryophytes* | *Dryophytes japonicus* | NC_010232.1 |
| Anura |  | Hylidae | Hylinae | *Dryophytes* | *Dryophytes suweonensis* | NC_032380.1 |
| Anura |  | Hylidae | Hylinae | *Dryophytes* | *Dryophytes versicolor* | NC_045917.1 |
| Anura |  | Hylidae | Hylinae | *Hyla* | *Hyla annectans* | NC_025309.1 |
| Anura |  | Hylidae | Hylinae | *Hyla* | *Hyla chinensis* | NC_006403.1 |
| Anura |  | Hylidae | Hylinae | *Hyla* | *Hyla sanchiangensis* | NC_062326.1 |
| Anura |  | Hylidae | Hylinae | *Hyla* | *Hyla sarda* | CM056048.1 |
| Anura |  | Hylidae | Hylinae | *Hyla* | *Hyla tsinlingensis* | NC_026524.1 |
| Anura |  | Hylidae | Hylinae | *Pseudis* | *Pseudis tocantins* | NC_041426.1 |
| Anura |  | Hylidae | Phyllomedusinae | *Phyllomedusa* | *Phyllomedusa bahiana* | NC_067554.1 |
| Anura |  | Hylidae | Phyllomedusinae | *Pithecopus* | *Pithecopus megacephalus* | MG772558.1 |
| Anura |  | Hyperoliidae | Hyperoliinae | *Hyperolius* | *Hyperolius marmoratus* | NC_023381.1 |
| Anura |  | Leiopelmatidae |  | *Leiopelma* | *Leiopelma archeyi* | NC_014691.1 |
| Anura |  | Leiopelmatidae |  | *Leiopelma* | *Leiopelma hochstetteri* | NC_027072.1 |
| Anura |  | Leptodactylidae | Leptodactylinae | *Leptodactylus* | *Leptodactylus fallax* | NC_056366.1 |
| Anura |  | Leptodactylidae | Leptodactylinae | *Leptodactylus* | *Leptodactylus fuscus* | CM063215.1 |
| Anura |  | Mantellidae | Mantellinae | *Mantella* | *Mantella baroni* | NC_039758.1 |
| Anura |  | Mantellidae | Mantellinae | *Mantella* | *Mantella madagascariensis* | NC_007888.1 |
| Anura |  | Megophryidae | Leptobrachiinae | *Leptobrachella* | *Leptobrachella alpina* | MW487804.1 |
| Anura |  | Megophryidae | Leptobrachiinae | *Leptobrachella* | *Leptobrachella oshanensis* | NC_020610.1 |
| Anura |  | Megophryidae | Leptobrachiinae | *Leptobrachium* | *Leptobrachium ailaonicum* | MZ394043.1 |
| Anura |  | Megophryidae | Leptobrachiinae | *Leptobrachium* | *Leptobrachium boringii* | NC_024427.1 |
| Anura |  | Megophryidae | Leptobrachiinae | *Leptobrachium* | *Leptobrachium leishanense* | NC_031411.1 |
| Anura |  | Megophryidae | Leptobrachiinae | *Leptobrachium* | *Leptobrachium liui* | NC_057468.1 |
| Anura |  | Megophryidae | Leptobrachiinae | *Oreolalax* | *Oreolalax jingdongensis* | MF953479.1 |
| Anura |  | Megophryidae | Leptobrachiinae | *Oreolalax* | *Oreolalax lichuanensis* | NC_030627.1 |
| Anura |  | Megophryidae | Leptobrachiinae | *Oreolalax* | *Oreolalax major* | NC_030605.1 |
| Anura |  | Megophryidae | Leptobrachiinae | *Oreolalax* | *Oreolalax multipunctatus* | NC_037382.1 |
| Anura |  | Megophryidae | Leptobrachiinae | *Oreolalax* | *Oreolalax omeimontis* | NC_049862.1 |
| Anura |  | Megophryidae | Leptobrachiinae | *Oreolalax* | *Oreolalax rhodostigmatus* | MF770485.1 |
| Anura |  | Megophryidae | Leptobrachiinae | *Oreolalax* | *Oreolalax schmidti* | NC_056343.1 |
| Anura |  | Megophryidae | Leptobrachiinae | *Oreolalax* | *Oreolalax xiangchengensis* | MH727696.1 |
| Anura |  | Megophryidae | Leptobrachiinae | *Scutiger* | *Scutiger ningshanensis* | NC_031426.1 |
| Anura |  | Megophryidae | Megophryinae | *Atympanophrys* | *Atympanophrys gigantica* | MZ364157.1 |
| Anura |  | Megophryidae | Megophryinae | *Atympanophrys* | *Atympanophrys shapingensis* | NC_018785.1 |
| Anura |  | Megophryidae | Megophryinae | *Boulenophrys* | *Boulenophrys baishanzuensis* | NC_081000.1 |
| Anura |  | Megophryidae | Megophryinae | *Boulenophrys* | *Boulenophrys boettgeri* | OR529440.1 |
| Anura |  | Megophryidae | Megophryinae | *Boulenophrys* | *Boulenophrys kuatunensis* | OR522721.1 |
| Anura |  | Megophryidae | Megophryinae | *Boulenophrys* | *Boulenophrys spinata* | ON646614.1 |
| Anura |  | Microhylidae | Cophylinae | *Anilany* | *Anilany helenae* | MZ751042.1 |
| Anura |  | Microhylidae | Microhylinae | *Glyphoglossus* | *Glyphoglossus yunnanensis* | MZ542769.1 |
| Anura |  | Microhylidae | Kalophryninae | *Kalophrynus* | *Kalophrynus palmatissimus* | NC_068684.1 |
| Anura |  | Microhylidae | Microhylinae | *Kaloula* | *Kaloula borealis* | NC_020044.1 |
| Anura |  | Microhylidae | Microhylinae | *Kaloula* | *Kaloula pulchra* | NC_006405.1 |
| Anura |  | Microhylidae | Microhylinae | *Kaloula* | *Kaloula rugifera* | NC_029409.1 |
| Anura |  | Microhylidae | Microhylinae | *Kaloula* | *Kaloula verrucosa* | NC_039411.1 |
| Anura |  | Microhylidae | Microhylinae | *Microhyla* | *Microhyla achatina* | MW233587.1 |
| Anura |  | Microhylidae | Microhylinae | *Microhyla* | *Microhyla beilunensis* | MT559308.1 |
| Anura |  | Microhylidae | Microhylinae | *Microhyla* | *Microhyla butleri* | NC_030049.1 |
| Anura |  | Microhylidae | Microhylinae | *Microhyla* | *Microhyla fissipes* | NC_045110.1 |
| Anura |  | Microhylidae | Microhylinae | *Microhyla* | *Microhyla heymonsi* | NC_006406.1 |
| Anura |  | Microhylidae | Microhylinae | *Microhyla* | *Microhyla mixtura* | NC_038130.1 |
| Anura |  | Microhylidae | Microhylinae | *Microhyla* | *Microhyla okinavensis* | NC_010233.1 |
| Anura |  | Microhylidae | Microhylinae | *Microhyla* | *Microhyla ornata* | NC_009422.1 |
| Anura |  | Microhylidae | Microhylinae | *Microhyla* | *Microhyla pulchra* | NC_024547.1 |
| Anura |  | Microhylidae | Microhylinae | *Microhyla* | *Microhyla taraiensis* | NC_039176.1 |
| Anura | Myobatrachoidea | Limnodynastidae |  | *Platyplectrum* | *Platyplectrum melanopyga* | NC_019999.1 |
| Anura | Myobatrachoidea | Myobatrachidae |  | *Mixophyes* | *Mixophyes australis* | CM070084.1 |
| Anura | Myobatrachoidea | Myobatrachidae |  | *Taudactylus* | *Taudactylus pleione* | CM062684.1 |
| Anura |  | Pelobatidae |  | *Pelobates* | *Pelobates cultripes* | NC_008144.1 |
| Anura |  | Pelobatidae |  | *Pelobates* | *Pelobates fuscus* | NC_051953.1 |
| Anura |  | Pelodytidae |  | *Pelodytes* | *Pelodytes ibericus* | CM069894.1 |
| Anura |  | Pelodytidae |  | *Pelodytes* | *Pelodytes punctatus* | NC_020000.1 |
| Anura |  | Pipidae | Dactylethrinae | *Hymenochirus* | *Hymenochirus boettgeri* | NC_015615.1 |
| Anura |  | Pipidae | Dactylethrinae | *Pseudhymenochirus* | *Pseudhymenochirus merlini* | NC_015618.1 |
| Anura |  | Pipidae | Dactylethrinae | *Xenopus* | *Xenopus allofraseri* | NC_044874.1 |
| Anura |  | Pipidae | Dactylethrinae | *Xenopus* | *Xenopus amieti* | NC_044876.1 |
| Anura |  | Pipidae | Dactylethrinae | *Xenopus* | *Xenopus andrei* | NC_044878.1 |
| Anura |  | Pipidae | Dactylethrinae | *Xenopus* | *Xenopus borealis* | NC_018776.1 |
| Anura |  | Pipidae | Dactylethrinae | *Xenopus* | *Xenopus boumbaensis* | NC_044877.1 |
| Anura |  | Pipidae | Dactylethrinae | *Xenopus* | *Xenopus calcaratus* | NC_044865.1 |
| Anura |  | Pipidae | Dactylethrinae | *Xenopus* | *Xenopus clivii* | NC_044886.1 |
| Anura |  | Pipidae | Dactylethrinae | *Xenopus* | *Xenopus epitropicalis* | NC_044867.1 |
| Anura |  | Pipidae | Dactylethrinae | *Xenopus* | *Xenopus eysoole* | NC_044884.1 |
| Anura |  | Pipidae | Dactylethrinae | *Xenopus* | *Xenopus fischbergi* | NC_044888.1 |
| Anura |  | Pipidae | Dactylethrinae | *Xenopus* | *Xenopus gilli* | NC_044871.1 |
| Anura |  | Pipidae | Dactylethrinae | *Xenopus* | *Xenopus itombwensis* | NC_044879.1 |
| Anura |  | Pipidae | Dactylethrinae | *Xenopus* | *Xenopus kobeli* | NC_044883.1 |
| Anura |  | Pipidae | Dactylethrinae | *Xenopus* | *Xenopus laevis* | NC_001573.1 |
| Anura |  | Pipidae | Dactylethrinae | *Xenopus* | *Xenopus largeni* | NC_044868.1 |
| Anura |  | Pipidae | Dactylethrinae | *Xenopus* | *Xenopus lenduensis* | NC_044880.1 |
| Anura |  | Pipidae | Dactylethrinae | *Xenopus* | *Xenopus longipes* | NC_044885.1 |
| Anura |  | Pipidae | Dactylethrinae | *Xenopus* | *Xenopus mellotropicalis* | NC_044866.1 |
| Anura |  | Pipidae | Dactylethrinae | *Xenopus* | *Xenopus muelleri* | NC_044887.1 |
| Anura |  | Pipidae | Dactylethrinae | *Xenopus* | *Xenopus parafraseri* | NC_044872.1 |
| Anura |  | Pipidae | Dactylethrinae | *Xenopus* | *Xenopus petersii* | NC_044869.1 |
| Anura |  | Pipidae | Dactylethrinae | *Xenopus* | *Xenopus poweri* | NC_044870.1 |
| Anura |  | Pipidae | Dactylethrinae | *Xenopus* | *Xenopus pygmaeus* | NC_044873.1 |
| Anura |  | Pipidae | Dactylethrinae | *Xenopus* | *Xenopus ruwenzoriensis* | NC_044882.1 |
| Anura |  | Pipidae | Dactylethrinae | *Xenopus* | *Xenopus tropicalis* | NC_006839.1 |
| Anura |  | Pipidae | Dactylethrinae | *Xenopus* | *Xenopus vestitus* | NC_044881.1 |
| Anura |  | Pipidae | Dactylethrinae | *Xenopus* | *Xenopus victorianus* | NC_018775.1 |
| Anura |  | Pipidae | Dactylethrinae | *Xenopus* | *Xenopus wittei* | NC_044875.1 |
| Anura |  | Pipidae | Pipinae | *Pipa* | *Pipa carvalhoi* | NC_015617.1 |
| Anura |  | Pipidae | Pipinae | *Pipa* | *Pipa myersi* | NC_061926.1 |
| Anura |  | Pipidae | Pipinae | *Pipa* | *Pipa pipa* | NC_061389.1 |
| Anura |  | Pipidae | Pipinae | *Pipa* | *Pipa snethlageae* | NC_061925.1 |
| Anura |  | Pyxicephalidae | Pyxicephalinae | *Pyxicephalus* | *Pyxicephalus adspersus* | NC_044480.1 |
| Anura |  | Ptychadenidae |  | *Ptychadena* | *Ptychadena amharensis* | NC_082083.1 |
| Anura |  | Ptychadenidae |  | *Ptychadena* | *Ptychadena anchietae* | NC_082080.1 |
| Anura |  | Ptychadenidae |  | *Ptychadena* | *Ptychadena beka* | NC_082088.1 |
| Anura |  | Ptychadenidae |  | *Ptychadena* | *Ptychadena cooperi* | NC_082084.1 |
| Anura |  | Ptychadenidae |  | *Ptychadena* | *Ptychadena delphina* | NC_082091.1 |
| Anura |  | Ptychadenidae |  | *Ptychadena* | *Ptychadena doro* | NC_082090.1 |
| Anura |  | Ptychadenidae |  | *Ptychadena* | *Ptychadena erlangeri* | NC_082087.1 |
| Anura |  | Ptychadenidae |  | *Ptychadena* | *Ptychadena goweri* | NC_082089.1 |
| Anura |  | Ptychadenidae |  | *Ptychadena* | *Ptychadena harenna* | NC_082093.1 |
| Anura |  | Ptychadenidae |  | *Ptychadena* | *Ptychadena levenorum* | NC_082085.1 |
| Anura |  | Ptychadenidae |  | *Ptychadena* | *Ptychadena nana* | NC_082086.1 |
| Anura |  | Ptychadenidae |  | *Ptychadena* | *Ptychadena neumanni* | NC_082092.1 |
| Anura |  | Ptychadenidae |  | *Ptychadena* | *Ptychadena nuerensis* | NC_082081.1 |
| Anura |  | Ptychadenidae |  | *Ptychadena* | *Ptychadena robeensis* | NC_082094.1 |
| Anura |  | Ptychadenidae |  | *Ptychadena* | *Ptychadena wadei* | NC_082082.1 |
| Anura |  | Ranidae |  | *Amolops* | *Amolops granulosus* | NC_044901.1 |
| Anura |  | Ranidae |  | *Amolops* | *Amolops jinjiangensis* | MZ292455.1 |
| Anura |  | Ranidae |  | *Amolops* | *Amolops loloensis* | NC_029250.1 |
| Anura |  | Ranidae |  | *Amolops* | *Amolops mantzorum* | NC_024180.1 |
| Anura |  | Ranidae |  | *Amolops* | *Amolops ricketti* | NC_023949.1 |
| Anura |  | Ranidae |  | *Amolops* | *Amolops sinensis* | ON646615.1 |
| Anura |  | Ranidae |  | *Amolops* | *Amolops wuyiensis* | NC_025591.1 |
| Anura |  | Ranidae |  | *Amerana* | *Amerana draytonii* | NC_028296.1 |
| Anura |  | Ranidae |  | *Aquarana* | *Aquarana catesbeiana*_1 | NC_022696.1 |
| Anura |  | Ranidae |  | *Aquarana* | *Aquarana catesbeiana*_2 | NC_068690.1 |
| Anura |  | Ranidae |  | *Aquarana* | *Aquarana clamitans* | CM075516.1 |
| Anura |  | Ranidae |  | *Aquarana* | *Aquarana okaloosae* | NC_028283.1 |
| Anura |  | Ranidae |  | *Babina* | *Babina holsti* | NC_022870.1 |
| Anura |  | Ranidae |  | *Babina* | *Babina subaspera* | NC_022871.1 |
| Anura |  | Ranidae |  | *Boreorana* | *Boreorana sylvatica* | NC_027236.1 |
| Anura |  | Ranidae |  | *Glandirana* | *Glandirana emeljanovi* | NC_030211.1 |
| Anura |  | Ranidae |  | *Glandirana* | *Glandirana rugosa* | LC536282.1 |
| Anura |  | Ranidae |  | *Glandirana* | *Glandirana tientaiensis* | NC_025226.1 |
| Anura |  | Ranidae |  | *Hylarana* | *Hylarana guentheri* | NC_024748.1 |
| Anura |  | Ranidae |  | *Hylarana* | *Hylarana labialis* | NC_068686.1 |
| Anura |  | Ranidae |  | *Hylarana* | *Hylarana latouchii* | NC_057198.1 |
| Anura |  | Ranidae |  | *Nidirana* | *Nidirana adenopleura* | NC_018771.1 |
| Anura |  | Ranidae |  | *Nidirana* | *Nidirana daunchina* | OR528757.1 |
| Anura |  | Ranidae |  | *Nidirana* | *Nidirana okinavana* | NC_022872.1 |
| Anura |  | Ranidae |  | *Nidirana* | *Nidirana yeae* | MW411575.1 |
| Anura |  | Ranidae |  | *Odorrana* | *Odorrana exiliversabilis* | NC_053712.1 |
| Anura |  | Ranidae |  | *Odorrana* | *Odorrana grahami* | NC_059861.1 |
| Anura |  | Ranidae |  | *Odorrana* | *Odorrana graminea* | NC_050884.1 |
| Anura |  | Ranidae |  | *Odorrana* | *Odorrana hainanensis* | NC_034984.1 |
| Anura |  | Ranidae |  | *Odorrana* | *Odorrana hejiangensis* | MZ895124.1 |
| Anura |  | Ranidae |  | *Odorrana* | *Odorrana hosii* | NC_068691.1 |
| Anura |  | Ranidae |  | *Odorrana* | *Odorrana ishikawae* | NC_015305.1 |
| Anura |  | Ranidae |  | *Odorrana* | *Odorrana jingdongensis* | NC_065297.1 |
| Anura |  | Ranidae |  | *Odorrana* | *Odorrana livida* | NC_043768.1 |
| Anura |  | Ranidae |  | *Odorrana* | *Odorrana macrotympana* | MW551526.1 |
| Anura |  | Ranidae |  | *Odorrana* | *Odorrana margaretae* | NC_024603.1 |
| Anura |  | Ranidae |  | *Odorrana* | *Odorrana nasuta* | NC_082181.1 |
| Anura |  | Ranidae |  | *Odorrana* | *Odorrana schmackeri* | NC_027827.1 |
| Anura |  | Ranidae |  | *Odorrana* | *Odorrana tormota* | NC_009423.1 |
| Anura |  | Ranidae |  | *Odorrana* | *Odorrana wuchuanensis* | NC_034983.1 |
| Anura |  | Ranidae |  | *Pelophylax* | *Pelophylax cerigensis* | OQ694802.1 |
| Anura |  | Ranidae |  | *Pelophylax* | *Pelophylax cretensis* | NC_025575.1 |
| Anura |  | Ranidae |  | *Pelophylax* | *Pelophylax cypriensis* | NC_026893.1 |
| Anura |  | Ranidae |  | *Pelophylax* | *Pelophylax epeiroticus* | NC_026894.1 |
| Anura |  | Ranidae |  | *Pelophylax* | *Pelophylax kurtmuelleri* | NC_026895.1 |
| Anura |  | Ranidae |  | *Pelophylax* | *Pelophylax lessonae* | NC_029200.1 |
| Anura |  | Ranidae |  | *Pelophylax* | *Pelophylax nigromaculatus* | NC_002805.1 |
| Anura |  | Ranidae |  | *Pelophylax* | *Pelophylax plancyi* | NC_009264.1 |
| Anura |  | Ranidae |  | *Pelophylax* | *Pelophylax ridibundus* | NC_029199.1 |
| Anura |  | Ranidae |  | *Pelophylax* | *Pelophylax shqipericus* | NC_026896.1 |
| Anura |  | Ranidae |  | *Rana* | *Rana amurensis* | NC_030042.1 |
| Anura |  | Ranidae |  | *Rana* | *Rana chensinensis* | NC_023529.1 |
| Anura |  | Ranidae |  | *Rana* | *Rana chaochiaoensis* | NC_035803.1 |
| Anura |  | Ranidae |  | *Rana* | *Rana coreana* | NC_068259.1 |
| Anura |  | Ranidae |  | *Rana* | *Rana dabieshanensis* | NC_060306.1 |
| Anura |  | Ranidae |  | *Rana* | *Rana dybowskii* | NC_023528.1 |
| Anura |  | Ranidae |  | *Rana* | *Rana hanluica* | NC_061371.1 |
| Anura |  | Ranidae |  | *Rana* | *Rana huanrensis* | NC_028521.1 |
| Anura |  | Ranidae |  | *Rana* | *Rana johnsi* | NC_058599.1 |
| Anura |  | Ranidae |  | *Rana* | *Rana kukunoris* | NC_035804.1 |
| Anura |  | Ranidae |  | *Rana* | *Rana longicrus* | NC_061370.1 |
| Anura |  | Ranidae |  | *Rana* | *Rana omeimontis* | NC_035805.1 |
| Anura |  | Ranidae |  | *Rana* | *Rana pyrenaica* | KU720300.1 |
| Anura |  | Ranidae |  | *Rana* | *Rana sangzhiensis* | MT782121.1 |
| Anura |  | Ranidae |  | *Rana* | *Rana temporaria* | NC_042226.1 |
| Anura |  | Ranidae |  | *Rana* | *Rana uenoi* | NC_056272.1 |
| Anura |  | Ranidae |  | *Rana* | *Rana wuyiensis* | OL467321.1 |
| Anura |  | Ranidae |  | *Rana* | *Rana zhenhaiensis* | NC_069580.1 |
| Anura |  | Ranixalidae |  | *Indirana* | *Indirana semipalmdata* | KX774468.1 |
| Anura |  | Rhacophoridae | Buergeriinae | *Buergeria* | *Buergeria buergeri* | AB127977.1 |
| Anura |  | Rhacophoridae | Buergeriinae | *Buergeria* | *Buergeria japonica* | LC739528.1 |
| Anura |  | Rhacophoridae | Rhacophorinae | *Gracixalus* | *Gracixalus yunnanensis* | NC_061400.1 |
| Anura |  | Rhacophoridae | Rhacophorinae | *Polypedates* | *Polypedates braueri* | NC_042797.1 |
| Anura |  | Rhacophoridae | Rhacophorinae | *Polypedates* | *Polypedates impresus* | NC_062354.1 |
| Anura |  | Rhacophoridae | Rhacophorinae | *Polypedates* | *Polypedates leucomystax* | NC_062356.1 |
| Anura |  | Rhacophoridae | Rhacophorinae | *Polypedates* | *Polypedates megacephalus* | NC_043955.1 |
| Anura |  | Rhacophoridae | Rhacophorinae | *Polypedates* | *Polypedates mutus* | NC_062355.1 |
| Anura |  | Rhacophoridae | Rhacophorinae | *Rhacophorus* | *Rhacophorus rhodopus* | OK181853.1 |
| Anura |  | Rhacophoridae | Rhacophorinae | *Zhangixalus* | *Zhangixalus arboreus* | LC565708.1 |
| Anura |  | Rhacophoridae | Rhacophorinae | *Zhangixalus* | *Zhangixalus burmanus* | OR161035.1 |
| Anura |  | Rhacophoridae | Rhacophorinae | *Zhangixalus* | *Zhangixalus chenfui* | NC_062878.1 |
| Anura |  | Rhacophoridae | Rhacophorinae | *Zhangixalus* | *Zhangixalus dennysi* | NC_027452.1 |
| Anura |  | Rhacophoridae | Rhacophorinae | *Zhangixalus* | *Zhangixalus dugritei* | MZ712011.1 |
| Anura |  | Rhacophoridae | Rhacophorinae | *Zhangixalus* | *Zhangixalus omeimontis* | NC_046387.1 |
| Anura |  | Rhacophoridae | Rhacophorinae | *Zhangixalus* | *Zhangixalus schlegelii* | NC_007178.1 |
| Anura |  | Rhinophrynidae |  | *Rhinophrynus* | *Rhinophrynus dorsalis* | NC_015620.1 |
| Anura |  | Scaphiopodidae |  | *Scaphiopus* | *Scaphiopus holbrookii* | NC_037377.1 |
| Anura |  | Scaphiopodidae |  | *Spea* | *Spea hammondii* | CM055131.1 |
| Anura |  | Sooglossidae |  | *Sooglossus* | *Sooglossus thomasseti* | NC_020001.1 |
| Anura |  | Telmatobiidae |  | *Telmatobius* | *Telmatobius bolivianus* | NC_020002.1 |
| Anura |  | Telmatobiidae |  | *Telmatobius* | *Telmatobius chusmisensis* | NC_030333.1 |
| Caudata |  | Hynobiidae | Hynobiinae | *Batrachuperus* | *Batrachuperus londongensis* | NC_008077.1 |
| Gymnophiona |  | Dermophiidae |  | *Gymnopis* | *Gymnopis multiplicata* | NC_020139.1 |
