# Supplementary material for: Mitochondrial Phylogenomics and Genome Evolution in Anura: Insights From Structure and Gene Order Rearrangements
Source: Ecol Evol. 2026 Mar 30;16(4):e73370. doi: 10.1002/ece3.73370 (PMC13107284; doi:10.1002/ece3.73370)
Supplement: Supplementary file 23 — Table S2: Best partitioning schemes and substitution models selected by ModelFinder. [file ECE3-16-e73370-s003.docx]

| Data Matrix | Subset | Best Model | # sites | Partition names |
| --- | --- | --- | --- | --- |
| 24NT-ML | 1 | GTR+F+R5 | 145 | *trnL1*, *trnI* |
|  | 2 | TIM2+F+I+I+R7 | 174 | *nad6*_codon1 |
|  | 3 | GTR+F+I+I+R5 | 174 | *nad6*_codon2 |
|  | 4 | TIM2+F+I+I+R5 | 174 | *nad6*_codon3 |
|  | 5 | GTR+F+I+I+R6 | 384 | *cytb*_codon1 |
|  | 6 | GTR+F+I+I+R4 | 384 | *cytb*_codon2 |
|  | 7 | TIM3+F+R10 | 457 | *cytb*_codon3, *trnV* |
|  | 8 | GTR+F+I+I+R7 | 1029 | *rrnS* |
|  | 9 | GTR+F+I+I+R8 | 1728 | *rrnL* |
|  | 10 | GTR+F+I+I+R5 | 146 | *trnL2*, *trnR* |
|  | 11 | GTR+F+I+I+R7 | 328 | *nad1*_codon1 |
|  | 12 | TVM+F+I+I+R5 | 328 | *nad1*_codon2 |
|  | 13 | GTR+F+ASC+R8 | 328 | *nad1*_codon3 |
|  | 14 | TVM+F+I+I+R4 | 71 | *trnM* |
|  | 15 | GTR+F+R8 | 348 | *nad2*_codon1 |
|  | 16 | GTR+F+I+I+R5 | 348 | *nad2*_codon2 |
|  | 17 | GTR+F+ASC+R7 | 348 | *nad2*_codon3 |
|  | 18 | SYM+I+I+R6 | 522 | *cox1*_codon1 |
|  | 19 | TVM+F+I+I+R4 | 522 | *cox1*_codon2 |
|  | 20 | TIM3+F+I+I+R7 | 522 | *cox1*_codon3 |
|  | 21 | TVM+F+R5 | 74 | *trnS2* |
|  | 22 | GTR+F+I+I+R5 | 70 | *trnD* |
|  | 23 | GTR+F+I+I+R5 | 229 | *cox2*_codon1 |
|  | 24 | TIM3+F+R4 | 229 | *cox2*_codon2 |
|  | 25 | TIM3+F+ASC+R6 | 229 | *cox2*_codon3 |
|  | 26 | GTR+F+I+I+R5 | 190 | *trnK*, *nad3*_codon1 |
|  | 27 | GTR+F+I+I+R5 | 231 | *atp6*_codon1 |
|  | 28 | TIM3+F+I+I+R5 | 231 | *atp6*_codon2 |
|  | 29 | TIM3+F+R8 | 231 | *atp6*_codon3 |
|  | 30 | SYM+I+I+R6 | 261 | *cox3*_codon1 |
|  | 31 | GTR+F+I+I+R5 | 261 | *cox3*_codon2 |
|  | 32 | TIM3+F+I+I+R7 | 261 | *cox3*_codon3 |
|  | 33 | TIM2+F+I+I+R5 | 73 | *trnG* |
|  | 34 | TVM+F+R5 | 114 | *nad3*_codon2 |
|  | 35 | TN+F+ASC+R6 | 114 | *nad3*_codon3 |
|  | 36 | GTR+F+R5 | 100 | *nad4L*_codon1 |
|  | 37 | GTR+F+R4 | 100 | *nad4L*_codon2 |
|  | 38 | TIM3+F+ASC+R5 | 100 | *nad4L*_codon3 |
|  | 39 | GTR+F+R7 | 459 | *nad4*_codon1 |
|  | 40 | GTR+F+I+I+R6 | 459 | *nad4*_codon2 |
|  | 41 | GTR+F+I+I+R6 | 459 | *nad4*_codon3 |
|  | 42 | SYM+R5 | 77 | *trnS1* |
| 24NT-BI | 1 | GTR+F+G4 | 234 | *trnL1*, *trnI*, *nad4L*_codon1 |
|  | 2 | GTR+F+I+G4 | 160 | *nad6*_codon1 |
|  | 3 | GTR+F+I+G4 | 150 | *nad6*_codon2 |
|  | 4 | GTR+F+I+G4 | 174 | *nad6*_codon3 |
|  | 5 | GTR+F+I+G4 | 636 | *cytb*_codon1, *trnM*, *cox2*_codon1, *cox3*_codon1 |
|  | 6 | GTR+F+I+G4 | 459 | *cytb*_codon2, *cox1*_codon2, *cox3*_codon2 |
|  | 7 | GTR+F+I+G4 | 524 | *cytb*_codon3, *trnV*, *trnD* |
|  | 8 | GTR+F+I+G4 | 837 | *rrnS* |
|  | 9 | GTR+F+I+G4 | 1358 | *rrnL* |
|  | 10 | GTR+F+I+G4 | 270 | *trnL2*, *trnG*, *trnR*, *trnS1* |
|  | 11 | GTR+F+I+G4 | 581 | *nad1*_codon1, *trnK*, *atp6*_codon1, *nad3*_codon1 |
|  | 12 | GTR+F+I+G4 | 187 | *nad1*_codon2 |
|  | 13 | GTR+F+G4 | 328 | *nad1*_codon3 |
|  | 14 | GTR+F+I+G4 | 708 | *nad2*_codon1, *nad4*_codon1 |
|  | 15 | GTR+F+I+G4 | 421 | *nad2*_codon2, *atp6*_codon2 |
|  | 16 | GTR+F+G4 | 348 | *nad2*_codon3 |
|  | 17 | SYM+I+G4 | 262 | *cox1*_codon1 |
|  | 18 | GTR+F+I+G4 | 521 | *cox1*_codon3 |
|  | 19 | GTR+F+G4 | 68 | *trnS2* |
|  | 20 | GTR+F+I+G4 | 124 | *cox2*_codon2 |
|  | 21 | GTR+F+ASC+G4 | 229 | *cox2*_codon3 |
|  | 22 | GTR+F+I+G4 | 344 | *atp6*_codon3, *nad3*_codon3 |
|  | 23 | GTR+F+I+G4 | 261 | *cox3*_codon3 |
|  | 24 | GTR+F+G4 | 79 | *nad3*_codon2 |
|  | 25 | GTR+F+G4 | 84 | *nad4L*_codon2 |
|  | 26 | GTR+F+G4 | 100 | *nad4L*_codon3 |
|  | 27 | GTR+F+I+G4 | 342 | *nad4*_codon2 |
|  | 28 | GTR+F+I+G4 | 459 | *nad4*_codon3 |
| 24NTS-ML | 1 | GTR+F+R5 | 73 | *trnL1* |
|  | 2 | TIM2+F+I+I+R7 | 174 | *nad6*_codonA |
|  | 3 | GTR+F+I+I+R5 | 174 | *nad6*_codonB |
|  | 4 | GTR+F+I+I+R6 | 384 | *cytb*_codonA |
|  | 5 | GTR+F+I+I+R4 | 384 | *cytb*_codonB |
|  | 6 | GTR+F+I+I+R7 | 1029 | *rrnS* |
|  | 7 | GTR+F+R5 | 73 | *trnV* |
|  | 8 | GTR+F+I+I+R8 | 1728 | *rrnL* |
|  | 9 | GTR+F+R5 | 76 | *trnL2* |
|  | 10 | GTR+F+I+I+R7 | 328 | *nad1*_codonA |
|  | 11 | TVM+F+I+I+R5 | 328 | *nad1*_codonB |
|  | 12 | GTR+F+I+I+R5 | 72 | *trnI* |
|  | 13 | TVM+F+I+I+R4 | 71 | *trnM* |
|  | 14 | GTR+F+R8 | 348 | *nad2*_codonA |
|  | 15 | GTR+F+R5 | 348 | *nad2*_codonB |
|  | 16 | SYM+I+I+R6 | 522 | *cox1*_codonA |
|  | 17 | TVM+F+I+I+R4 | 522 | *cox1*_codonB |
|  | 18 | TVM+F+R5 | 74 | *trnS2* |
|  | 19 | GTR+F+I+I+R5 | 70 | *trnD* |
|  | 20 | GTR+F+I+I+R5 | 229 | *cox2*_codonA |
|  | 21 | TIM3+F+R4 | 229 | *cox2*_codonB |
|  | 22 | GTR+F+I+I+R5 | 190 | *trnK*, *nad3*_codonA |
|  | 23 | GTR+F+I+I+R5 | 231 | *atp6*_codonA |
|  | 24 | TIM3+F+I+I+R5 | 231 | *atp6*_codonB |
|  | 25 | SYM+I+I+R6 | 261 | *cox3*_codonA |
|  | 26 | GTR+F+I+I+R4 | 261 | *cox3*_codonB |
|  | 27 | TIM2+F+I+I+R5 | 73 | *trnG* |
|  | 28 | TVM+F+R5 | 114 | *nad3*_codonB |
|  | 29 | TVM+F+I+I+R4 | 70 | *trnR* |
|  | 30 | GTR+F+R7 | 100 | *nad4L*_codonA |
|  | 31 | GTR+F+R5 | 559 | *nad4L*_codonB, *nad4*_codonB |
|  | 32 | GTR+F+R8 | 459 | *nad4*_codonA |
|  | 33 | SYM+R5 | 77 | *trnS1* |
| 24NTS-BI | 1 | GTR+F+I+G4 | 272 | *trnL1*, *trnL2*, *trnK*, *trnG* |
|  | 2 | GTR+F+I+G4 | 160 | *nad6*_codonA |
|  | 3 | GTR+F+I+G4 | 150 | *nad6*_codonB |
|  | 4 | SYM+I+G4 | 423 | *cytb*_codonA, *cox3*_codonA |
|  | 5 | GTR+F+I+G4 | 459 | *cytb*_codonB, *cox1*_codonB, *cox3*_codonB |
|  | 6 | GTR+F+I+G4 | 837 | *rrnS* |
|  | 7 | GTR+F+I+G4 | 140 | *trnV*, *trnD* |
|  | 8 | GTR+F+I+G4 | 1358 | *rrnL* |
|  | 9 | GTR+F+I+G4 | 515 | *nad1*_codonA, *atp6*_codonA, *nad3*_codonA |
|  | 10 | GTR+F+I+G4 | 413 | *nad1*_codonB, *atp6*_codonB, *nad3*_codonB |
|  | 11 | GTR+F+G4 | 165 | *trnI*, *nad4L*_codonA |
|  | 12 | GTR+F+I+G4 | 218 | *trnM*, *cox2*_codonA |
|  | 13 | GTR+F+I+G4 | 310 | *nad2*_codonA |
|  | 14 | GTR+F+I+G4 | 269 | *nad2*_codonB |
|  | 15 | SYM+I+G4 | 262 | *cox1*_codonA |
|  | 16 | GTR+F+G4 | 68 | *trnS2* |
|  | 17 | GTR+F+I+G4 | 124 | *cox2*_codonB |
|  | 18 | GTR+F+G4 | 62 | *trnR* |
|  | 19 | GTR+F+I+G4 | 419 | *nad4L*_codonB, *nad4*_codonB |
|  | 20 | GTR+F+I+G4 | 402 | *nad4*_codonA |
|  | 21 | SYM+G4 | 74 | *trnS1* |
| 11NT-ML | 1 | GTR+F+I+I+R7 | 174 | *nad6*_codon1 |
|  | 2 | GTR+F+I+I+R5 | 174 | *nad6*_codon2 |
|  | 3 | TIM2+F+I+I+R5 | 174 | *nad6*_codon3 |
|  | 4 | GTR+F+I+I+R6 | 384 | *cytb*_codon1 |
|  | 5 | GTR+F+I+I+R4 | 384 | *cytb*_codon2 |
|  | 6 | GTR+F+ASC+R9 | 384 | *cytb*_codon3 |
|  | 7 | GTR+F+I+I+R6 | 442 | *nad1*_codon1, *nad3*_codon1 |
|  | 8 | TVM+F+I+I+R5 | 328 | *nad1*_codon2 |
|  | 9 | GTR+F+ASC+R8 | 328 | *nad1*_codon3 |
|  | 10 | GTR+F+R7 | 348 | *nad2*_codon1 |
|  | 11 | GTR+F+R5 | 348 | *nad2*_codon2 |
|  | 12 | GTR+F+ASC+R7 | 348 | *nad2*_codon3 |
|  | 13 | SYM+I+I+R6 | 522 | *cox1*_codon1 |
|  | 14 | TVM+F+I+I+R4 | 522 | *cox1*_codon2 |
|  | 15 | TIM3+F+I+I+R7 | 522 | *cox1*_codon3 |
|  | 16 | GTR+F+I+I+R5 | 229 | *cox2*_codon1 |
|  | 17 | TIM3+F+R4 | 229 | *cox2*_codon2 |
|  | 18 | TIM3+F+ASC+R7 | 229 | *cox2*_codon3 |
|  | 19 | GTR+F+I+I+R5 | 231 | *atp6*_codon1 |
|  | 20 | TIM3+F+I+I+R5 | 231 | *atp6*_codon2 |
|  | 21 | TIM3+F+R8 | 231 | *atp6*_codon3 |
|  | 22 | SYM+I+I+R6 | 261 | *cox3*_codon1 |
|  | 23 | GTR+F+I+I+R5 | 261 | *cox3*_codon2 |
|  | 24 | TIM3+F+I+I+R7 | 261 | *cox3*_codon3 |
|  | 25 | TVM+F+R5 | 114 | *nad3*_codon2 |
|  | 26 | TN+F+ASC+R7 | 114 | *nad3*_codon3 |
|  | 27 | GTR+F+R5 | 100 | *nad4L*_codon1 |
|  | 28 | GTR+F+R4 | 100 | *nad4L*_codon2 |
|  | 29 | TIM3+F+ASC+R6 | 100 | *nad4L*_codon3 |
|  | 30 | GTR+F+R7 | 459 | *nad4*_codon1 |
|  | 31 | GTR+F+I+I+R6 | 459 | *nad4*_codon2 |
|  | 32 | GTR+F+I+I+R6 | 459 | *nad4*_codon3 |
| 11NT-BI | 1 | GTR+F+I+G4 | 160 | *nad6*_codon1 |
|  | 2 | GTR+F+I+G4 | 150 | *nad6*_codon2 |
|  | 3 | GTR+F+I+G4 | 174 | *nad6*_codon3 |
|  | 4 | SYM+I+G4 | 581 | *cytb*_codon1, *cox2*_codon1, *cox3*_codon1 |
|  | 5 | GTR+F+I+G4 | 376 | *cytb*_codon2, *nad1*_codon2 |
|  | 6 | GTR+F+G4 | 384 | *cytb*_codon3 |
|  | 7 | GTR+F+I+G4 | 515 | *nad1*_codon1, *atp6*_codon1, *nad3*_codon1 |
|  | 8 | GTR+F+G4 | 328 | *nad1*_codon3 |
|  | 9 | GTR+F+I+G4 | 708 | *nad2*_codon1, *nad4*_codon1 |
|  | 10 | GTR+F+I+G4 | 535 | *nad2*_codon2, *cox2*_codon2, *atp6*_codon2 |
|  | 11 | GTR+F+G4 | 348 | *nad2*_codon3 |
|  | 12 | SYM+I+G4 | 262 | *cox1*_codon1 |
|  | 13 | GTR+F+I+G4 | 270 | *cox1*_codon2, *cox3*_codon2 |
|  | 14 | GTR+F+I+G4 | 521 | *cox1*_codon3 |
|  | 15 | GTR+F+ASC+G4 | 229 | *cox2*_codon3 |
|  | 16 | GTR+F+I+G4 | 344 | *atp6*_codon3, *nad3*_codon3 |
|  | 17 | GTR+F+I+G4 | 261 | *cox3*_codon3 |
|  | 18 | GTR+F+G4 | 79 | *nad3*_codon2 |
|  | 19 | GTR+F+G4 | 96 | *nad4L*_codon1 |
|  | 20 | GTR+F+G4 | 84 | *nad4L*_codon2 |
|  | 21 | GTR+F+G4 | 100 | *nad4L*_codon3 |
|  | 22 | GTR+F+I+G4 | 342 | *nad4*_codon2 |
|  | 23 | GTR+F+I+G4 | 459 | *nad4*_codon3 |
| 11AA-ML | 1 | mtVer+F+R8 | 174 | *nad6* |
|  | 2 | mtVer+I+I+R10 | 2976 | *cytb*, *nad1*, *nad2*, *cox1*, *cox2*, *atp6*, *cox3*, *nad3*, *nad4L*, *nad4* |
| 11NT-BI | 1 | JTT+F+I+G4 | 173 | *nad6* |
|  | 2 | mtMAM+F+I+G4 | 2154 | *cytb*, *nad1*, *nad2*, *cox1*, *cox2*, *atp6*, *cox3*, *nad4L*, *nad4* |
|  | 3 | mtMAM+I+G4 | 104 | *nad3* |
