# Supplementary material for: Mitochondrial Phylogenomics and Genome Evolution in Anura: Insights From Structure and Gene Order Rearrangements
Source: Ecol Evol. 2026 Mar 30;16(4):e73370. doi: 10.1002/ece3.73370 (PMC13107284; doi:10.1002/ece3.73370)
Supplement: Supplementary file 24 — Table S3: Nucleotide composition analysis based on 277 anuran species. [file ECE3-16-e73370-s016.docx]

| **Species** | **Size(bp)** | A (%) | T (%) | C (%) | G (%) | AT% | GC% | AT skew | GC skew |
| --- | --- | --- | --- | --- | --- | --- | --- | --- | --- |
| *Alytes obstetricans* | 17490 | 29.5 | 26.8 | 28.8 | 14.9 | 56.3 | 43.7 | 0.05 | -0.32 |
| *Discoglossus galganoi* | 17014 | 28.6 | 27.7 | 27.1 | 16.6 | 56.3 | 43.7 | 0.015 | -0.24 |
| *Discoglossus pictus* | 20250 | 29.3 | 29.7 | 25.4 | 15.5 | 59 | 40.9 | -0.007 | -0.242 |
| *Astylosternus robustus* | 21418 | 29.6 | 27.9 | 29.3 | 13.2 | 57.5 | 42.5 | 0.029 | -0.379 |
| *Bombina bombina* | 17154 | 30 | 27.7 | 26.5 | 15.8 | 57.7 | 42.3 | 0.04 | -0.253 |
| *Bombina fortinuptialis* | 17575 | 30.5 | 28.7 | 25.4 | 15.3 | 59.2 | 40.7 | 0.029 | -0.248 |
| *Bombina lichuanensis* | 18554 | 31 | 29 | 25.1 | 14.9 | 60 | 40 | 0.032 | -0.255 |
| *Bombina maxima* | 18388 | 31.1 | 29.2 | 24.7 | 14.9 | 60.3 | 39.6 | 0.03 | -0.248 |
| *Bombina microdeladigitora* | 18256 | 30.6 | 29 | 25.3 | 15.1 | 59.6 | 40.4 | 0.027 | -0.252 |
| *Bombina orientalis* | 17847 | 30 | 27.8 | 26.7 | 15.5 | 57.8 | 42.2 | 0.037 | -0.264 |
| *Bombina variegata* | 18551 | 30 | 27.9 | 26.1 | 16 | 57.9 | 42.1 | 0.036 | -0.24 |
| *Ischnocnema guentheri* | 18383 | 29.5 | 30.6 | 26.1 | 13.7 | 60.2 | 39.8 | -0.018 | -0.311 |
| *Ischnocnema henselii* | 18536 | 30.3 | 30.3 | 26.4 | 13.0 | 60.6 | 39.4 | 0.001 | -0.341 |
| *Ischnocnema nasuta* | 19693 | 28.9 | 29.4 | 28.0 | 13.8 | 58.3 | 41.7 | -0.009 | -0.341 |
| *Eleutherodactylus coqui* | 18519 | 29.5 | 30.5 | 25.7 | 14.3 | 60.0 | 40.0 | -0.018 | -0.284 |
| *Breviceps adspersus* | 28757 | 31.7 | 28.7 | 27 | 12.7 | 60.4 | 39.7 | 0.05 | -0.36 |
| *Breviceps mossambicus* | 22543 | 32.5 | 28.1 | 27.0 | 12.4 | 60.6 | 39.4 | 0.072 | -0.372 |
| *Breviceps poweri* | 28059 | 27.8 | 29.8 | 30.3 | 12.1 | 57.6 | 42.4 | -0.035 | -0.430 |
| *Anaxyrus americanus* | 17328 | 30 | 32.4 | 23.5 | 14.1 | 62.4 | 37.6 | -0.038 | -0.248 |
| *Bufo bufo* | 17865 | 28.8 | 28.5 | 27.3 | 15.4 | 57.3 | 42.7 | 0.005 | -0.279 |
| *Bufo gargarizans* | 17277 | 28.9 | 28.1 | 27.7 | 15.3 | 57 | 43 | 0.014 | -0.288 |
| *Bufo minshanicus* | 17719 | 28.8 | 28.1 | 27.7 | 15.4 | 57.0 | 43.0 | 0.012 | -0.286 |
| *Bufo praetextatus* | 17757 | 28.9 | 28.1 | 27.6 | 15.3 | 57 | 42.9 | 0.014 | -0.286 |
| *Bufo stejnegeri* | 17939 | 28.3 | 28.6 | 27.4 | 15.7 | 56.9 | 43.1 | -0.006 | -0.271 |
| *Bufo tibetanus* | 17405 | 29 | 28.2 | 27.6 | 15.2 | 57.2 | 42.8 | 0.015 | -0.289 |
| *Bufotes pewzowi* | 17551 | 29.5 | 29.6 | 26 | 14.9 | 59.1 | 40.9 | -0.002 | -0.271 |
| *Bufotes pseudoraddei* | 17189 | 29.8 | 29.9 | 25.6 | 14.8 | 59.7 | 40.4 | -0.001 | -0.268 |
| *Bufotes viridis* | 17135 | 29.5 | 29.7 | 25.6 | 15.1 | 59.2 | 40.7 | -0.003 | -0.257 |
| *Bufotes turanensis* | 17225 | 30 | 30 | 25.4 | 14.7 | 60 | 40.1 | 0 | -0.267 |
| *Duttaphrynus melanostictus* | 17374 | 29.2 | 28.7 | 26.9 | 15.3 | 57.9 | 42.2 | 0.009 | -0.276 |
| *Melanophryniscus moreirae* | 18005 | 30.3 | 31.3 | 24.2 | 14.2 | 61.6 | 38.4 | -0.015 | -0.261 |
| *Rhinella marina* | 18152 | 29.9 | 31.4 | 24.5 | 14.2 | 61.3 | 38.7 | -0.024 | -0.265 |
| *Strauchbufo raddei* | 17602 | 29.2 | 29.6 | 26.1 | 15.2 | 58.8 | 41.3 | -0.008 | -0.265 |
| *Calyptocephalella gayi* | 17994 | 29.6 | 25.8 | 29.8 | 14.9 | 55.4 | 44.7 | 0.069 | -0.332 |
| *Telmatobufo australis* | 17989 | 28.9 | 26.6 | 29.2 | 15.2 | 55.5 | 44.4 | 0.041 | -0.315 |
| *Cornufer vitianus* | 17106 | 29.3 | 30.4 | 26.3 | 14 | 59.7 | 40.3 | -0.018 | -0.307 |
| *Anomaloglossus baeobatrachus* | 17572 | 28.5 | 30.3 | 27.4 | 13.9 | 58.8 | 41.3 | -0.031 | -0.326 |
| *Anomaloglossus blanci* | 16284 | 29 | 30.1 | 27.5 | 13.5 | 59.1 | 41 | -0.019 | -0.34 |
| *Anomaloglossus degranvillei* | 17090 | 29 | 30.3 | 27.1 | 13.5 | 59.3 | 40.6 | -0.021 | -0.336 |
| *Anomaloglossus dewynteri* | 17256 | 29 | 30.2 | 27.1 | 13.7 | 59.2 | 40.8 | -0.022 | -0.33 |
| *Anomaloglossus surinamensis* | 17148 | 28.8 | 30.6 | 26.7 | 13.9 | 59.4 | 40.6 | -0.031 | -0.316 |
| *Phyllobates terribilis* | 17702 | 28.3 | 31.1 | 26 | 14.6 | 59.4 | 40.6 | -0.046 | -0.279 |
| *Ranitomeya imitator* | 19445 | 28.5 | 32.1 | 26.4 | 13.0 | 60.7 | 39.3 | -0.060 | -0.341 |
| *Hyloxalus subpunctatus* | 16751 | 26.9 | 30.6 | 27.6 | 14.9 | 57.5 | 42.5 | -0.064 | -0.3 |
| *Euphlyctis hexadactyla* | 20280 | 27.1 | 29.9 | 28 | 15 | 57 | 43 | -0.05 | -0.304 |
| *Fejervarya cancrivora* | 17843 | 26.7 | 30 | 28.2 | 15 | 56.7 | 43.2 | -0.058 | -0.304 |
| *Fejervarya limnocharis* | 17717 | 28.1 | 29.9 | 26.8 | 15.2 | 58 | 42 | -0.032 | -0.277 |
| *Fejervarya multistriata* | 17750 | 28 | 29.9 | 26.9 | 15.2 | 57.9 | 42.1 | -0.033 | -0.278 |
| *Hoplobatrachus chinensis* | 20282 | 26.1 | 26.7 | 31.1 | 16.1 | 52.8 | 47.2 | -0.012 | -0.318 |
| *Hoplobatrachus tigerinus* | 20462 | 27 | 28.4 | 29.3 | 15.2 | 55.4 | 44.5 | -0.025 | -0.319 |
| *Limnonectes bannaensis* | 16867 | 28.9 | 29.7 | 27.2 | 14.2 | 58.6 | 41.4 | -0.014 | -0.314 |
| *Limnonectes blythii* | 16949 | 29.8 | 31.1 | 25.9 | 13.2 | 60.9 | 39.1 | -0.021 | -0.325 |
| *Limnonectes fragilis* | 16640 | 28.1 | 29.8 | 27.5 | 14.6 | 57.9 | 42.1 | -0.03 | -0.308 |
| *Limnonectes fujianensis* | 18293 | 28.1 | 29 | 28 | 14.8 | 57.1 | 42.8 | -0.017 | -0.308 |
|  |  |  |  |  |  |  |  |  |  |
| *Minervarya manoharan* | 17654 | 25.7 | 29.6 | 28.6 | 16.1 | 55.3 | 44.7 | -0.072 | -0.28 |
| *Nanorana kangxianensis* | 17150 | 28 | 28.7 | 28.2 | 15.1 | 56.7 | 43.3 | -0.012 | -0.303 |
| *Nanorana parkeri* | 17837 | 27.7 | 30.1 | 26.6 | 15.6 | 57.8 | 42.2 | -0.042 | -0.261 |
| *Nanorana pleskei* | 17660 | 27.5 | 30 | 26.2 | 16.3 | 57.5 | 42.5 | -0.042 | -0.234 |
| *Nanorana taihangnica* | 17412 | 28.6 | 28.6 | 28.2 | 14.6 | 57.2 | 42.8 | 0 | -0.316 |
| *Nanorana ventripunctata* | 18373 | 27.6 | 31.5 | 25.8 | 15.2 | 59.1 | 41 | -0.066 | -0.258 |
| *Quasipaa boulengeri* | 17741 | 28.9 | 30.6 | 26.1 | 14.4 | 59.5 | 40.5 | -0.03 | -0.288 |
| *Quasipaa exilispinosa* | 17046 | 29.3 | 29.4 | 27.2 | 14.2 | 58.7 | 41.4 | -0.002 | -0.314 |
| *Quasipaa robertingeri* | 18520 | 28.6 | 31.7 | 25.4 | 14.3 | 60.3 | 39.7 | -0.051 | -0.281 |
| *Quasipaa spinosa* | 18012 | 29.4 | 30.2 | 26.3 | 14.1 | 59.6 | 40.4 | -0.014 | -0.303 |
| *Quasipaa yei* | 17072 | 28.6 | 29.4 | 27.3 | 14.7 | 58 | 42 | -0.015 | -0.3 |
| *Occidozyga martensii* | 18321 | 29.5 | 32.8 | 23.5 | 14.2 | 62.3 | 37.7 | -0.053 | -0.246 |
| *Occidozyga myanhessei* | 18348 | 29.9 | 33.1 | 22.7 | 14.4 | 63 | 37.1 | -0.051 | -0.225 |
| *Heleophryne regis* | 17620 | 29 | 27.8 | 28.6 | 14.6 | 56.8 | 43.2 | 0.022 | -0.322 |
| *Hemisus marmoratus* | 20093 | 28.9 | 31.1 | 26.7 | 13.4 | 60 | 40.1 | -0.037 | -0.333 |
| *Bokermannohyla alvarengai* | 17325 | 28.8 | 30.1 | 26 | 15.1 | 58.9 | 41.1 | -0.021 | -0.265 |
| *Dendropsophus ebraccatus* | 19264 | 29 | 31.3 | 25.3 | 14.5 | 60.3 | 39.8 | -0.038 | -0.271 |
| *Dryophytes andersonii* | 15424 | 29 | 28.2 | 27.5 | 15.2 | 57.2 | 42.7 | 0.013 | -0.287 |
| *Dryophytes femoralis* | 15415 | 29.2 | 30.2 | 25.6 | 14.9 | 59.4 | 40.5 | -0.017 | -0.264 |
| *Dryophytes japonicus* | 19519 | 29.6 | 30.5 | 25.4 | 14.5 | 60.1 | 39.9 | -0.015 | -0.274 |
| *Dryophytes suweonensis* | 17448 | 29.3 | 28.6 | 27.2 | 15 | 57.9 | 42.2 | 0.013 | -0.291 |
| *Dryophytes versicolor* | 18800 | 30.5 | 31.1 | 24.6 | 13.8 | 61.6 | 38.4 | -0.009 | -0.282 |
| *Hyla annectans* | 17973 | 30.1 | 30.6 | 24.9 | 14.5 | 60.7 | 39.4 | -0.009 | -0.263 |
| *Hyla chinensis* | 18180 | 30 | 29.9 | 25.7 | 14.3 | 59.9 | 40 | 0.002 | -0.285 |
| *Hyla sanchiangensis* | 15694 | 29.9 | 29.8 | 25.7 | 14.6 | 59.7 | 40.3 | 0.001 | -0.275 |
| *Hyla sarda* | 18195 | 29.6 | 29.8 | 25.8 | 14.8 | 59.4 | 40.6 | -0.003 | -0.271 |
| *Hyla tsinlingensis* | 18295 | 29.3 | 28.6 | 27.1 | 15 | 57.9 | 42.1 | 0.012 | -0.288 |
| *Pseudis tocantins* | 15564 | 29.3 | 27.5 | 28.8 | 14.4 | 56.8 | 43.2 | 0.032 | -0.335 |
| *Phyllomedusa bahiana* | 16239 | 30.5 | 31.5 | 24.3 | 13.7 | 62 | 38 | -0.016 | -0.281 |
| *Pithecopus megacephalus* | 18050 | 30.1 | 30.5 | 25.6 | 13.8 | 60.6 | 39.4 | -0.007 | -0.299 |
| *Hyperolius marmoratus* | 22595 | 34.1 | 34.4 | 19.9 | 11.6 | 68.5 | 31.5 | -0.005 | -0.264 |
| *Leiopelma archeyi* | 16593 | 30.7 | 25.8 | 28.3 | 15.3 | 56.5 | 43.6 | 0.087 | -0.299 |
| *Leiopelma hochstetteri* | 17175 | 31.2 | 28.1 | 26.1 | 14.6 | 59.3 | 40.7 | 0.052 | -0.284 |
| *Leptodactylus fallax* | 18669 | 29.7 | 27.5 | 28 | 14.7 | 57.2 | 42.7 | 0.039 | -0.312 |
| *Leptodactylus fuscus* | 17473 | 29.2 | 29.5 | 26.3 | 15.0 | 58.7 | 41.3 | -0.006 | -0.273 |
| *Mantella baroni* | 20945 | 32.3 | 33.6 | 21.3 | 12.9 | 65.9 | 34.2 | -0.02 | -0.245 |
| *Mantella madagascariensis* | 22874 | 32.2 | 33.1 | 21.5 | 13.2 | 65.3 | 34.7 | -0.013 | -0.241 |
| *Leptobrachella alpina* | 17763 | 28.5 | 30.8 | 25.6 | 15.1 | 59.3 | 40.7 | -0.038 | -0.26 |
| *Leptobrachella oshanensis* | 17747 | 28.8 | 29.9 | 26.3 | 15.1 | 58.7 | 41.4 | -0.018 | -0.27 |
| *Leptobrachium ailaonicum* | 17318 | 27.9 | 31.8 | 25 | 15.3 | 59.7 | 40.3 | -0.066 | -0.241 |
| *Leptobrachium boringii* | 17085 | 27.7 | 31.5 | 25.5 | 15.4 | 59.2 | 40.9 | -0.065 | -0.247 |
| *Leptobrachium leishanense* | 17485 | 28.1 | 32.6 | 24.4 | 14.8 | 60.7 | 39.2 | -0.074 | -0.244 |
| *Leptobrachium liui* | 17190 | 28.1 | 32.6 | 24.4 | 14.9 | 60.7 | 39.3 | -0.074 | -0.243 |
| *Oreolalax jingdongensis* | 17864 | 29.1 | 32.7 | 23.9 | 14.3 | 61.8 | 38.2 | -0.059 | -0.253 |
| *Oreolalax lichuanensis* | 17702 | 28 | 32.2 | 24.9 | 15 | 60.2 | 39.9 | -0.069 | -0.248 |
| *Oreolalax major* | 17431 | 28.8 | 32.4 | 24.5 | 14.4 | 61.2 | 38.9 | -0.059 | -0.261 |
| *Oreolalax multipunctatus* | 17358 | 28.5 | 33 | 24.2 | 14.3 | 61.5 | 38.5 | -0.072 | -0.257 |
| *Oreolalax omeimontis* | 17675 | 28.5 | 32.6 | 25 | 14 | 61.1 | 39 | -0.068 | -0.282 |
| *Oreolalax rhodostigmatus* | 18676 | 28 | 32.4 | 24.9 | 14.7 | 60.4 | 39.6 | -0.072 | -0.259 |
| *Oreolalax schmidti* | 18481 | 28.3 | 32.8 | 24.5 | 14.4 | 61.1 | 38.9 | -0.073 | -0.259 |
| *Oreolalax xiangchengensis* | 17110 | 29.2 | 33 | 23.6 | 14.2 | 62.2 | 37.8 | -0.062 | -0.25 |
| *Scutiger ningshanensis* | 17265 | 29.1 | 32.7 | 24.2 | 14 | 61.8 | 38.2 | -0.058 | -0.269 |
| *Atympanophrys gigantica* | 18259 | 28.4 | 32.1 | 25.2 | 14.3 | 60.5 | 39.5 | -0.062 | -0.275 |
| *Atympanophrys shapingensis* | 17631 | 28.2 | 31.5 | 26 | 14.3 | 59.7 | 40.3 | -0.055 | -0.291 |
| *Boulenophrys baishanzuensis* | 17040 | 27.2 | 31.5 | 26.6 | 14.6 | 58.8 | 41.3 | -0.073 | -0.292 |
| *Boulenophrys boettgeri* | 16597 | 27.8 | 31.5 | 26.4 | 14.2 | 59.4 | 40.6 | -0.062 | -0.300 |
| *Boulenophrys kuatunensis* | 17921 | 28.1 | 32.1 | 25.9 | 14.0 | 60.2 | 39.8 | -0.066 | -0.298 |
| *Boulenophrys spinata* | 16024 | 27.2 | 30.6 | 27.1 | 15.1 | 57.8 | 42.2 | -0.06 | -0.284 |
| *Anilany helenae* | 17519 | 30.3 | 28.5 | 28.2 | 12.9 | 58.8 | 41.1 | 0.031 | -0.372 |
| *Glyphoglossus yunnanensis* | 16710 | 29.2 | 29.6 | 27 | 14.2 | 58.8 | 41.2 | -0.007 | -0.311 |
| *Kalophrynus palmatissimus* | 19702 | 30.2 | 31.6 | 25.3 | 12.9 | 61.8 | 38.2 | -0.023 | -0.323 |
| *Kaloula borealis* | 17174 | 29.7 | 29.5 | 26.6 | 14.2 | 59.2 | 40.8 | 0.003 | -0.305 |
| *Kaloula pulchra* | 16818 | 30.7 | 28.4 | 26.6 | 14.4 | 59.1 | 41 | 0.038 | -0.298 |
| *Kaloula rugifera* | 17074 | 29.7 | 30.3 | 25.8 | 14.2 | 60 | 40 | -0.01 | -0.289 |
| *Kaloula verrucosa* | 17061 | 29.6 | 30.8 | 25.4 | 14.2 | 60.4 | 39.6 | -0.019 | -0.284 |
| *Microhyla achatina* | 16725 | 28.9 | 29.3 | 27.2 | 14.6 | 58.2 | 41.8 | -0.008 | -0.302 |
| *Microhyla beilunensis* | 16721 | 29.1 | 31.9 | 24.5 | 14.5 | 61 | 39 | -0.046 | -0.256 |
| *Microhyla butleri* | 16722 | 28.8 | 29.6 | 26.9 | 14.7 | 58.4 | 41.6 | -0.014 | -0.294 |
| *Microhyla fissipes* | 16723 | 28.9 | 31 | 25.5 | 14.6 | 59.9 | 40.1 | -0.035 | -0.272 |
| *Microhyla heymonsi* | 16707 | 29 | 32.2 | 24.4 | 14.4 | 61.2 | 38.8 | -0.052 | -0.26 |
| *Microhyla mixtura* | 16729 | 29.5 | 31.4 | 24.9 | 14.2 | 60.9 | 39.1 | -0.031 | -0.272 |
| *Microhyla okinavensis* | 16717 | 29 | 31.8 | 24.6 | 14.5 | 60.8 | 39.1 | -0.047 | -0.257 |
| *Microhyla ornata* | 16730 | 29 | 31.1 | 25.3 | 14.6 | 60.1 | 39.9 | -0.035 | -0.27 |
| *Microhyla pulchra* | 16744 | 28.1 | 30.3 | 26.8 | 14.8 | 58.4 | 41.6 | -0.037 | -0.287 |
| *Microhyla taraiensis* | 16764 | 29.7 | 31.7 | 24.6 | 14 | 61.4 | 38.6 | -0.031 | -0.275 |
| *Platyplectrum melanopyga* | 19205 | 28.5 | 30 | 26.8 | 14.7 | 58.5 | 41.5 | -0.027 | -0.29 |
| *Mixophyes australis* | 18981 | 29.0 | 28.6 | 28.3 | 14.1 | 57.6 | 42.4 | 0.007 | -0.335 |
| *Taudactylus pleione* | 22974 | 29.9 | 28.2 | 14.0 | 27.9 | 58.1 | 41.9 | 0.029 | 0.331 |
| *Pelobates cultripes* | 17384 | 28.7 | 30.5 | 26.3 | 14.5 | 59.2 | 40.8 | -0.029 | -0.29 |
| *Pelobates fuscus* | 16828 | 28.9 | 30.4 | 26.4 | 14.3 | 59.3 | 40.7 | -0.026 | -0.299 |
| *Pelodytes ibericus* | 18155 | 32.0 | 31.3 | 23.2 | 13.5 | 63.3 | 36.7 | 0.012 | -0.263 |
| *Pelodytes punctatus* | 18905 | 32.4 | 32 | 22.5 | 13.1 | 64.4 | 35.6 | 0.007 | -0.263 |
| *Hymenochirus boettgeri* | 18007 | 33.7 | 25.9 | 26.7 | 13.7 | 59.6 | 40.4 | 0.131 | -0.323 |
| *Pseudhymenochirus merlini* | 18029 | 32.9 | 27.4 | 25 | 14.7 | 60.3 | 39.7 | 0.092 | -0.259 |
| *Xenopus allofraseri* | 17498 | 32.4 | 28.6 | 24.9 | 14.1 | 61 | 39 | 0.062 | -0.278 |
| *Xenopus amieti* | 16976 | 31.5 | 29.6 | 24.3 | 14.6 | 61.1 | 38.9 | 0.032 | -0.251 |
| *Xenopus andrei* | 17443 | 31.8 | 29.1 | 24.7 | 14.4 | 60.9 | 39.1 | 0.043 | -0.264 |
| *Xenopus borealis* | 17474 | 31.6 | 31.1 | 22.7 | 14.6 | 62.7 | 37.3 | 0.009 | -0.218 |
| *Xenopus boumbaensis* | 16807 | 31.3 | 29 | 25 | 14.8 | 60.3 | 39.8 | 0.039 | -0.256 |
| *Xenopus calcaratus* | 17523 | 30.3 | 26.5 | 28.4 | 14.9 | 56.8 | 43.3 | 0.067 | -0.312 |
| *Xenopus clivii* | 16794 | 31.8 | 29.8 | 24.1 | 14.3 | 61.6 | 38.4 | 0.033 | -0.253 |
| *Xenopus epitropicalis* | 17808 | 30.3 | 26.3 | 28.4 | 15 | 56.6 | 43.4 | 0.071 | -0.31 |
| *Xenopus eysoole* | 17397 | 31.6 | 29.6 | 24.2 | 14.6 | 61.2 | 38.8 | 0.033 | -0.247 |
| *Xenopus fischbergi* | 17189 | 31.7 | 30.9 | 22.9 | 14.5 | 62.6 | 37.4 | 0.012 | -0.224 |
| *Xenopus gilli* | 16923 | 32 | 29.5 | 24.4 | 14 | 61.5 | 38.4 | 0.042 | -0.27 |
| *Xenopus itombwensis* | 16853 | 31.5 | 28.9 | 24.9 | 14.8 | 60.4 | 39.7 | 0.042 | -0.256 |
| *Xenopus kobeli* | 17811 | 32.3 | 29.2 | 24.4 | 14 | 61.5 | 38.4 | 0.051 | -0.271 |
| *Xenopus laevis* | 17553 | 33.1 | 30 | 23.5 | 13.5 | 63.1 | 37 | 0.049 | -0.27 |
| *Xenopus largeni* | 17186 | 31.7 | 29.9 | 24.2 | 14.2 | 61.6 | 38.4 | 0.031 | -0.26 |
| *Xenopus lenduensis* | 17152 | 31.8 | 29 | 24.8 | 14.4 | 60.8 | 39.2 | 0.045 | -0.265 |
| *Xenopus longipes* | 17196 | 31.4 | 29.6 | 24.2 | 14.7 | 61 | 38.9 | 0.029 | -0.243 |
| *Xenopus mellotropicalis* | 17700 | 30.7 | 26.5 | 28.2 | 14.6 | 57.2 | 42.8 | 0.073 | -0.317 |
| *Xenopus muelleri* | 17478 | 31.1 | 30.3 | 24 | 14.5 | 61.4 | 38.5 | 0.013 | -0.245 |
| *Xenopus parafraseri* | 16708 | 31.6 | 28.2 | 25.7 | 14.5 | 59.8 | 40.2 | 0.057 | -0.279 |
| *Xenopus petersii* | 17729 | 32.6 | 29.5 | 24.4 | 13.5 | 62.1 | 37.9 | 0.05 | -0.287 |
| *Xenopus poweri* | 17581 | 32.4 | 29.5 | 24.5 | 13.5 | 61.9 | 38 | 0.047 | -0.289 |
| *Xenopus pygmaeus* | 17189 | 32.2 | 28.4 | 25.1 | 14.2 | 60.6 | 39.3 | 0.062 | -0.278 |
| *Xenopus ruwenzoriensis* | 16645 | 31.3 | 29.2 | 24.8 | 14.7 | 60.5 | 39.5 | 0.036 | -0.256 |
| *Xenopus tropicalis* | 17610 | 31 | 26.5 | 28.1 | 14.4 | 57.5 | 42.5 | 0.079 | -0.32 |
| *Xenopus vestitus* | 16779 | 31.8 | 28.8 | 25 | 14.3 | 60.6 | 39.3 | 0.049 | -0.272 |
| *Xenopus victorianus* | 17716 | 32.6 | 29.5 | 24.5 | 13.4 | 62.1 | 37.9 | 0.05 | -0.291 |
| *Xenopus wittei* | 17715 | 32.2 | 28.8 | 24.7 | 14.4 | 61 | 39.1 | 0.056 | -0.265 |
| *Pipa carvalhoi* | 19534 | 28.2 | 25.8 | 30.2 | 15.8 | 54 | 46 | 0.043 | -0.311 |
| *Pipa myersi* | 17947 | 33.2 | 30 | 23.2 | 13.6 | 63.2 | 36.8 | 0.051 | -0.259 |
| *Pipa pipa* | 18208 | 31.3 | 24.7 | 28.1 | 15.9 | 56 | 44 | 0.119 | -0.276 |
| *Pipa snethlageae* | 18024 | 34.2 | 25.3 | 26.3 | 14.1 | 59.5 | 40.4 | 0.15 | -0.304 |
| *Pyxicephalus adspersus* | 24317 | 29.5 | 28.2 | 27.8 | 14.5 | 57.7 | 42.3 | 0.022 | -0.314 |
| *Ptychadena amharensis* | 16435 | 27.3 | 29.9 | 26.3 | 16.5 | 57.1 | 42.9 | -0.045 | -0.228 |
| *Ptychadena anchietae* | 15483 | 27.3 | 30.1 | 26.9 | 15.7 | 57.4 | 42.6 | -0.048 | -0.261 |
| *Ptychadena beka* | 15607 | 28.1 | 30.7 | 25.7 | 15.5 | 58.8 | 41.2 | -0.044 | -0.248 |
| *Ptychadena cooperi* | 15666 | 27.8 | 30.6 | 25.7 | 16.0 | 58.3 | 41.7 | -0.048 | -0.232 |
| *Ptychadena delphina* | 15496 | 27.5 | 31.1 | 25.7 | 15.7 | 58.6 | 41.4 | -0.060 | -0.241 |
| *Ptychadena doro* | 15481 | 27.5 | 31.0 | 25.7 | 15.9 | 58.4 | 41.6 | -0.060 | -0.235 |
| *Ptychadena erlangeri* | 15606 | 28.0 | 31.0 | 25.2 | 15.8 | 59.0 | 41.0 | -0.052 | -0.231 |
| *Ptychadena goweri* | 15598 | 27.8 | 30.7 | 25.9 | 15.6 | 58.5 | 41.5 | -0.050 | -0.248 |
| *Ptychadena harenna* | 15499 | 27.6 | 30.9 | 25.9 | 15.7 | 58.5 | 41.5 | -0.056 | -0.246 |
| *Ptychadena levenorum* | 15621 | 27.7 | 30.7 | 25.8 | 15.8 | 58.4 | 41.6 | -0.051 | -0.239 |
| *Ptychadena nana* | 15819 | 28.3 | 31.5 | 24.8 | 15.3 | 59.9 | 40.1 | -0.053 | -0.239 |
| *Ptychadena neumanni* | 15516 | 27.4 | 30.4 | 26.3 | 15.9 | 57.8 | 42.2 | -0.053 | -0.248 |
| *Ptychadena nuerensis* | 17118 | 27.6 | 28.4 | 27.9 | 16.1 | 55.9 | 44.1 | -0.015 | -0.268 |
| *Ptychadena robeensis* | 15661 | 28.0 | 30.1 | 26.4 | 15.6 | 58.1 | 41.9 | -0.037 | -0.258 |
| *Ptychadena wadei* | 15513 | 28.2 | 32.2 | 25.0 | 14.6 | 60.4 | 39.6 | -0.065 | -0.261 |
| *Amolops granulosus* | 17785 | 28.1 | 28.5 | 28.4 | 15 | 56.6 | 43.4 | -0.007 | -0.309 |
| *Amolops jinjiangensis* | 17780 | 28 | 28 | 28.9 | 15.1 | 56 | 44 | 0 | -0.315 |
| *Amolops loloensis* | 18926 | 28.5 | 28.7 | 28.3 | 14.6 | 57.2 | 42.9 | -0.003 | -0.319 |
| *Amolops mantzorum* | 17744 | 28.3 | 28 | 28.8 | 14.8 | 56.3 | 43.6 | 0.005 | -0.321 |
| *Amolops ricketti* | 17772 | 28.9 | 30.5 | 26 | 14.5 | 59.4 | 40.5 | -0.027 | -0.285 |
| *Amolops sinensis* | 18084 | 29.4 | 30.7 | 25.3 | 14.7 | 60.1 | 40 | -0.022 | -0.265 |
| *Amolops wuyiensis* | 17797 | 28.9 | 30.9 | 25.6 | 14.6 | 59.8 | 40.2 | -0.033 | -0.272 |
| *Amerana draytonii* | 17805 | 27.7 | 29.1 | 28.8 | 14.4 | 56.8 | 43.2 | -0.025 | -0.333 |
| *Aquarana catesbeiana*_1 | 18241 | 28.6 | 31.2 | 26 | 14.1 | 59.8 | 40.1 | -0.045 | -0.296 |
| *Aquarana catesbeiana*_2 | 17212 | 28.6 | 31.1 | 26 | 14.3 | 59.7 | 40.3 | -0.043 | -0.289 |
| *Aquarana clamitans* | 17656 | 28.9 | 31.4 | 25.5 | 14.2 | 60.3 | 39.7 | -0.041 | -0.284 |
| *Aquarana okaloosae* | 17504 | 28.9 | 31.2 | 25.7 | 14.2 | 60.1 | 39.9 | -0.039 | -0.288 |
| *Babina holsti* | 19113 | 29.1 | 30.1 | 25.9 | 14.8 | 59.2 | 40.7 | -0.017 | -0.272 |
| *Babina subaspera* | 18525 | 29.4 | 30.7 | 25.4 | 14.5 | 60.1 | 39.9 | -0.021 | -0.274 |
| *Boreorana sylvatica* | 17343 | 28.6 | 30.7 | 26.3 | 14.4 | 59.3 | 40.7 | -0.036 | -0.293 |
| *Glandirana emeljanovi* | 17733 | 28.1 | 30.3 | 26.8 | 14.8 | 58.4 | 41.6 | -0.038 | -0.29 |
| *Glandirana rugosa* | 17781 | 28.1 | 30.5 | 27 | 14.4 | 58.6 | 41.4 | -0.041 | -0.302 |
| *Glandirana tientaiensis* | 17681 | 28.2 | 28.9 | 28.9 | 14 | 57.1 | 42.9 | -0.013 | -0.347 |
| *Hylarana guentheri* | 19053 | 29.8 | 29.5 | 26.9 | 13.8 | 59.3 | 40.7 | 0.005 | -0.32 |
| *Hylarana labialis* | 16184 | 30 | 31.1 | 25.8 | 13.1 | 61.1 | 38.9 | -0.019 | -0.326 |
| *Hylarana latouchii* | 17007 | 29.2 | 29.3 | 27.3 | 14.1 | 58.5 | 41.4 | -0.001 | -0.318 |
| *Nidirana adenopleura* | 18982 | 29.1 | 29.8 | 26.6 | 14.4 | 58.9 | 41 | -0.011 | -0.297 |
| *Nidirana daunchina* | 23131 | 30.6 | 30.4 | 25.4 | 13.7 | 60.9 | 39.1 | 0.003 | -0.297 |
| *Nidirana okinavana* | 19959 | 29.3 | 31 | 25.2 | 14.6 | 60.3 | 39.8 | -0.028 | -0.267 |
| *Nidirana yeae* | 19003 | 29 | 29.9 | 26.5 | 14.5 | 58.9 | 41 | -0.016 | -0.293 |
| *Odorrana exiliversabilis* | 17122 | 28.5 | 28.3 | 28.3 | 14.9 | 56.8 | 43.2 | 0.005 | -0.308 |
| *Odorrana grahami* | 17864 | 28.3 | 27.6 | 15.5 | 28.6 | 55.9 | 44.1 | 0.014 | 0.299 |
| *Odorrana graminea* | 18106 | 29.4 | 27.9 | 28 | 14.7 | 57.3 | 42.7 | 0.027 | -0.309 |
| *Odorrana hainanensis* | 17986 | 28.7 | 28 | 28.3 | 15 | 56.7 | 43.3 | 0.011 | -0.306 |
| *Odorrana hejiangensis* | 24227 | 29 | 29.6 | 25.8 | 15.6 | 58.6 | 41.4 | -0.01 | -0.246 |
| *Odorrana hosii* | 16035 | 29.3 | 28.8 | 27.2 | 14.6 | 58.1 | 41.8 | 0.008 | -0.3 |
| *Odorrana ishikawae* | 21020 | 29.2 | 30.1 | 26.4 | 14.3 | 59.3 | 40.7 | -0.015 | -0.299 |
| *Odorrana jingdongensis* | 17937 | 27.5 | 28.5 | 28.5 | 15.5 | 56 | 44 | -0.017 | -0.295 |
| *Odorrana livida* | 16057 | 28.9 | 27.6 | 28.5 | 15 | 56.5 | 43.5 | 0.023 | -0.311 |
| *Odorrana macrotympana* | 17767 | 30.1 | 29.4 | 14.4 | 26.2 | 59.5 | 40.6 | 0.012 | 0.289 |
| *Odorrana margaretae* | 17903 | 27.5 | 28.5 | 28.5 | 15.5 | 56 | 44 | -0.018 | -0.295 |
| *Odorrana nasuta* | 16535 | 28.7 | 27.7 | 28.8 | 14.9 | 56.4 | 43.6 | 0.018 | -0.318 |
| *Odorrana schmackeri* | 18302 | 29.4 | 28.4 | 27.4 | 14.8 | 57.8 | 42.2 | 0.018 | -0.298 |
| *Odorrana tormota* | 17962 | 28.5 | 29.4 | 27.3 | 14.8 | 57.9 | 42.1 | -0.016 | -0.296 |
| *Odorrana wuchuanensis* | 18256 | 27.9 | 27.8 | 29 | 15.3 | 55.7 | 44.3 | 0.002 | -0.308 |
| *Pelophylax cerigensis* | 17922 | 27.7 | 30.8 | 26.2 | 15.2 | 58.5 | 41.4 | -0.053 | -0.267 |
| *Pelophylax cretensis* | 17829 | 28.3 | 30.3 | 26.1 | 15.3 | 58.6 | 41.4 | -0.033 | -0.259 |
| *Pelophylax cypriensis* | 18023 | 28.2 | 31 | 25.8 | 14.9 | 59.2 | 40.7 | -0.048 | -0.267 |
| *Pelophylax epeiroticus* | 18030 | 27.8 | 30 | 27 | 15.3 | 57.8 | 42.3 | -0.037 | -0.276 |
| *Pelophylax kurtmuelleri* | 18020 | 27.9 | 30.5 | 26.4 | 15.2 | 58.4 | 41.6 | -0.045 | -0.271 |
| *Pelophylax lessonae* | 17968 | 27.9 | 30.3 | 26.7 | 15 | 58.2 | 41.7 | -0.041 | -0.279 |
| *Pelophylax nigromaculatus* | 17804 | 27.3 | 29.6 | 28.1 | 15 | 56.9 | 43.1 | -0.04 | -0.303 |
| *Pelophylax plancyi* | 17822 | 27.1 | 29.5 | 28 | 15.3 | 56.6 | 43.3 | -0.042 | -0.294 |
| *Pelophylax ridibundus* | 17990 | 28.2 | 30.2 | 26.6 | 15 | 58.4 | 41.6 | -0.033 | -0.28 |
| *Pelophylax shqipericus* | 17366 | 27.8 | 30.2 | 26.9 | 15.1 | 58 | 42 | -0.042 | -0.28 |
| *Rana amurensis* | 18470 | 28.4 | 30.1 | 27.3 | 14.1 | 58.5 | 41.4 | -0.029 | -0.318 |
| *Rana chensinensis* | 18808 | 27.7 | 29.4 | 28.1 | 14.7 | 57.1 | 42.8 | -0.03 | -0.311 |
| *Rana chaochiaoensis* | 18591 | 27.8 | 28.8 | 28.4 | 14.9 | 56.6 | 43.3 | -0.019 | -0.31 |
| *Rana coreana* | 22262 | 29.7 | 29.5 | 26.8 | 14 | 59.2 | 40.8 | 0.003 | -0.315 |
| *Rana dabieshanensis* | 18291 | 27 | 28.6 | 29.3 | 15.2 | 55.6 | 44.5 | -0.029 | -0.317 |
| *Rana dybowskii* | 18864 | 27.7 | 29.2 | 27.9 | 15.2 | 56.9 | 43.1 | -0.027 | -0.293 |
| *Rana hanluica* | 19395 | 28.2 | 30.9 | 26.9 | 14 | 59.1 | 40.9 | -0.045 | -0.314 |
| *Rana huanrensis* | 19253 | 27.9 | 29.7 | 28 | 14.4 | 57.6 | 42.4 | -0.03 | -0.321 |
| *Rana johnsi* | 17873 | 28 | 28.3 | 29.4 | 14.2 | 56.3 | 43.6 | -0.006 | -0.349 |
| *Rana kukunoris* | 18863 | 27.9 | 29.5 | 28 | 14.6 | 57.4 | 42.6 | -0.028 | -0.316 |
| *Rana longicrus* | 17833 | 27.6 | 28 | 29.5 | 14.9 | 55.6 | 44.4 | -0.007 | -0.328 |
| *Rana omeimontis* | 19934 | 27.5 | 29 | 28.5 | 15.1 | 56.5 | 43.6 | -0.028 | -0.308 |
| *Rana pyrenaica* | 17211 | 27.8 | 27.8 | 29.4 | 14.9 | 55.6 | 44.3 | 0 | -0.327 |
| *Rana sangzhiensis* | 19207 | 27.3 | 29.3 | 28.5 | 14.9 | 56.6 | 43.4 | -0.035 | -0.315 |
| *Rana temporaria* | 16061 | 27.3 | 28.8 | 28.9 | 15.1 | 56.1 | 44 | -0.026 | -0.314 |
| *Rana uenoi* | 17370 | 27.8 | 29 | 28.3 | 15 | 56.8 | 43.3 | -0.021 | -0.309 |
| *Rana wuyiensis* | 17779 | 28 | 28.5 | 29.3 | 14.2 | 56.5 | 43.5 | -0.009 | -0.349 |
| *Rana zhenhaiensis* | 18006 | 27.4 | 27.6 | 29.9 | 15 | 55 | 44.9 | -0.003 | -0.331 |
| *Indirana semipalmdata* | 17052 | 28.5 | 27.2 | 14.4 | 29.9 | 55.7 | 44.3 | 0.025 | 0.349 |
| *Buergeria buergeri* | 19959 | 29.9 | 30.5 | 24.8 | 14.7 | 60.4 | 39.5 | -0.01 | -0.255 |
| *Buergeria japonica* | 22274 | 32.4 | 33.6 | 19.9 | 14.1 | 66 | 34 | -0.019 | -0.17 |
| *Gracixalus yunnanensis* | 17735 | 31.4 | 32.3 | 23 | 13.3 | 63.7 | 36.3 | -0.015 | -0.266 |
| *Polypedates braueri* | 19904 | 29.7 | 31.9 | 23.3 | 15.1 | 61.6 | 38.4 | -0.036 | -0.212 |
| *Polypedates impresus* | 19720 | 30.2 | 30.8 | 24.3 | 14.7 | 61 | 39 | -0.01 | -0.246 |
| *Polypedates leucomystax* | 19837 | 30.2 | 30.8 | 24.3 | 14.7 | 61 | 39 | -0.009 | -0.246 |
| *Polypedates megacephalus* | 19952 | 30.2 | 30.4 | 24.6 | 14.8 | 60.6 | 39.4 | -0.004 | -0.25 |
| *Polypedates mutus* | 20056 | 30.5 | 30.4 | 24.6 | 14.5 | 60.9 | 39.1 | 0.001 | -0.257 |
| *Rhacophorus rhodopus* | 15789 | 30.8 | 30.0 | 24.3 | 14.8 | 60.9 | 39.1 | 0.013 | -0.243 |
| *Zhangixalus arboreus* | 22236 | 32.9 | 31.6 | 22.2 | 13.3 | 64.5 | 35.5 | 0.02 | -0.25 |
| *Zhangixalus burmanus* | 19830 | 32.3 | 31.0 | 23.1 | 13.7 | 63.2 | 36.8 | 0.021 | -0.256 |
| *Zhangixalus chenfui* | 20520 | 33.5 | 30.8 | 23 | 12.7 | 64.3 | 35.7 | 0.041 | -0.29 |
| *Zhangixalus dennysi* | 18052 | 31.4 | 31 | 23.2 | 14.3 | 62.4 | 37.5 | 0.007 | -0.237 |
| *Zhangixalus dugritei* | 19412 | 32 | 30.2 | 23.9 | 13.9 | 62.2 | 37.8 | 0.028 | -0.265 |
| *Zhangixalus omeimontis* | 19604 | 32.5 | 30.5 | 23.3 | 13.7 | 63 | 37 | 0.03 | -0.26 |
| *Zhangixalus schlegelii* | 21359 | 31.8 | 30.3 | 23.3 | 14.7 | 62.1 | 38 | 0.025 | -0.226 |
| *Rhinophrynus dorsalis* | 17299 | 30.3 | 26 | 28.5 | 15.2 | 56.3 | 43.7 | 0.077 | -0.305 |
| *Scaphiopus holbrookii* | 16881 | 30.8 | 26.6 | 27.4 | 15.2 | 57.4 | 42.6 | 0.074 | -0.285 |
| *Spea hammondii* | 16682 | 29.8 | 27.1 | 27.9 | 15.2 | 56.9 | 43.1 | 0.047 | -0.295 |
| *Sooglossus thomasseti* | 16407 | 30.9 | 27.1 | 28.5 | 13.5 | 58 | 42 | 0.065 | -0.356 |
| *Telmatobius bolivianus* | 19295 | 31.2 | 31.4 | 23.7 | 13.7 | 62.6 | 37.4 | -0.004 | -0.269 |
| *Telmatobius chusmisensis* | 19312 | 30.4 | 30.7 | 24 | 13.4 | 61.1 | 37.4 | -0.005 | -0.282 |
