# Supplementary material for: Mitochondrial Phylogenomics and Genome Evolution in Anura: Insights From Structure and Gene Order Rearrangements
Source: Ecol Evol. 2026 Mar 30;16(4):e73370. doi: 10.1002/ece3.73370 (PMC13107284; doi:10.1002/ece3.73370)
Supplement: Supplementary file 25 — Table S4: Anura species with mitogenomes duplication or loss in this study. [file ECE3-16-e73370-s001.docx]

| **Type** | **Loss(-) or duplication(+)** | **Species** | **Taxon** | **GenBank No.** |
| --- | --- | --- | --- | --- |
| tRNA gene | M(+1) | *Cornufer vitianus* | Ceratobatrachidae | NC_027671.1 |
|  |  | *Euphlyctis hexadactyla* | Dicroglossidae | NC_014584.1 |
|  |  | *Fejervarya cancrivora* | Dicroglossidae | NC_012647.1 |
|  |  | *Fejervarya limnocharis* | Dicroglossidae | NC_005055.1 |
|  |  | *Fejervarya multistriata* | Dicroglossidae | NC_012647.1 |
|  |  | *Hoplobatrachus chinensis* | Dicroglossidae | NC_042258.1 |
|  |  | *Hoplobatrachus tigerinus* | Dicroglossidae | NC_014581.1 |
|  |  | *Minervarya manoharani* | Dicroglossidae | KY463521.1 |
|  |  | *Nanorana parkeri* | Dicroglossidae | NC_026789.1 |
|  |  | *Nanorana pleskei* | Dicroglossidae | NC_016119.1 |
|  |  | *Nanorana ventripunctata* | Dicroglossidae | NC_039094.1 |
|  |  | *Quasipaa boulengeri* | Dicroglossidae | NC_021937.1 |
|  |  | *Quasipaa exilispinosa* | Dicroglossidae | NC_056269.1 |
|  |  | *Quasipaa robertingeri* | Dicroglossidae | KY441640.1 |
|  |  | *Quasipaa spinosa* | Dicroglossidae | NC_013270.1 |
|  |  | *Quasipaa yei* | Dicroglossidae | NC_024843.1 |
|  |  | *Occidozyga martensii* | Dicroglossidae | NC_014685.1 |
|  |  | *Occidozyga myanhessei* | Dicroglossidae | NC_057992.1 |
|  |  | *Heleophryne regis* | Heleophrynidae | NC_019998.1 |
|  |  | *Hyperolius marmoratus* | Hyperoliidae | NC_023381.1 |
|  |  | *Mantella baroni* | Mantellidae | NC_039758.1 |
|  |  | *Mantella madagascariensis* | Mantellidae | NC_007888.1 |
|  |  | *Leptobrachium ailaonicum* | Megophryidae | MZ394043.1 |
|  |  | *Leptobrachium boringii* | Megophryidae | NC_024427.1 |
|  |  | *Leptobrachium leishanense* | Megophryidae | NC_031411.1 |
|  |  | *Leptobrachium liui* | Megophryidae | NC_057468.1 |
|  |  | *Oreolalax jingdongensis* | Megophryidae | MF953479.1 |
|  |  | *Oreolalax major* | Megophryidae | NC_030605.1 |
|  |  | *Oreolalax multipunctatus* | Megophryidae | NC_037382.1 |
|  |  | *Oreolalax omeimontis* | Megophryidae | NC_049862.1 |
|  |  | *Oreolalax rhodostigmatus* | Megophryidae | MF770485.1 |
|  |  | *Oreolalax schmidti* | Megophryidae | NC_056343.1 |
|  |  | *Oreolalax xiangchengensis* | Megophryidae | MH727696.1 |
|  | L1(+1)、F(+1)、H(+1)、S1(+1)、T(+1)、P(+1) | *Astylosternus robustus* | Arthroleptidae | NC_023382.1 |
|  | T(-1) | *Ischnocnema guentheri* | Brachycephalidae | MH492737.1 |
|  | T(+1)、F(+2)、V(+2)、P(+2)、N(+1)、W(+1)、S1(+1) | *Breviceps adspersus* | Brevicipitidae | NC_023379.1 |
|  | S1(+1) | *Breviceps mossambicus* | Brevicipitidae | LC498571.1 |
|  |  | *Breviceps poweri* | Brevicipitidae | LC498572.1 |
|  | M(+1)、T(-1) | *Nanorana taihangnica* | Dicroglossidae | NC_024272.1 |
|  | M(+1)、A(-1)、N(-1) | *Limnonectes bannaensis* | Dicroglossidae | AY899242.1 |
|  | M(+1)、C(-1) | *Limnonectes blythii* | Dicroglossidae | NC_068685.1 |
|  | M(+1)、A(-1)、N(-1)、Q(-1)、P(-)、C(-1)、Y(-1) | *Limnonectes fragilis* | Dicroglossidae | AY899241.1 |
|  | M(+1)、C(+3) | *Limnonectes fujianensis* | Dicroglossidae | NC_007440.2 |
|  | M(+1)、A(-1)、N(-1)、C(-1)、Q(-1)、T(-) | *Nanorana kangxianensis* | Dicroglossidae | MZ895123.1 |
|  | M(+2) | *Zhangixalus chenfui* | Rhacophoridae | NC_062878.1 |
|  | M(+1)、W(-1) | *Leptobrachella alpina* | Megophryidae | MW487804.1 |
|  |  | *Leptobrachella oshanensis* | Megophryidae | NC_020610.1 |
|  | L1(+1)、T(+1)、P(+1)、F(+1)、V(+1)、E(+1) | *Pyxicephalus adspersus* | Pyxicephalidae | NC_044480.1 |
|  | A(+1)、N(+1)、Y(+1) | *Odorrana exiliversabilis* | Ranidae | NC_053712.1 |
|  | E(+1)、H（+1） | *Babina holsti* | Ranidae | NC_022870.1 |
|  |  | *Babina subaspera* | Ranidae | NC_022871.1 |
|  | H(-1) | *Odorrana schmackeri* | Ranidae | NC_027827.1 |
|  | F(-1) | *Rana pyrenaica* | Ranidae | KU720300.1 |
|  | H(+1) | *Nidirana okinavana* | Ranidae | NC_022872.1 |
|  | N(-1) | *Indirana semipalmdata* | Ranixalidae | KX774468.1 |
| Protein-coding gene | *nad5*(-1) | *Rhacophorus rhodopus* | Rhacophoridae | OK181853.1 |
|  | *atp8*(-1) | *Ischnocnema henselii* | Brachycephalidae | MH492733.1 |
|  |  | *Polypedates braueri* | Rhacophoridae | NC_042797.1 |
|  |  | *Polypedates impresus* | Rhacophoridae | NC_062354.1 |
|  |  | *Polypedates leucomystax* | Rhacophoridae | NC_062356.1 |
|  |  | *Polypedates megacephalus* | Rhacophoridae | NC_043955.1 |
|  |  | *Polypedates mutus* | Rhacophoridae | NC_062355.1 |
|  | *nad5*(+1) | *Astylosternus robustus* | Arthroleptidae | NC_023382.1 |
|  |  | *Hoplobatrachus chinensis* | Dicroglossidae | NC_042258.1 |
|  |  | *Hoplobatrachus tigerinus* | Dicroglossidae | NC_014581.1 |
|  |  | *Odorrana hejiangensis* | Ranidae | MZ895124.1 |
|  | *nad2*(+1) | *Hyperolius marmoratus* | Hyperoliidae | NC_023381.1 |
|  | *cox3*(+1)、*cob*(+1)、*nad6*(+1) | *Pyxicephalus adspersus* | Pyxicephalidae | NC_044480.1 |
| rRNA gene | *rrnL*(+2)、*rrnS*(+2) | *Breviceps adspersus* | Brevicipitidae | NC_023379.1 |
|  | *rrnS*(+1)、*rrnL*(+1) | *Pyxicephalus adspersus* | Pyxicephalidae | NC_044480.1 |
| CR | CR(+1) | *Astylosternus robustus* | Arthroleptidae | NC_023382.1 |
|  |  | *Breviceps adspersus* | Brevicipitidae | NC_023379.1 |
|  |  | *Ranitomeya imitator* | Dendrobatidae | CM064416.1 |
|  |  | *Euphlyctis hexadactyla* | Dicroglossidae | NC_014584.1 |
|  |  | *Hoplobatrachus chinensis* | Dicroglossidae | NC_042258.1 |
|  |  | *Hoplobatrachus tigerinus* | Dicroglossidae | NC_014581.1 |
|  |  | *Limnonectes fujianensis* | Dicroglossidae | NC_007440.2 |
|  |  | *Hyperolius marmoratus* | Hyperoliidae | NC_023381.1 |
|  |  | *Leiopelma hochstetteri* | Leiopelmatidae | NC_027072.1 |
|  |  | *Mantella baroni* | Mantellidae | NC_039758.1 |
|  |  | *Mantella madagascariensis* | Mantellidae | NC_007888.1 |
|  |  | *Oreolalax schmidti* | Megophryidae | NC_056343.1 |
|  |  | *Kalophrynus palmatissimus* | Microhylidae | NC_068684.1 |
|  |  | *Taudactylus pleione* | Myobatrachidae | CM062684.1 |
|  |  | *Pyxicephalus adspersus* | Pyxicephalidae | NC_044480.1 |
|  |  | *Hylarana labialis* | Ranidae | NC_068686.1 |
|  |  | *Nidirana daunchina* | Ranidae | OR528757.1 |
|  |  | *Rana amurensis* | Ranidae | NC_030042.1 |
|  |  | *Rana coreana* | Ranidae | NC_068259.1 |
|  |  | *Polypedates braueri* | Rhacophoridae | NC_042797.1 |
|  |  | *Polypedates impresus* | Rhacophoridae | NC_062354.1 |
|  |  | *Polypedates leucomystax* | Rhacophoridae | NC_062356.1 |
|  |  | *Polypedates megacephalus* | Rhacophoridae | NC_043955.1 |
|  |  | *Polypedates mutus* | Rhacophoridae | NC_062355.1 |
|  |  | *Zhangixalus arboreus* | Rhacophoridae | LC565708.1 |
|  |  | *Zhangixalus burmanus* | Rhacophoridae | OR161035.1 |
|  |  | *Zhangixalus chenfui* | Rhacophoridae | NC_062878.1 |
|  |  | *Zhangixalus dugritei* | Rhacophoridae | MZ712011.1 |
|  |  | *Zhangixalus omeimontis* | Rhacophoridae | NC_046387.1 |
|  |  | *Zhangixalus schlegelii* | Rhacophoridae | NC_007178.1 |
|  | CR(-1) | *Dryophytes andersonii* | Hylidae | NC_063648.1 |
|  |  | *Dryophytes femoralis* | Hylidae | NC_063649.1 |
|  |  | *Pseudis tocantins* | Hylidae | NC_041426.1 |
|  |  | *Anilany helenae* | Microhylidae | MZ751042.1 |
|  |  | *Ptychadena amharensis* | Ptychadenidae | NC_082083.1 |
|  |  | *Ptychadena anchietae* | Ptychadenidae | NC_082083.1 |
|  |  | *Ptychadena beka* | Ptychadenidae | NC_082088.1 |
|  |  | *Ptychadena cooperi* | Ptychadenidae | NC_082084.1 |
|  |  | *Ptychadena delphina* | Ptychadenidae | NC_082091.1 |
|  |  | *Ptychadena doro* | Ptychadenidae | NC_082090.1 |
|  |  | *Ptychadena erlangeri* | Ptychadenidae | NC_082087.1 |
|  |  | *Ptychadena goweri* | Ptychadenidae | NC_082089.1 |
|  |  | *Ptychadena harenna* | Ptychadenidae | NC_082093.1 |
|  |  | *Ptychadena levenorum* | Ptychadenidae | NC_082085.1 |
|  |  | *Ptychadena nana* | Ptychadenidae | NC_082086.1 |
|  |  | *Ptychadena neumanni* | Ptychadenidae | NC_082092.1 |
|  |  | *Ptychadena nuerensis* | Ptychadenidae | NC_082081.1 |
|  |  | *Ptychadena robeensis* | Ptychadenidae | NC_082094.1 |
|  |  | *Ptychadena wadei* | Ptychadenidae | NC_082082.1 |
