# Supplementary material for: Mitochondrial Phylogenomics and Genome Evolution in Anura: Insights From Structure and Gene Order Rearrangements
Source: Ecol Evol. 2026 Mar 30;16(4):e73370. doi: 10.1002/ece3.73370 (PMC13107284; doi:10.1002/ece3.73370)
Supplement: Supplementary file 26 — Table S5: Start codons in 13 protein‐coding genes in mitochondrial genomes of 277 Anura species. [file ECE3-16-e73370-s021.docx]

| **PCGs** | **ATG** | | **ATA** | | **ATC** | | **ATT** | | **GTG** | | **TTG** | |
| --- | --- | --- | --- | --- | --- | --- | --- | --- | --- | --- | --- | --- |
| *nad1* | 166 | 59.93% | 22 | 7.94% | 15 | 5.42% | 19 | 6.86% | 31 | 11.19% | 24 | 8.66% |
| *nad2* | 110 | 39.71% | 21 | 7.58% | 9 | 3.25% | 134 | 48.38% | 3 | 1.08% | 0 | 0.00% |
| *nad3* | 220 | 79.42% | 10 | 3.61% | 2 | 0.72% | 11 | 3.97% | 34 | 12.28% | 0 | 0.00% |
| *nad4* | 272 | 98.19% | 0 | 0.00% | 0 | 0.00% | 0 | 0.00% | 5 | 1.81% | 0 | 0.00% |
| *nad4L* | 232 | 83.75% | 4 | 1.44% | 1 | 0.36% | 4 | 1.44% | 36 | 13.00% | 0 | 0.00% |
| *nad5* | 221 | 80.07% | 27 | 9.79% | 5 | 1.81% | 5 | 1.81% | 18 | 6.52% | 0 | 0.00% |
| *nad6* | 248 | 89.53% | 7 | 2.53% | 0 | 0.00% | 5 | 1.80% | 17 | 6.14% | 0 | 0.00% |
| *atp6* | 228 | 82.31% | 30 | 10.83% | 3 | 1.08% | 1 | 0.36% | 13 | 4.70% | 2 | 0.72% |
| *atp8* | 262 | 96.68% | 1 | 0.37% | 0 | 0.00% | 0 | 0.00% | 8 | 2.95% | 0 | 0.00% |
| *cox1* | 5 | 1.81% | 115 | 41.52% | 0 | 0.00% | 2 | 0.72% | 154 | 55.59% | 1 | 0.36% |
| *cox2* | 267 | 96.39% | 7 | 2.53% | 0 | 0.00% | 0 | 0.00% | 3 | 1.08% | 0 | 0.00% |
| *cox3* | 275 | 99.28% | 2 | 0.72% | 0 | 0.00% | 0 | 0.00% | 0 | 0.00% | 0 | 0.00% |
| *cytb* | 273 | 98.56% | 3 | 1.08% | 1 | 0.36% | 0 | 0.00% | 0 | 0.00% | 0 | 0.00% |
