# Supplementary material for: Mitochondrial Phylogenomics and Genome Evolution in Anura: Insights From Structure and Gene Order Rearrangements
Source: Ecol Evol. 2026 Mar 30;16(4):e73370. doi: 10.1002/ece3.73370 (PMC13107284; doi:10.1002/ece3.73370)
Supplement: Supplementary file 27 — Table S6: Stop codons in 13 protein‐coding genes in mitochondrial genomes of 277 Anura species. [file ECE3-16-e73370-s020.docx]

| **PCGs** | **T--** | | **TAA** | | **AGA** | | **AGG** | | **TAG** | | **TA-** | | **AG-** | |
| --- | --- | --- | --- | --- | --- | --- | --- | --- | --- | --- | --- | --- | --- | --- |
| *nad1* | 185 | 66.79% | 14 | 5.05% | 2 | 0.72% | 10 | 3.61% | 12 | 4.33% | 54 | 19.50% | 0 | 0.00% |
| *nad2* | 187 | 67.75% | 35 | 12.68% | 8 | 2.90% | 1 | 0.36% | 36 | 13.05% | 9 | 3.26% | 0 | 0.00% |
| *nad3* | 233 | 84.11% | 27 | 9.75% | 1 | 0.36% | 1 | 0.36% | 5 | 1.81% | 10 | 3.61% | 0 | 0.00% |
| *nad4* | 194 | 70.03% | 57 | 20.58% | 1 | 0.36% | 6 | 2.17% | 14 | 5.05% | 5 | 1.81% | 0 | 0.00% |
| *nad4L* | 1 | 0.36% | 247 | 89.17% | 0 | 0.00% | 0 | 0.00% | 24 | 8.66% | 5 | 1.81% | 0 | 0.00% |
| *nad5* | 50 | 18.12% | 98 | 35.51% | 57 | 20.65% | 52 | 18.84% | 18 | 6.52% | 1 | 0.36% | 0 | 0.00% |
| *nad6* | 0 | 0.00% | 16 | 5.80% | 146 | 52.90% | 98 | 35.50% | 16 | 5.80% | 0 | 0.00% | 0 | 0.00% |
| *atp6* | 133 | 48.02% | 102 | 36.82% | 2 | 0.72% | 3 | 1.08% | 6 | 2.17% | 31 | 11.19% | 0 | 0.00% |
| *atp8* | 0 | 0.00% | 238 | 87.82% | 1 | 0.37% | 0 | 0.00% | 32 | 11.81% | 0 | 0.00% | 0 | 0.00% |
| *cox1* | 4 | 1.45% | 78 | 28.26% | 23 | 8.33% | 159 | 57.61% | 11 | 3.99% | 0 | 0.00% | 1 | 0.36% |
| *cox2* | 238 | 85.92% | 25 | 9.03% | 11 | 3.97% | 0 | 0.00% | 3 | 1.08% | 0 | 0.00% | 0 | 0.00% |
| *cox3* | 233 | 84.12% | 2 | 0.72% | 0 | 0.00% | 0 | 0.00% | 0 | 0.00% | 42 | 15.16% | 0 | 0.00% |
| *cytb* | 42 | 15.22% | 136 | 49.28% | 21 | 7.61% | 1 | 0.36% | 37 | 13.40% | 39 | 14.13% | 0 | 0.00% |
