# Supplementary material for: Mitochondrial Phylogenomics and Genome Evolution in Anura: Insights From Structure and Gene Order Rearrangements
Source: Ecol Evol. 2026 Mar 30;16(4):e73370. doi: 10.1002/ece3.73370 (PMC13107284; doi:10.1002/ece3.73370)
Supplement: Supplementary file 28 — Table S7: The results of relative synonymous codon usage analysis for 13 PCGs of anuran species. All frequencies were averaged over all taxa. Relative synonymous codon usage was given in parentheses following the codon frequency. * represent stop codon. [file ECE3-16-e73370-s005.docx]

| Codon | Count | RSCU | Codon | Count | RSCU | Codon | Count | RSCU | Codon | Count | RSCU |
| --- | --- | --- | --- | --- | --- | --- | --- | --- | --- | --- | --- |
| UUU(F) | 153 | 1.19 | UCU(S) | 69.3 | 1.46 | UAU(Y) | 56.9 | 1.03 | UGU(C) | 12.4 | 0.84 |
| UUC(F) | 104 | 0.81 | UCC(S) | 66.2 | 1.4 | UAC(Y) | 54.2 | 0.97 | UGC(C) | 17.3 | 1.16 |
| UUA(L) | 142 | 1.39 | UCA(S) | 89.9 | 1.9 | UAA(*) | 0 | 0 | UGA(W) | 92.8 | 1.69 |
| UUG(L) | 28.7 | 0.28 | UCG(S) | 9 | 0.19 | UAG(*) | 0 | 0 | UGG(W) | 17 | 0.31 |
| CUU(L) | 142 | 1.39 | CCU(P) | 46.3 | 0.91 | CAU(H) | 34.2 | 0.7 | CGU(R) | 11.6 | 0.65 |
| CUC(L) | 111 | 1.08 | CCC(P) | 63.5 | 1.25 | CAC(H) | 63.8 | 1.3 | CGC(R) | 17.1 | 0.96 |
| CUA(L) | 151 | 1.48 | CCA(P) | 82.3 | 1.62 | CAA(Q) | 78.9 | 1.75 | CGA(R) | 37.9 | 2.12 |
| CUG(L) | 38.9 | 0.38 | CCG(P) | 11.8 | 0.23 | CAG(Q) | 11.2 | 0.25 | CGG(R) | 5.1 | 0.28 |
| AUU(I) | 204 | 1.27 | ACU(T) | 74.3 | 1.01 | AAU(N) | 57.9 | 0.88 | AGU(S) | 17.8 | 0.37 |
| AUC(I) | 118 | 0.73 | ACC(T) | 100 | 1.36 | AAC(N) | 73.2 | 1.12 | AGC(S) | 32 | 0.68 |
| AUA(M) | 127 | 1.47 | ACA(T) | 110 | 1.49 | AAA(K) | 73.8 | 1.74 | AGA(*) | 0 | 0 |
| AUG(M) | 46 | 0.53 | ACG(T) | 10.8 | 0.15 | AAG(K) | 11.2 | 0.26 | AGG(*) | 0 | 0 |
| GUU(V) | 63.8 | 1.32 | GCU(A) | 79.9 | 1.04 | GAU(D) | 28.8 | 0.81 | GGU(G) | 36.6 | 0.66 |
| GUC(V) | 43 | 0.89 | GCC(A) | 131 | 1.7 | GAC(D) | 42.4 | 1.19 | GGC(G) | 68.4 | 1.23 |
| GUA(V) | 64.6 | 1.34 | GCA(A) | 85.1 | 1.11 | GAA(E) | 69.7 | 1.54 | GGA(G) | 73.1 | 1.31 |
| GUG(V) | 21.8 | 0.45 | GCG(A) | 11.2 | 0.15 | GAG(E) | 20.8 | 0.46 | GGG(G) | 44.7 | 0.8 |
| Average codons=3759 | | | | | | | | | | | |
