# Supplementary material for: Mitochondrial Phylogenomics and Genome Evolution in Anura: Insights From Structure and Gene Order Rearrangements
Source: Ecol Evol. 2026 Mar 30;16(4):e73370. doi: 10.1002/ece3.73370 (PMC13107284; doi:10.1002/ece3.73370)
Supplement: Supplementary file 29 — Table S8: Divergence times of the major lineages analyzed in this study, estimated from Maximum likelihood phylogenetic analyses of the concatenated 24NT dataset. [file ECE3-16-e73370-s010.docx]

| Clade | Age | Lower 95% HPD | Upper 95% HPD |
| --- | --- | --- | --- |
| 1 | 315.68 | 307.78 | 325.34 |
| 2 | 262.64 | 252.16 | 272.78 |
| 3 | 221.09 | 202 | 239.28 |
| 4 | 55.3 | 30.04 | 90.22 |
| 5 | 204.66 | 187.38 | 221.98 |
| 6 | 182.56 | 153.28 | 209.36 |
| 7 | 40.13 | 29.78 | 51.7 |
| 8 | 12.24 | 7.67 | 17.76 |
| 9 | 3.71 | 2.39 | 5.18 |
| 10 | 2.57 | 1.47 | 3.91 |
| 11 | 29.56 | 20.72 | 39.7 |
| 12 | 16.37 | 9.36 | 23.77 |
| 13 | 159.54 | 121.14 | 190.98 |
| 14 | 32.07 | 17.02 | 47.84 |
| 15 | 197.14 | 180.29 | 213.55 |
| 16 | 173.54 | 147.44 | 199.32 |
| 17 | 132.31 | 114.4 | 149.83 |
| 18 | 78.84 | 58.07 | 101.07 |
| 19 | 55.46 | 37.81 | 73.96 |
| 20 | 36.28 | 22.11 | 51.39 |
| 21 | 124.57 | 105.92 | 142.19 |
| 22 | 89.64 | 71.96 | 110.21 |
| 23 | 37.31 | 25.58 | 49.08 |
| 24 | 17.47 | 9.58 | 25.55 |
| 25 | 16.88 | 9.54 | 25.48 |
| 26 | 60.91 | 49.27 | 72.45 |
| 27 | 33.48 | 23.65 | 44.29 |
| 28 | 23.74 | 14.95 | 33.25 |
| 29 | 52.47 | 43 | 62.76 |
| 30 | 42.81 | 35.8 | 50.82 |
| 31 | 37.39 | 31.05 | 44.02 |
| 32 | 33.75 | 27.24 | 40.08 |
| 33 | 13.67 | 7.98 | 19.89 |
| 34 | 27.2 | 21.05 | 34.07 |
| 35 | 16.44 | 11.23 | 21.99 |
| 36 | 6.97 | 4.42 | 9.69 |
| 37 | 5.87 | 3.41 | 8.51 |
| 38 | 25.01 | 20.4 | 30.32 |
| 39 | 20.63 | 16.5 | 24.72 |
| 40 | 17.47 | 13.66 | 21.37 |
| 41 | 10.34 | 7.8 | 13.03 |
| 42 | 8.19 | 6.03 | 10.53 |
| 43 | 3.48 | 1.93 | 5.18 |
| 44 | 5.76 | 3.73 | 7.84 |
| 45 | 0.98 | 0.5 | 1.56 |
| 46 | 19.71 | 15.73 | 23.89 |
| 47 | 15.36 | 10.9 | 19.99 |
| 48 | 13.83 | 9.16 | 18.5 |
| 49 | 89.78 | 63.5 | 116.22 |
| 50 | 187.24 | 171.92 | 203.49 |
| 51 | 145.98 | 136.25 | 154.21 |
| 52 | 125.67 | 105.81 | 141.74 |
| 53 | 51.55 | 38.82 | 74.48 |
| 54 | 7.18 | 3.73 | 10.97 |
| 55 | 131.06 | 119.57 | 141.34 |
| 56 | 26.66 | 14.4 | 40.99 |
| 57 | 118.54 | 106.85 | 130.27 |
| 58 | 97.89 | 85.06 | 110.97 |
| 59 | 75.03 | 62.66 | 87.77 |
| 60 | 67.82 | 56.31 | 80.61 |
| 61 | 44.23 | 36.26 | 53.24 |
| 62 | 37.36 | 30.01 | 45 |
| 63 | 18.14 | 11.08 | 25.78 |
| 64 | 33.39 | 26.33 | 41 |
| 65 | 13.47 | 7.96 | 19.34 |
| 66 | 17.91 | 11.87 | 23.89 |
| 67 | 9.82 | 5.47 | 14.38 |
| 68 | 21.15 | 14.99 | 27.49 |
| 69 | 17.52 | 12.11 | 23.45 |
| 70 | 10.54 | 6.17 | 15.29 |
| 71 | 27.57 | 16.01 | 40.61 |
| 72 | 48.71 | 36.98 | 61.95 |
| 73 | 32.56 | 23.66 | 41.76 |
| 74 | 21.54 | 15 | 29.06 |
| 75 | 12.38 | 7.36 | 17.98 |
| 76 | 10.43 | 5.38 | 15.92 |
| 77 | 169.77 | 155.34 | 183.53 |
| 78 | 163.52 | 150.42 | 177.32 |
| 79 | 158.74 | 145.64 | 171.31 |
| 80 | 142.45 | 130.27 | 152.73 |
| 81 | 112.07 | 93.74 | 130.6 |
| 82 | 85.94 | 66.37 | 107.88 |
| 83 | 70.16 | 47.08 | 91.67 |
| 84 | 66.39 | 52.78 | 84.05 |
| 85 | 126.31 | 113.64 | 137.99 |
| 86 | 102.85 | 86.66 | 120.04 |
| 87 | 50.97 | 36.77 | 66.13 |
| 88 | 32.7 | 20.89 | 45.24 |
| 89 | 119.35 | 106.74 | 131.33 |
| 90 | 51.11 | 30.53 | 74.41 |
| 91 | 113.12 | 100.85 | 125.9 |
| 92 | 78.91 | 64.53 | 94.73 |
| 93 | 67.18 | 51.66 | 83.12 |
| 94 | 51.21 | 36.12 | 67.79 |
| 95 | 47.38 | 34.94 | 61.03 |
| 96 | 26.12 | 17.5 | 35.6 |
| 97 | 8.25 | 5.44 | 11.41 |
| 98 | 5.95 | 3.56 | 8.75 |
| 99 | 105.78 | 92.56 | 118.51 |
| 100 | 97.91 | 84.45 | 112.04 |
| 101 | 93.66 | 79.4 | 107.01 |
| 102 | 85.09 | 70.23 | 98.72 |
| 103 | 52.19 | 41.86 | 63.21 |
| 104 | 38.87 | 29.54 | 48.38 |
| 105 | 25.73 | 17.79 | 33.7 |
| 106 | 19.64 | 12.39 | 27.39 |
| 107 | 34.24 | 25.98 | 42.56 |
| 108 | 28.71 | 21.34 | 36.84 |
| 109 | 22.41 | 14.73 | 29.8 |
| 110 | 1.4 | 0.7 | 2.17 |
| 111 | 23.94 | 16.23 | 31.95 |
| 112 | 99.47 | 86.11 | 112.28 |
| 113 | 7.81 | 4.21 | 12.16 |
| 114 | 93.92 | 81.35 | 107.21 |
| 115 | 56.42 | 37.62 | 76.88 |
| 116 | 83.56 | 70.31 | 96.71 |
| 117 | 55.72 | 46.04 | 65.93 |
| 118 | 49.79 | 40.92 | 58.96 |
| 119 | 42.98 | 35.47 | 51.33 |
| 120 | 37.38 | 29.15 | 45.84 |
| 121 | 10.09 | 6.8 | 13.75 |
| 122 | 2.05 | 1.04 | 3.16 |
| 123 | 6.23 | 3.62 | 9.21 |
| 124 | 40.1 | 32.3 | 47.83 |
| 125 | 27.44 | 21.01 | 34.29 |
| 126 | 16.61 | 11.85 | 21.53 |
| 127 | 13.28 | 9.04 | 17.98 |
| 128 | 3.15 | 2.04 | 4.42 |
| 129 | 1.86 | 1.05 | 2.81 |
| 130 | 145.51 | 133.14 | 157.32 |
| 131 | 137.13 | 123.98 | 148.85 |
| 132 | 129.88 | 116.71 | 143.12 |
| 133 | 108.35 | 90.05 | 125.01 |
| 134 | 48.54 | 34.39 | 63.84 |
| 135 | 35.75 | 22.54 | 49.16 |
| 136 | 108.61 | 88.65 | 126.85 |
| 137 | 118.94 | 103.8 | 134.41 |
| 138 | 104.5 | 89.41 | 120.68 |
| 139 | 90.21 | 75.43 | 104.73 |
| 140 | 43.61 | 30.02 | 58.68 |
| 141 | 20.79 | 13.66 | 28.57 |
| 142 | 15.14 | 8.58 | 21.84 |
| 143 | 77.4 | 63.59 | 90.31 |
| 144 | 61.18 | 50.11 | 72.52 |
| 145 | 52.91 | 43.17 | 62.68 |
| 146 | 44.35 | 34.05 | 55.29 |
| 147 | 44.24 | 36.11 | 53.18 |
| 148 | 33.63 | 26 | 41.38 |
| 149 | 6.23 | 3.33 | 9.51 |
| 150 | 24.57 | 18 | 31.79 |
| 151 | 16.36 | 10.63 | 22.85 |
| 152 | 36.2 | 27.47 | 46.08 |
| 153 | 137.24 | 125.35 | 148.53 |
| 154 | 132.86 | 121.3 | 144.05 |
| 155 | 121.82 | 109.72 | 134.52 |
| 156 | 86.87 | 72.35 | 102.36 |
| 157 | 70.47 | 56.7 | 84.8 |
| 158 | 54.57 | 39.87 | 69.69 |
| 159 | 39.4 | 31.92 | 46.84 |
| 160 | 31.87 | 25.67 | 38.17 |
| 161 | 26.75 | 20.65 | 32.75 |
| 162 | 19.37 | 13.9 | 25.02 |
| 163 | 15.3 | 10 | 20.73 |
| 164 | 21.95 | 16.9 | 26.9 |
| 165 | 16.37 | 11.98 | 20.81 |
| 166 | 14.64 | 10.38 | 18.89 |
| 167 | 7.91 | 4.68 | 11.41 |
| 168 | 15.98 | 10.68 | 21.62 |
| 169 | 26.28 | 17.61 | 35.04 |
| 170 | 128.12 | 117.04 | 139.26 |
| 171 | 123.97 | 112.87 | 135.02 |
| 172 | 114.61 | 103.14 | 126.12 |
| 173 | 34.48 | 20.65 | 48.33 |
| 174 | 103.45 | 91.1 | 114.73 |
| 175 | 88.42 | 75.9 | 101.68 |
| 176 | 59.85 | 47.41 | 72.61 |
| 177 | 41.95 | 31.43 | 52.81 |
| 178 | 34.06 | 24.07 | 44.47 |
| 179 | 10 | 5.52 | 15.28 |
| 180 | 6.92 | 3.83 | 10.6 |
| 181 | 38.01 | 28.51 | 47.54 |
| 182 | 24.29 | 17.31 | 32.34 |
| 183 | 15.12 | 9.35 | 21.74 |
| 184 | 14.87 | 7.93 | 22.19 |
| 185 | 61.75 | 47.62 | 76.12 |
| 186 | 52.21 | 38.53 | 65.9 |
| 187 | 33.38 | 21.71 | 45.7 |
| 188 | 83.83 | 70.55 | 96.16 |
| 189 | 67.39 | 53.66 | 80.58 |
| 190 | 44.55 | 31.93 | 58.16 |
| 191 | 0.55 | 0.26 | 0.9 |
| 192 | 61.77 | 45.72 | 76.19 |
| 193 | 32.03 | 20.17 | 45.7 |
| 194 | 123.33 | 111.86 | 134.09 |
| 195 | 113.23 | 101.47 | 124.79 |
| 196 | 32.48 | 19.37 | 48.02 |
| 197 | 101.06 | 89.2 | 113.48 |
| 198 | 83.14 | 71.35 | 95.12 |
| 199 | 74.8 | 63.57 | 86.66 |
| 200 | 67.05 | 55.88 | 78.97 |
| 201 | 44.56 | 35.7 | 53.12 |
| 202 | 37.9 | 30.04 | 46.17 |
| 203 | 27.03 | 20.77 | 33.75 |
| 204 | 22.41 | 15.87 | 28.59 |
| 205 | 12.8 | 7.45 | 18.38 |
| 206 | 22.94 | 16.51 | 29.99 |
| 207 | 35.14 | 26.31 | 44.35 |
| 208 | 16.51 | 11.32 | 22.42 |
| 209 | 11.51 | 6.83 | 16.71 |
| 210 | 26 | 17.11 | 35.45 |
| 211 | 63.12 | 41.45 | 84.64 |
| 212 | 87.39 | 76.29 | 98.57 |
| 213 | 80.87 | 68.99 | 92.33 |
| 214 | 57.71 | 43.12 | 73.23 |
| 215 | 4.31 | 2.33 | 6.74 |
| 216 | 33.71 | 25.01 | 43.12 |
| 217 | 23.15 | 17.42 | 29.61 |
| 218 | 17.65 | 13.03 | 22.74 |
| 219 | 11.32 | 8.32 | 14.43 |
| 220 | 9.52 | 6.83 | 12.41 |
| 221 | 7.65 | 5.07 | 10.19 |
| 222 | 3.64 | 2.06 | 5.41 |
| 223 | 64.54 | 49.05 | 78.91 |
| 224 | 21.99 | 14.43 | 30.28 |
| 225 | 14.92 | 8.5 | 21.76 |
| 226 | 11.9 | 8.31 | 15.73 |
| 227 | 9.37 | 6.28 | 12.7 |
| 228 | 5.77 | 3.37 | 8.57 |
| 229 | 82.26 | 71.61 | 92.67 |
| 230 | 76.3 | 65.56 | 88.02 |
| 231 | 56.92 | 42.23 | 71.94 |
| 232 | 42.07 | 29.6 | 54.03 |
| 233 | 35.75 | 23.74 | 47.88 |
| 234 | 77.8 | 67.92 | 88.2 |
| 235 | 55.9 | 40.32 | 70.54 |
| 236 | 22.17 | 15.14 | 29.64 |
| 237 | 6.86 | 3.71 | 10.29 |
| 238 | 10.02 | 6.55 | 13.76 |
| 239 | 7.38 | 4.28 | 10.7 |
| 240 | 7.98 | 4.15 | 12.16 |
| 241 | 74.73 | 64.92 | 84.7 |
| 242 | 56.53 | 48.23 | 65.52 |
| 243 | 49.64 | 42.11 | 57.58 |
| 244 | 27.03 | 18.84 | 36.92 |
| 245 | 13.26 | 7.55 | 19.59 |
| 246 | 0.44 | 0.19 | 0.72 |
| 247 | 44.65 | 37.52 | 51.82 |
| 248 | 33.06 | 25.47 | 41.48 |
| 249 | 28.03 | 20.15 | 36.11 |
| 250 | 38.98 | 32.3 | 45.83 |
| 251 | 34.27 | 27.1 | 40.78 |
| 252 | 22.54 | 15.45 | 29.65 |
| 253 | 0.66 | 0.3 | 1.07 |
| 254 | 27.42 | 20.12 | 34.71 |
| 255 | 12 | 6.83 | 17.54 |
| 256 | 62.15 | 52.92 | 71.63 |
| 257 | 46.71 | 34.24 | 59.37 |
| 258 | 11.35 | 6.35 | 16.75 |
| 259 | 1.81 | 0.91 | 2.82 |
| 260 | 0.15 | 0.03 | 0.3 |
| 261 | 52.7 | 44.39 | 60.8 |
| 262 | 44.64 | 37.91 | 51.76 |
| 263 | 4.26 | 2.21 | 6.59 |
| 264 | 39.59 | 33.36 | 45.84 |
| 265 | 17.93 | 9.63 | 26.75 |
| 266 | 35.66 | 29.91 | 41.57 |
| 267 | 23.05 | 17.54 | 28.45 |
| 268 | 13.61 | 10.16 | 17.25 |
| 269 | 0.92 | 0.43 | 1.45 |
| 270 | 12.23 | 8.91 | 15.75 |
| 271 | 4.46 | 2.48 | 6.6 |
| 272 | 5.29 | 3.04 | 7.84 |
| 273 | 33.34 | 27.74 | 39.33 |
| 274 | 23.57 | 17.85 | 29.48 |
| 275 | 17.79 | 11.99 | 23.78 |
| 276 | 8.33 | 5.52 | 11.65 |
| 277 | 4.89 | 2.68 | 7.18 |
| 278 | 18.23 | 12.08 | 24.66 |
