# Supplementary material for: Mitochondrial Phylogenomics and Genome Evolution in Anura: Insights From Structure and Gene Order Rearrangements
Source: Ecol Evol. 2026 Mar 30;16(4):e73370. doi: 10.1002/ece3.73370 (PMC13107284; doi:10.1002/ece3.73370)
Supplement: Supplementary file 18 — Figure S18: Evolutionary timetree of Anura derived from MCMCTREE in PAML, utilizing the concatenated nucleotide dataset that integrated 11 protein‐coding genes with all third codon positions, two rRNAs, and the concatenated 11 tRNAs (24NT dataset). The clade designations corresponded to those in Table S8. Nodes indicated by red boxes have been calibrated with prior constraint distributions, while blue boxes denoted divergences dates estimated without prior calibration constraints. The bounds of the boxes represented the 95% highest posterior density (HPD) for each node. [file ECE3-16-e73370-s029.pdf]

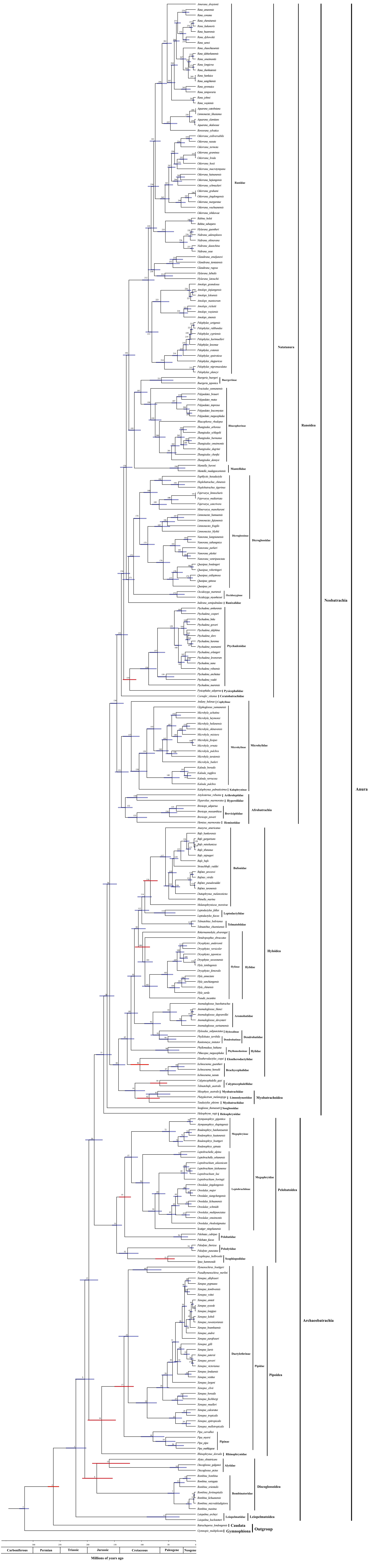

Natatanura
Ranoidea
Neobatrachia
Anura
Afrobatrachia
Hyloidea
Myobatrachoidea
Pelobatoidea
Archaeobatrachia
Pipoidea
Discoglossoidea
Leiopelmatoidea
Caudata
Gymnophiona
Outgroup
Carboniferous
Permian
Triassic
Jurassic
Cretaceous
Paleogene
Neogene
Millions of years ago
